# miR-135a-5p mediates memory and synaptic impairments via the Rock2/Adducin1 signaling pathway in a mouse model of Alzheimer’s disease

**DOI:** 10.1038/s41467-021-22196-y

**Published:** 2021-03-26

**Authors:** Kai Zheng, Fan Hu, Yang Zhou, Juan Zhang, Jie Zheng, Chuan Lai, Wan Xiong, Ke Cui, Ya-Zhuo Hu, Zhi-Tao Han, Hong-Hong Zhang, Jian-Guo Chen, Heng-Ye Man, Dan Liu, Youming Lu, Ling-Qiang Zhu

**Affiliations:** 1 Department of Geriatrics, Tongji Hospital, Tongji Medical College, Huazhong University of Science and Technology, Wuhan, China; 2 Department of Pathophysiology, Key Lab of Neurological Disorder of Education Ministry, School of Basic Medicine, Tongji Medical College, Huazhong University of Science and Technology, Wuhan, P. R. China; 3 Beijing Key Laboratory of Aging and Geriatrics, National Clinical Research Center for Geriatric Disease, Institute of Geriatrics, Chinese PLA General Hospital and Chinese PLA Medical Academy, Beijing, P. R. China; 4 The Institute of Brain Research, Collaborative Innovation Center for Brain Science, Huazhong University of Science and Technology, Wuhan, P. R. China; 5 Department of Biology, Boston University, Boston, MA USA

**Keywords:** Cognitive neuroscience, Diseases of the nervous system

## Abstract

Aberrant regulation of microRNAs (miRNAs) has been implicated in the pathogenesis of Alzheimer’s disease (AD), but most abnormally expressed miRNAs found in AD are not regulated by synaptic activity. Here we report that dysfunction of miR-135a-5p/Rock2/Add1 results in memory/synaptic disorder in a mouse model of AD. miR-135a-5p levels are significantly reduced in excitatory hippocampal neurons of AD model mice. This decrease is tau dependent and mediated by Foxd3. Inhibition of miR-135a-5p leads to synaptic disorder and memory impairments. Furthermore, excess Rock2 levels caused by loss of miR-135a-5p plays an important role in the synaptic disorder of AD via phosphorylation of Ser726 on adducin 1 (Add1). Blocking the phosphorylation of Ser726 on Add1 with a membrane-permeable peptide effectively rescues the memory impairments in AD mice. Taken together, these findings demonstrate that synaptic-related miR-135a-5p mediates synaptic/memory deficits in AD via the Rock2/Add1 signaling pathway, illuminating a potential therapeutic strategy for AD.

## Abstract (web summary)

Several micro RNAs have been shown to be deregulated in brain tissue or sera from individuals with Alzheimer’s disease and in AD mouse models. The authors show that miR-135a-5p is downregulated in excitatory pyramidal neurons from AD mice and that dysfunction of miR-135a-5p/Rock2/Add1 results in memory/synaptic disorder in AD.

## Introduction

Alzheimer’s disease (AD) is the most common neurodegenerative disorder in aged people with an increasing incidence with age in the population worldwide^1^. The best characterized clinical manifestation of AD is progressive cognitive decline, which appears at the very early stages of AD, in the absence of the two pathological hallmarks: senile plaques and neurofibrillary tangles^2,3^. Although the underlying mechanisms involved in the two pathological changes have been well studied, therapeutic strategies targeting these pathologies have failed. To date, there have been no approved drugs to block the decline in cognitive function in AD.

Synapses are considered the fundamental units in the brain, and synaptic activity can stimulate the maturation of mushroom-shaped spines and form new synapses^4^ such that synaptic strength can be adapted to environmental changes and in turn play an important role in learning and memory^5,6^. In the brain of an individual with AD, both the disruption of synaptic activity and loss of synapses were found, especially at the early disease stage^7^. Compared with senile plaques and neurofibrillary tangles, synaptic disorders, characterized by loss of synapses and decreased synaptic activity, have higher correlations with cognitive impairments in AD^8^. Thus, understanding the underlying mechanisms of synaptic disorders will be beneficial to the development of therapeutic strategies for AD at an early stage.

MicroRNAs (miRNAs) are a group of small noncoding RNAs ~22 bp in length that silence targeted genes at the posttranscriptional level^9^. miRNAs participate in many physiological processes and pathological pathways, including embryonic development, tumorigenesis, and cardiac diseases^9,10^. The fine-tuning ability of miRNAs permits them to regulate the local translation of synaptic-associated proteins at synapses^11,12^. In brain tissue or sera from individuals with AD, numerous miRNAs have been identified to be deregulated and proposed to be involved in the formation of senile plaques and neurofibrillary tangles^13^. Recently, the abnormal regulation of miRNAs in the synaptic disorder of AD was also investigated. Some brain-enriched miRNAs, such as miR-124, miR-125b, and miR-132^12,14,15^, were aberrantly expressed in AD brains and mediated impairments to synaptic plasticity. However, most of these miRNAs expression levels are not themselves regulated by synaptic activity. Thus, how these miRNAs contribute to synaptic disorders in AD remains elusive.

Here, we first screened for alterations in the levels of miRNAs associated with synaptic plasticity in the hippocampus of AD model mice and found that miR-135a-5p is abnormally downregulated in excitatory pyramidal neurons. The loss of miR-135a-5p is tau dependent and mediated by the reduction of the transcriptional factor Forkhead box D3 (Foxd3). Artificial inhibition of miR-135a-5p results in synaptic disorder and memory impairments via activation of the Rho-associated coiled-coil containing protein kinase 2 (Rock2)/Adducin 1 (Add1) signaling pathway. Overexpressing miR-135a-5p or silencing Rock2 not only rescues dendritic spine maturation but also attenuates synaptic disorders and memory deficits in AD model mice. Finally, a peptide that can block the direct phosphorylation of Add1 exerts similar neuroprotective effects, which provides the basis for developing a novel therapeutic approach for AD.

## Results

### Alterations in synaptic activity-dependent miRNAs in the hippocampus of AD model mice

**Fig. 1:**
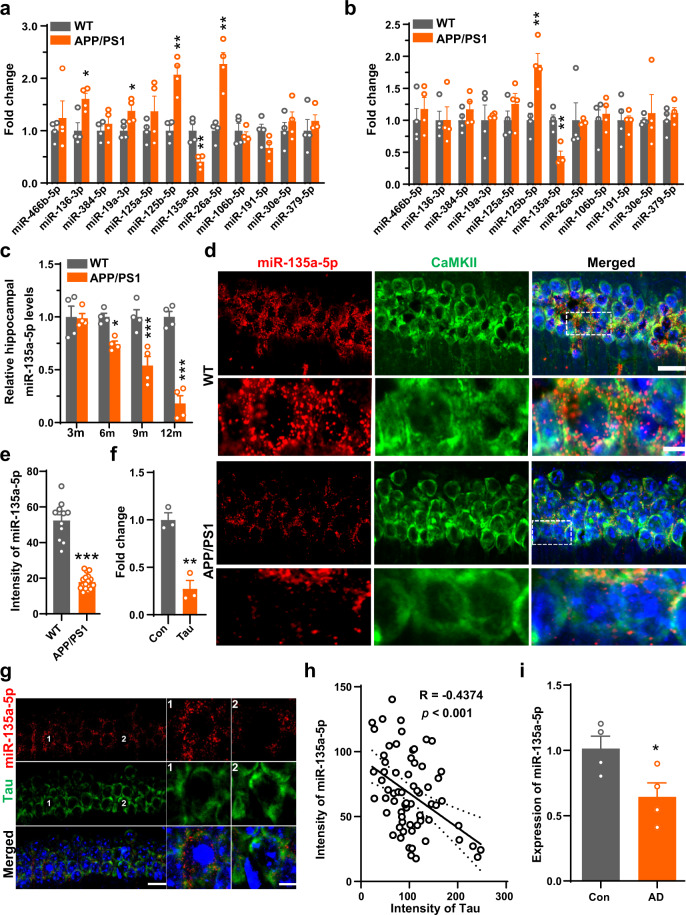
Alteration of synaptic activity-associated miRNAs in the hippocampus of AD mice. **a**, **b** Fold changes of synaptic activity associated with microRNAs (miRNAs) in the hippocampus of 12-month-old (**a**) or 9-month-old (**b**) APP/PS1 and control mice (WT) (*n* = 4 for each group). **c** Relative expression of miR-135a-5p in the hippocampus of APP/PS1 and control mice (WT) at different months as indicated (*n* = 4 for each group, two-way ANOVA, *p* < 0.0001). **d** The distribution of miR-135a-5p in glutamatergic excitatory neurons in hippocampal slices of 9 months APP/PS1 and control mice (WT) by co-immunofluorescence experiments with the antibody of CamKII (Green) and FISH of miR-135a-5p (Red). The amplification images were shown at the bottom of each group. Scale bar = 20 μm (upper), 5 μm (lower) (*n* = 3 for each group). **e** Quantification of fluorescence intensity for miR-135a-5p in CaMKII positive neurons from (**d**) (*n* = 11, 17 for WT, APP/PS1, *p* < 0.0001). **f** qRT-PCR was used to evaluate the expression of miR-135a-5p in the primary culture neurons at DIV 10 after overexpression of AAV-hTau (Tau) or control virus (Con) for 72 h (*n* = 3 for each group). **g** A representative immunofluorescence staining of FISH for miR-135a-5p (Red) and immunofluorescence experiments with the antibody of Tau-5 (Tau, Green) in hippocampus of 9 months APP/PS1 mice. Scale bar = 20 μm (left), 5 μm (right). **h** The fluorescence correlation of intensities of miR-135a-5p and Tau-5 in APP/PS1 mice as shown in panel (**g**) (*n* = 68 neurons from 5 mice). **i** The expression of miR-135a-5p in the frontal cortex from patients with AD and age-matched controls was measured by qRT-PCR (*n* = 4 for each group). (Data are presented as mean ± S.E.M. and two-tailed *t* tests were used unless otherwise specified. Source data are provided as a Source Data file. *p < 0.05, **p < 0.01, ***p < 0.001 vs. WT/Con).

To understand the potential role of synaptic activity-associated miRNAs in the pathogenesis of AD, we first performed a systematic analysis of the expression profile of synaptic activity-related miRNAs in the hippocampus of AD model mice at different stages. We focused on 12 miRNAs related to synaptic plasticity as reported in previous literature^16,17^ and compared the expression of those miRNAs in the hippocampus of APP/PS1 AD model mice to that of wild-type mice at 9 months and 12 months. We found that at 12 months of age, the AD model mice showed apparent downregulation of miR-135a-5p and upregulation of miR-136-3p, miR-19a-3p, miR-125b-5p, and miR-26a-5p (Fig. 1a). However, at 9 months of age, only a decrease in miR-135a-5p and an increase in miR-125b-5p were detected (Fig. 1b). These data suggested that miR-135a-5p downregulation or miR-125b-5p upregulation may play an important role in the relatively early stages of AD. As the dysfunction of miR-125b-5p in AD has been well documented^14^, we focused on the possible role of miR-135a-5p in the synaptic disorder of AD in this work.

### The miR-135a-5p expression is abnormally reduced in the hippocampus of AD model mice and the human frontal cortex of patients with AD

We further examined changes in miR-135a-5p levels in the hippocampus of APP/PS1 mice of different ages. We found that the level of miR-135a-5p was not changed at 3 months in APP/PS1 mice but showed a decrease at 6 months (Fig. 1c). We also performed fluorescence in situ hybridization and detected that the decrease in miR-135a-5p expression is prominent in CaMKII-positive neurons (Fig. 1d, e) but not in GAD67-positive neurons (Supplementary Fig. 1a), suggesting that the loss of miR-135a-5p in AD mainly occurs in excitatory neurons. In the cultured primary neurons, the miR-135a-5p level was reduced upon overexpression of human Tau, APP, or PS1 (Fig. 1f, Supplementary Fig. 1b, c). However, in primary neurons from Tau knockout mice, only Tau overexpression could induce the reduction in miR-135a-5p (Supplementary Fig. 1d, e). In line with this, downregulation of miR-135a-5p was also detected in the hippocampus of P301S tau mice at 6 months (Supplementary Fig. 1f). Moreover, the extent of Tau expression was negatively correlated with miR-135a-5p levels in the hippocampus of AD model mice (Fig. 1g, h) but not the wild-type mice (Supplementary Fig. 1g, h). These data suggested that the loss of miR-135a-5p in AD is tau dependent, which leads to suppressed synaptic strength (Supplementary Fig. 1i–k). Finally, in the frontal cortex of patients with AD and AD mice, the level of miR-135a-5p is decreased when compared to age-matched control subjects (Fig. 1i, Supplementary Fig. 1l).

### Loss of miR-135a-5p expression is mediated by the reduction of Foxd3

**Fig. 2:**
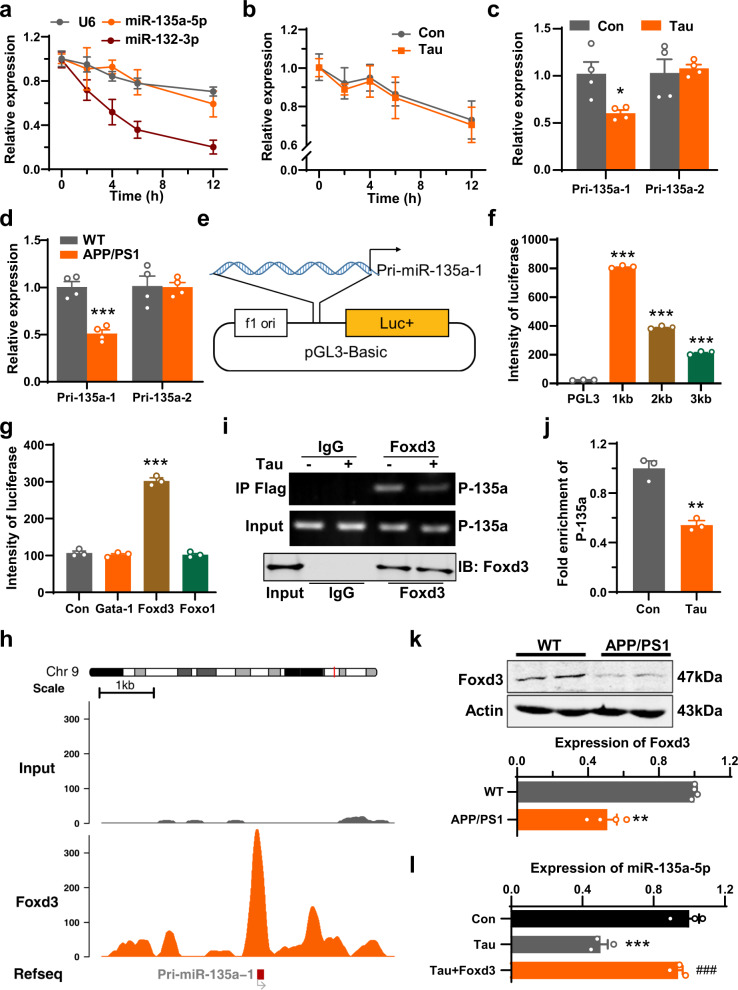
Loss of miR-135a-5p in AD is caused by the reduction of Foxd3. **a** The degradation curve of miR-135a-5p, miR-132-3p, and U6 were measured by qRT-PCR in primary hippocampal neurons at DIV 7 after treated with 10 μg/mL actinomycin D for 0–12 h (*n* = 4 for each group). **b** The degradation curve of miR-135a-5p in primary neurons after infection of hTau (Tau) or control virus (Con) (*n* = 4 for each group). **c**, **d** Alterations of pri-miR-135a-1/2 in primary neurons after infection of hTau (Tau) and control virus (Con) (**c**) or in hippocampus of 9 months APP/PS1 and control mice (WT) (**d**) (*n* = 4 for each group). **e** A diagram for the construct of pri-miR-135a-1 promoter-luciferase reporter vectors with different lengths of promoter regions (1–3 kb). **f** pri-miR-135a-1 promoter vectors were co-transfected into HEK293T cells with pRL-TK vector (Renilla luciferase reporter vector). After 48 h, the luciferase activity was analyzed (*n* = 3 for each group, one-way ANOVA, *p* < 0.0001, Dunnett’s post hoc *p* < 0.0001 vs pGL3). **g** The pGL3-1kb was co-transfected into HEK293T with transcriptional factors (Gata-1, Foxd3, Foxo1), and the luciferase activity was analyzed at 48 h later (*n* = 3 for each group, one-way ANOVA, *p* < 0.0001, Dunnett’s post hoc *p* < 0.0001 vs. Con). **h** ChIP-seq peaks showed the binding site of Foxd3 in the promoter of pri-miR-135a-1 through Integrative Genomics Viewer (IGV) for genomic regions chr9: 106151716-106155805. **i**, **j** Primary hippocampal neurons at DIV 5 was infected with AAV-hTau (Tau) or control virus (Con). One week later, neurons were infected with AAV-Foxd3-Flag. The cell lyses were immunoprecipitated with anti-Flag and then primers of the pri-miR-135a-1 promoter (P-135a) were used for PCR assay. The representative DNA gels and blots were shown in (**i**) and quantitative analysis was shown in (**j**) (*n* = 3 for each group, *p* = 0.0016). **k** The protein level of Foxd3 from hippocampus of 9 months APP/PS1 and control mice (WT) was measured by western blotting (upper panel) and quantitative analysis was performed (lower panel) (*n* = 4 for each group, *p* = 0.0019). **l** The expression of miR-135a-5p in primary neurons infected with AAV-Foxd3, AAV-hTau or control virus was detected by qRT-PCR (*n* = 3 for each group, one-way ANOVA, *p* = 0.0003, Tukey’s post hoc, Tau vs. Con *p* = 0.0004, Tau + Foxd3 vs. Tau *p* = 0.0007) (Data are presented as mean ± S.E.M. and two-tailed *t* tests were used unless otherwise specified. Source data are provided as a Source Data file. *p < 0.05, **p < 0.01, ***p < 0.001 vs. WT/Con if without specific explain.).

We then investigated why miR-135a-5p is downregulated in AD. Previous studies have shown that RNA degradation is increased in the brains of individuals with AD^18^, and we first predicted that the loss of miR-135a-5p is caused by RNA degradation. To test this hypothesis, we examined the stability of miR-135a-5p by blocking new RNA synthesis with actinomycin D^19^ and measuring the loss of miR-135a-5p and U6 levels over a 12-h period in primary hippocampal neurons infected with adeno-associated virus (AAV) containing human Tau or empty vector (Fig. 2a, b). We found that the half-life of miR-135a-5p in Tau-overexpressing neurons was identical to that in control virus-infected neurons (Fig. 2b). Our data are reliable because the half-life of miR-132-3p in our system was approximately 4 h (Fig. 2a), which is consistent with a previous study^20^. Thus, the suppression of miR-135a-5p by Tau overexpression was not caused by dysfunction in RNA degradation. Next, we considered whether the transcription of miR-135a-5p is suppressed in AD. To address this, we measured the levels of primary miR-135a-5p transcript (pri-miR-135a-1/2) in the nuclear fractions of primary hippocampal cultures with Tau overexpression. We found that pri-miR-135a-1 but not pri-miR-135a-2 was reduced in Tau-overexpressing neurons (Fig. 2c). Consistent with that result, pri-miR-135a-1 was reduced in the hippocampus of APP/PS1 AD model mice from 6 months (Fig. 2d, Supplementary Fig. 2a, b). These data suggested that the transcription of pri-miR-135a-1 is inhibited, which possibly leads to the loss of miR-135a-5p in AD. To determine how this transcription was inhibited, we cloned different fragments (1 kb, 2 kb, 3 kb) of the promoter region of pri-miR-135a-1 and inserted them into the pGL3-reporter luciferase construct (Fig. 2e). We found that the construct containing the 1 kb promoter region exhibited the highest luciferase intensity (Fig. 2f). We further analyzed the potential transcriptional factor binding sites within the promoter region of pri-miR-135a-1 (chr9:106153125–106154124) by implementing three independent website tools (RegRNA2.0^21^, PROMO^22^, and LASAGNA^23^) and identified the transcriptional factors Max, Gata-1, Foxd3, Ahr, Arnt, Olf-1, Foxo1, and HNF-3β in the predicted results of all three databases (Supplementary Data 1–3). By examining their expression in the brain (www.uniprot.org and www.biogps.org), we observed that the expression levels of Max, Ahr, Arnt, Olf-1, and HNF-3β are very low in the brain, which rules out their potential roles in regulating the transcription of pri-miR-135a-1 in the brain. We then focused on the remaining three transcriptional factors: Gata-1, Foxd3, and Foxo1. By performing cotransfection of these three factors with the reporter construct, we determined that overexpression of Foxd3 but not of Foxo1 or Gata-1 resulted in dramatically increased luciferase activity in cells transduced with the reported construct containing the pri-miR-135a-1 promoter (Fig. 2g). Consistently, existing chromatin immunoprecipitation (ChIP)-seq data (GSE58407)^24^ indicated the physical binding of Foxd3 with the pri-miR-135a-1 promoter (Fig. 2h). The results of our ChIP assay demonstrated that Foxd3 could bind with the pri-miR-135a-1 promoter (P-135a), and the binding affinity was attenuated by Tau in vitro (Fig. 2i, j). Moreover, the protein level of Foxd3 was decreased to 53% and 57% in the hippocampus of APP/PS1 mice and P301S tau mice compared to that in wild-type littermates (Fig. 2k, Supplementary Fig. 2c). Upon overexpressing Foxd3 in primary hippocampal neurons transfected with Tau, we discovered that the level of miR-135a-5p was restored (Fig. 2l). Thus, we concluded that the loss of miR-135a-5p induced by Tau is due to the reduction of Foxd3.

### Inhibition of miR-135a-5p expression induces memory impairments in vivo

**Fig. 3:**
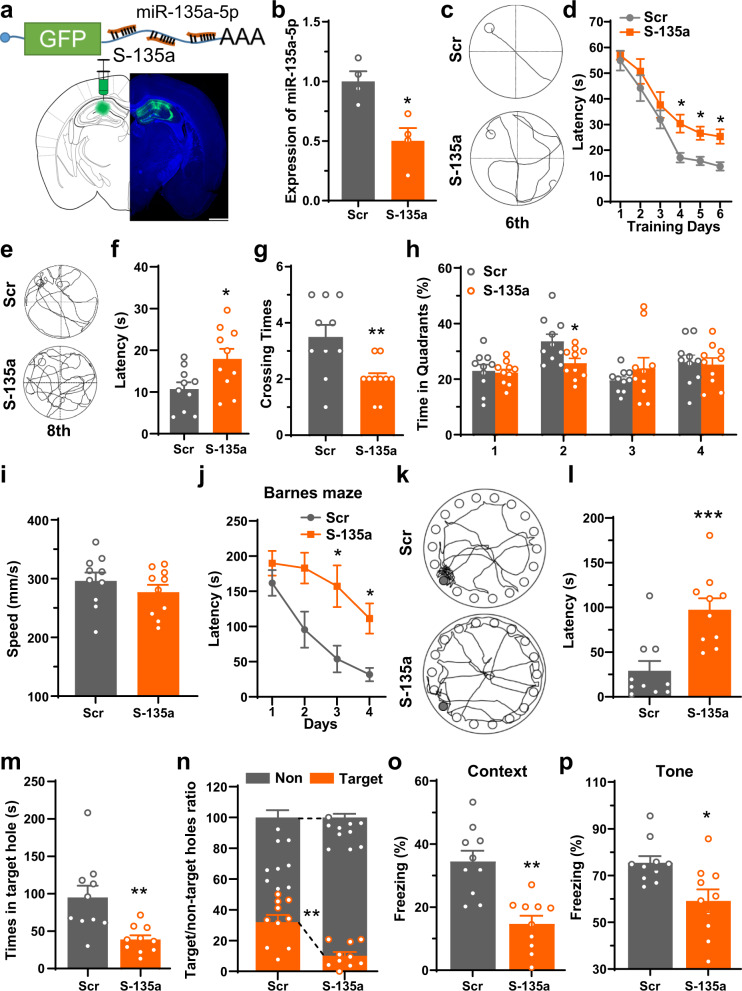
Inhibition of miR-135a-5p induces memory impairments in vivo. **a** Diagram of the AAV constructs for the miR-135a-5p sponge (S-135a) (upper panel) and a representative fluorescence image of the virus-infected slice (lower panel, *n* = 10). Bar = 1 mm. **b** qRT-PCR analyzes the expression of miR-135a-5p after injection of S-135a or scrambled sponge (Scr) to the hippocampus of C57 mice (*n* = 4 for each group). **c**–**p** The AAV packed miR-135a-5p sponge (S-135a) or scrambled control virus (Scr) were injected into the hippocampus of 6-month-old wild-type C57 mice. One month later, mice were subjected to the Morris water maze test (**c**–**i**), Barnes maze test (**j**–**n**) and fear conditioning test (**o**–**p**) to evaluate the learning and memory. **c** The representative traces to the hidden platform on day 6 of Morris water maze. **d** Latencies to the hidden platform in Morris water maze at learning stage were recorded (*n* = 10 for each group, repeated measures two-way ANOVA, *p* = 0.0087). **e** The representative traces on the probe trial at day 8. **f**–**i** Latencies to reach the previously hidden platform area (**f**), crossing times (**g**), total time spent in the different quadrants (**h**), and the swimming speed (**i**) on day 8 were analyzed (*n* = 10 for each group). **j** Latencies to the target hole for the first time were recorded during days 1–4 of the Barnes maze (*n* = 10 for each group, repeated measures two-way ANOVA, *p* = 0.0061). **k** The representative trace to reach the target hole on day 5. **l** Latencies to the target hole for the first time on day 5 (*n* = 10 for each group). **m** Time spent in nosing the target hole was evaluated (*n* = 10 for each group). **n** The accuracy of mice nosing target hole on day 5 (*n* = 10 for each group). **o** Percentage of freezing times was analyzed 24 h after contextual fear training (*n* = 10 for each group). **p** Percentage of freezing times was analyzed 48 h after fear training to tone (*n* = 10 for each group). (Data are presented as mean ± S.E.M. and two-tailed *t* tests were used unless otherwise specified. Source data are provided as a Source Data file. *p < 0.05, **p < 0.01, ***p < 0.001 vs. Scr).

We then asked whether the loss of miR-135a-5p expression is involved in the pathogenesis of AD. Because the decrease of miR-135a-5p was first detected in the 6-month AD mice, we then performed bilateral intrahippocampal injection of an AAV-miR-135a-5p (1 × 10^12^) sponge (S-135a) to simulate the loss of miR-135a-5p in the hippocampus of 6-month wild-type C57BL/6 mice (Fig. 3a, b). We evaluated spatial memory and synaptic plasticity of these mice one month later and observed that the S-135a-infected mice displayed longer latency from the fourth day during the learning stage in the Morris water maze task (Fig. 3c, d). During the probe trial test, compared with the control mice, S-135a-infected mice showed longer latency for the first crossing, fewer crossings to the platform region, and shorter swimming time in the target quadrant with no changes in swimming speed (Fig. 3e–i, the original results were shown in Supplementary Fig. 3). Furthermore, in the Barnes maze task, S-135a-infected mice displayed fewer nose poke numbers within the target hole, longer latency to the target hole, and more errors made during the search stages for the target hole than control mice (Fig. 3j–n). Finally, in the fear conditioning test, S-135a-infected mice showed a significant reduction in freezing time in the contextual conditioning paradigms, while only a slight difference was detected in tone conditioning (Fig. 3o, p), which relies on the ventral part of the hippocampus^25,26^. Together, these results indicated that loss of miR-135a-5p expression induced Alzheimer-like learning and memory deficits in mice.

### Inhibition of miR-135a-5p induces synaptic disorder in vivo

**Fig. 4:**
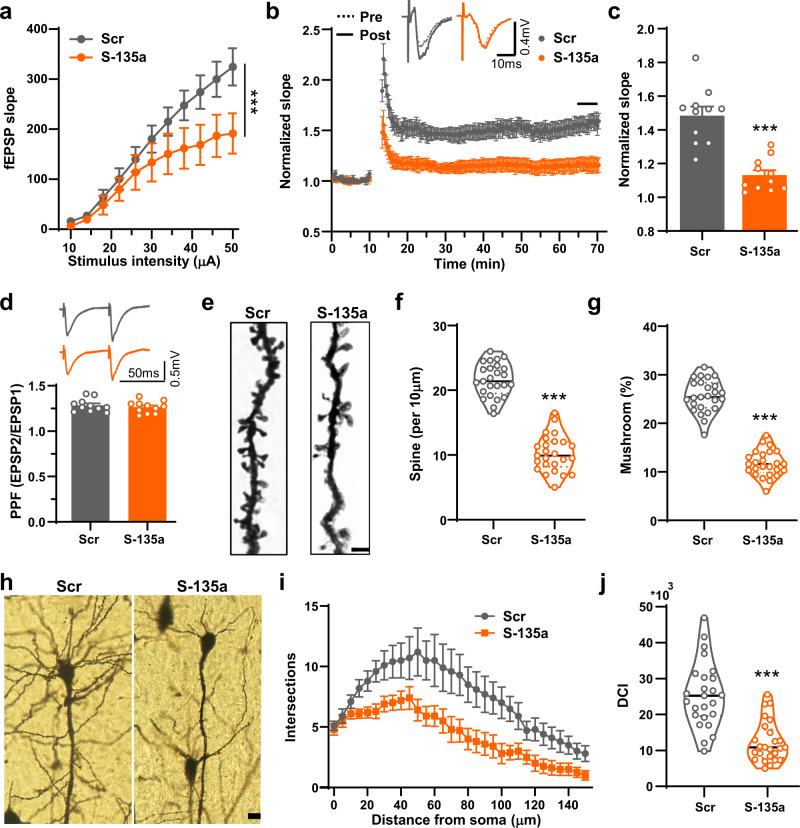
Inhibition of miR-135a-5p induces the synaptic disorder in vivo. **a** The input–output (I–O) curve between stimuli intensity and fEPSP slope in the CA3–CA1 projection of hippocampus in C57 mice after injection with S-135a or Scr virus as described above (*n* = 11 slices from 5 mice for each group, two-way ANOVA, *p* < 0.0001). **b** The normalized fEPSP slope at hippocampal CA3-CA1 projection in S-135a or Scr injected mice before and after high-frequency stimulation (HFS). Representative traces of before (dash line, pre) and after (solid line, post) HFS (*n* = 11 slices from 5 mice for each group). **c** Quantitative analysis of fEPSP slope at last 5 min as indicated by a black line in panel (**b**) (*n* = 11 slices from 5 mice for each group, *p* < 0.0001). **d** The paired-pulse amplitude ratio (second trace/the first trace) in CA3–CA1 projection (*n* = 11 for each group). **e** Representative Golgi staining images for the dendritic spines of C57 mice after injected with S-135a or Scr virus. Bar = 2 μm. **f**, **g** Quantitative analysis of the spine density (per 10 μm) (**f**) and percentage of mushroom spines (**g**) was performed (*n* = 25 neurons from 5 mice for each group, *p* < 0.0001). **h** Golgi staining images shown the dendritic trees in S-135a or Scr injected mice. Bar = 20 μm. **i** The Sholl analysis was performed to evaluate the dendritic complexity of S-135a or Scr injected mice (*n* = 25 neurons from 5 mice for each group). **j** Dendritic Complexity Index (DCI) was calculated to evaluate the branching complexity of S-135a or Scr injected mice (*n* = 25 neurons from 5 mice for each group, *p* < 0.0001). (Data are presented as mean ± S.E.M. and two-tailed *t* tests were used unless otherwise specified. Source data are provided as a Source Data file. ***p < 0.001 vs. Scr).

Because hippocampal synaptic plasticity is believed to be the basis of learning memory^27^, we then examined alterations in synaptic plasticity in S-135a-treated mice. We first evaluated hippocampal LTP in the CA3–CA1 circuit and found that the I–O curve in S-135a-infected mice was lower than that in control virus-infected mice (Fig. 4a), indicating that infection of S-135a altered basal synaptic transmission. In addition, S-135a-infected mice displayed impairment in LTP because the normalized fEPSP slope was dramatically suppressed (Fig. 4b, c). Moreover, paired-pulse facilitation (PPF) experiments suggested that S-135a did not impair presynaptic vesicle release (Fig. 4d). Golgi staining revealed that S-135a not only decreased the density of dendritic spines but also reduced the percentage of mushroom-type spines (Fig. 4e–g); furthermore, dendritic complexity, which was evaluated by Sholl analysis and DCI scores, was stunted in response to S-135a (Fig. 4h–j). These data suggested that the loss of miR-135a-5p expression results in synaptic disorder in vivo, which was observed in AD model mice^28^.

### Loss of miR-135a-5p expression results in the activation of the Rock2 signaling pathway

**Fig. 5:**
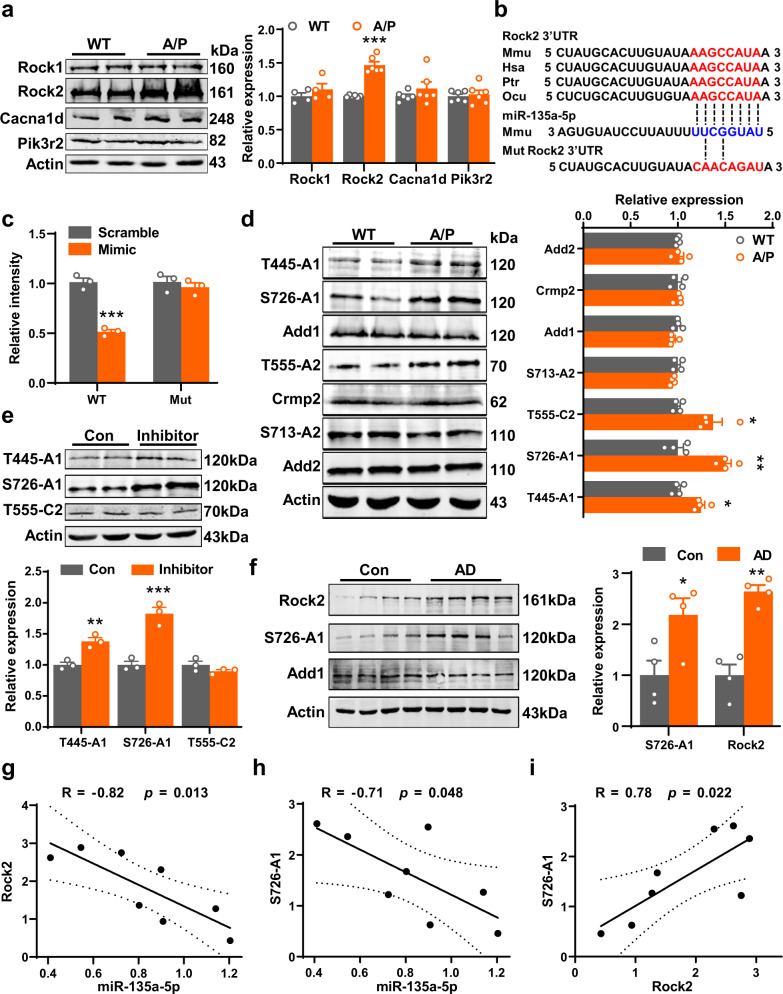
Loss of miR-135a-5p results in the activation of the Rock2 signal pathway. **a** Hippocampi of 9-month-old APP/PS1 (A/P) and wild-type (WT) mice were homogenized and western blotting was used to analyze the protein levels of Rock1, Rock2, Cacna1d, and Pik3r2 (*n* = 4 (Rock1), 6 (Others) for each group, *p* < 0.0001). **b** The sequence analysis indicated that the miR-135a-5p binding region in Rock2 3′UTR was conserved in mammalians. The mutant sequence in 3′UTR of Rock2 for luciferase analysis was provided at the bottom. **c** The wild type (WT) or mutant (Mut) 3′UTR of Rock2 in psiCHECK-2 vector was co-transfected into HEK293T cells with miR-135a-5p mimics (Mimic) or scrambled control (Scramble). The luciferase activity was determined at 48 h after the transfection (*n* = 3 for each group, two-way ANOVA, *p* = 0.0002). **d** The phosphorylation of Add1 at T445, S726 sites, Crmp2 at T555 site and Add2 at S713 site from hippocampi of 9-month-old APP/PS1 (A/P) and wild-type (WT) mice were analyzed by western blotting (*n* = 4 for each group). **e** The primary hippocampus neurons at DIV 14 were transfected with miR-135a-5p inhibitor or scrambled control for 48 h. Western blotting was used to detect the levels of phospho-T445-Add1 (T445-A1), phospho-S726-Add1 (S726-A1), and phospho-T555-Crmp2 (T555-C2) in the cell lysis (*n* = 3 for each group, *p* = 0.0032 for T445-A1, *p* < 0.0001 for S726-A1). **f** The frontal cortex from patients with AD and age-matched controls were homogenized and western blotting was used to analyze the protein levels of Rock2, phospho-S726-Add1 (S726-A1), and Add1. The representative blots were provided in the left panel and the quantitative analysis was performed as shown in right panel (*n* = 4 for each group). **g**–**i** The correlation analysis for miR-135a-5p and Rock2 (**g**), miR-135a-5p and S726-A1 (**h**), Rock2 and S726-A1 (**i**) in (**f**) and Fig. 1i (*n* = 8). (Data are presented as mean ± S.E.M. and two-tailed *t* tests were used unless otherwise specified. Source data are provided as a Source Data file. *p < 0.05, **p < 0.01, ***p < 0.001 vs. WT/Con if without specific explain).

It is known that miRNAs control gene expression by either inhibiting the translation or promoting the degradation of their target mRNAs^29^. Based on this premise, we sought to identify the direct target of miR-135a-5p that mediates synaptic disorders in AD. We used TargetScan^30^ and miRDB^31^ databases to predict the potential targets of miR-135a-5p (Supplementary Fig. 4a, Supplementary Data 4 and 5) and chose candidates with a Pct over 0.6 or miRDB scores over 70. Then, we selected 194 common candidate genes and conducted a KEGG analysis (Supplementary Fig. 4b). We found that 15 signal pathways were highly enriched, and the adjusted *p* value was less than 0.05 (Supplementary Table 1). Among the candidates in the 15 signal pathways, four of them (*Rock1*, *Rock2*, *Cacna1d*, and *Pik3r2*) were the most attractive because they exist in at least four pathways and are associated with actin cytoskeleton dynamics or calcium signaling. Given that Rock1 protein is mainly distributed in nonneuronal cells^32^ and showed no change in the hippocampus of 9-month-old APP/PS1 mice (Fig. 5a), we excluded the possible involvement of Rock1 in miR-135a-5p loss-induced memory/synaptic disorder though it plays an important role in actin remodeling and spine density^33,34^. We also detected alterations in Rock2, voltage-dependent L-type calcium channel subunit alpha-1D isoform a (encoded by *Cacna1d*) and p85β (encoded by *Pik3r2*) in the hippocampus of APP/PS1 mice. We found that only Rock2 expression was dramatically upregulated (Fig. 5a), which indicates that Rock2 is the potential downstream target that mediates the synaptic disorder induced by the loss of miR-135a-5p expression.

To verify the direct regulation of Rock2 by miR-135a-5p, we constructed the wild-type (WT) 3′UTR region of Rock2 (NM_009072.2), which contains the miR-135a-5p binding site, and a 3′UTR variant with a mutation at the miR-135a-5p binding site; cloned them both in a luciferase reporter gene and cotransfected the reporter vectors into HEK293T cells with either miR-135a-5p mimic or scrambled control (Fig. 5b). We found that the miR-135a-5p mimic suppressed the luciferase intensity in cells transfected with the WT construct but not the mutated construct (Fig. 5c). Meanwhile, by transfecting the mimic and inhibitor of miR-135a-5p into Neuro2a cells, we found that the protein levels of Rock2 were downregulated and upregulated, respectively, while mRNA levels were unaffected (Supplementary Fig. 4c, e). These data suggested the direct posttranscriptional regulation of Rock2 by miR-135a-5p. More importantly, we detected a negative correlation of miR-135a-5p and Rock2 in the hippocampus of APP/PS1 and C57 mice (Supplementary Fig. 4f, g).

Next, we explored the potential downstream effectors of Rock2 activation in mediating synaptic disorders in AD. Considering the above data, Rock2 activation may result in morphological changes in dendrites and dendritic spines, which depend on actin cytoskeleton remodeling. By referring to a previous report^35^, we performed bioinformatics analysis for the potential substrates of Rock2 and found that the phosphorylation of adducin 1 (Add1) proteins is potential of interest (Supplementary Table 2). By combining this information with a phospho-proteomics study of a brain from an individual with AD^36^, we selected Add1, Add2, and Crmp2 as candidates for further biological experiments. We found that in the hippocampus of APP/PS1 mice at 9 months, phosphorylation at Thr445 and Ser726 of Add1 and at Thr555 of Crmp2 were dramatically increased (Fig. 5d). However, in cultured hippocampal neurons, application of the miR-135a-5p inhibitor only markedly increased the phosphorylation of Add1 at Thr445 and Ser726, but not the Crmp2 at Thr555 (Fig. 5e), nor the phosphorylation of Limk1/2 (Supplementary Fig. 4h, i), two most important proteins downstream Rock regulating actin stabilization and remodeling^37,38^. Furthermore, in the frontal cortex of patients with AD and AD mice, the levels of phosphorylated Ser726-Add1 are increased, which are accompanied by an increase in Rock2 expression (Fig. 5f, Supplementary Fig. 4j, k). Importantly, we detected a relatively strong negative correlation of miR-135a-5p and both Rock2 and phospho-S726-Add1 but a positive correlation of Rock2 and phospho-S726-Add1 (Fig. 5g-i). Thus, the loss of miR-135a-5p expression results in aberrant Rock2 activation and the subsequent deregulation of the cellular cytoskeleton via phosphorylation of Add1 at Ser726.

### Overexpression of miR-135a-5p or silencing of Rock2 rescues memory impairments and synaptic disorders in AD model mice

**Fig. 6:**
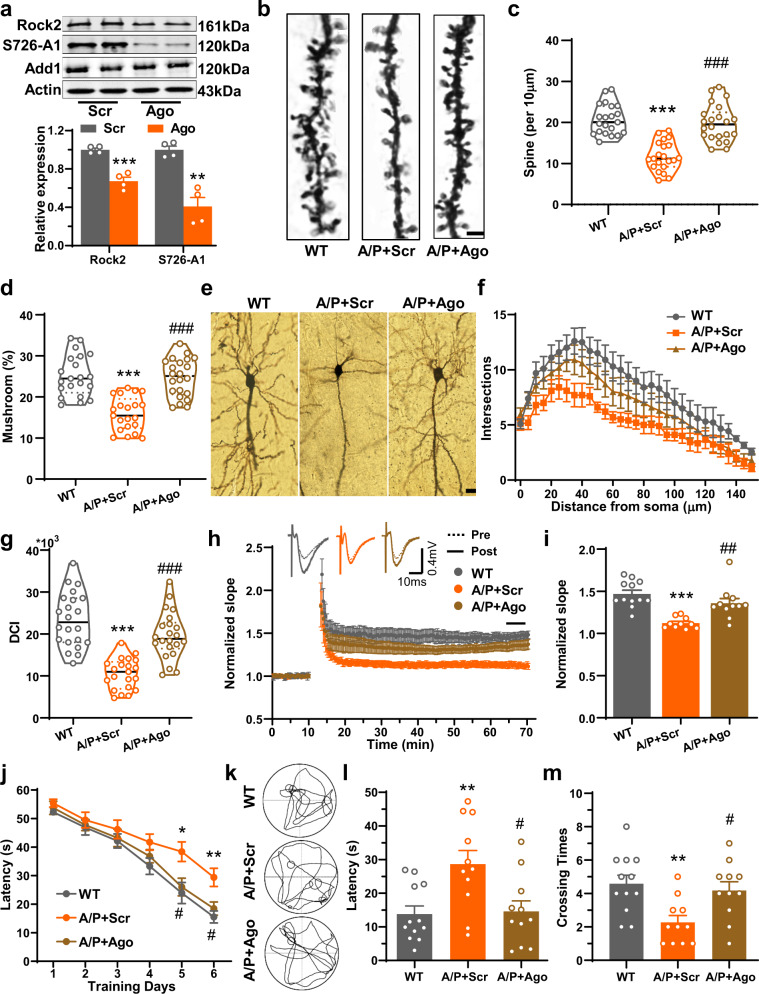
Overexpression of miR-135a-5p rescues the memory impairments and synaptic disorder in AD mice. The miR-135a-5p agomir (Ago) or scramble (Scr) was injected to CA1 region of APP/PS1 mice or wild-type littermates at 8 months old. Four weeks later, the mice were subjected to Morris water maze, electrophysiological recordings, western blotting, and Golgi staining. **a** Western blotting was used to analyze the levels of Rock2, phospho-S726-Add1 (S726-A1) in hippocampal homogenates from APP/PS1 mice with miR-135a-5p agomir (Ago) or scramble (Scr) injected (*n* = 4 for each group, **p < 0.01, ***p < 0.001 vs. Scr). **b**–**d** Golgi staining was performed to show spines density and maturation in the hippocampal CA1 region. **b** Representative images of dendritic spines. **c** The quantitative analysis for the spine density (per 10 μm) and **d** percentage of mushroom-type spines (*n* = 21, 21, 22 neurons from 5 mice for WT, A/P + Scr, A/P + Ago, one-way ANOVA, *p* < 0.0001, Tukey’s post hoc, *p* < 0.0001). WT wild type mice, A/P + Scr: APP/PS1 mice injected with scramble agomir; A/P + Ago: APP/PS1 mice injected with miR-135a-5p agomir. **e**–**g** Golgi staining was used to analyze the dendritic morphology of neurons. **e** The representative images of dendritic trees in the CA1 of mice in different groups. Bar = 20 μm. Sholl analysis (**f**) and dendritic complexity index (DCI) analysis (**g**) were performed to evaluate the dendritic complexity (*n* = 22, 21, 22 neurons from 5 mice for WT, A/P + Scr, A/P + Ago, one-way ANOVA, *p* < 0.0001, Tukey’s post hoc, *p* < 0.0001). **h** The electrophysiological recording was performed in CA3–CA1 projection for LTP analysis. Representative traces were shown in the upper panel and the normalized slope of fEPSP at different timelines were plotted in the lower panel. **i** Quantitative analysis of fEPSP slope was calculated for the last 5 minutes recording in (**h**) (*n* = 12, 10, 11 slices from 7, 6, 6, mice for WT, A/P + Scr, A/P + Ago, one-way ANOVA, *p* < 0.0001, Tukey’s post hoc, *p* < 0.0001 for A/P + Scr vs. WT, *p* = 0.003 for A/P + Ago vs. A/P + Scr). **j**–**m** The performances of all mice in the Morris water maze task. The latency at the learning stage from days 1 to 6 (**j**) was recorded. The representative traces (**k**), first time to the platform region (**l**) and crossing times (**m**) at day 8 were analyzed (*n* = 12, 11, 11 for WT, A/P + Scr, A/P + Ago, repeated measures two-way ANOVA with Tukey’s post hoc, *p* = 0.0003 for (**j**), one-way ANOVA with Tukey’s post hoc, *p* = 0.0040 for (**l**), *p* = 0.0046 for (**m**)). (Data are presented as mean ± S.E.M. and two-tailed *t* tests were used unless otherwise specified. Source data are provided as a Source Data file. *p < 0.05, **p < 0.01, ***p < 0.001 A/P + Scr vs. WT, ^#^*p* < 0.05, ^##^*p* < 0.01, ^###^*p* < 0.001 A/P + Ago vs. A/P + Scr if without specific explain).

We then asked whether upregulation of miR-135a-5p or silencing of Rock2 could rescue memory impairments and synaptic disorders in AD model mice. We used 8-month-old APP/PS1 mice for the injection, because the memory impairment was apparent in 9 months but not 6 months mice^39,40^, and the agomir of miR-135a-5p only lasts up to 6 weeks^41,42^. Upon infusion of the agomir of miR-135a-5p or the lentivirus containing Rock2-specific shRNA into the hippocampus of 8-month-old APP/PS1 mice (Supplementary Fig. 5a), we found that both agents were able to not only suppress Rock2 expression and the phosphorylation of Add1 at Ser726 (Fig. 6a, Supplementary Fig. 5a) but also restore the density and maturation of dendritic spines (Fig. 6b–d, Supplementary Fig. 5b–d) and the complexity of neuronal dendrites (Fig. 6e–g, Supplementary Fig. 5e–g). Moreover, the LTP/LTD deficits (Fig. 6h, i, Supplementary Fig. 5h, i, Supplementary Fig. 6) and memory impairments in AD model mice could also be partially rescued (Fig. 6j–m, the original data was shown in Supplementary Fig. 7, Supplementary Fig. 5j–m). These data suggested that restoring the miR-135a-5p/Rock2 signaling pathway rescues memory impairments and synaptic disorders in AD.

### A peptide blocking the phosphorylation of Add1 rescues memory impairments and synaptic disorders in AD model mice

**Fig. 7:**
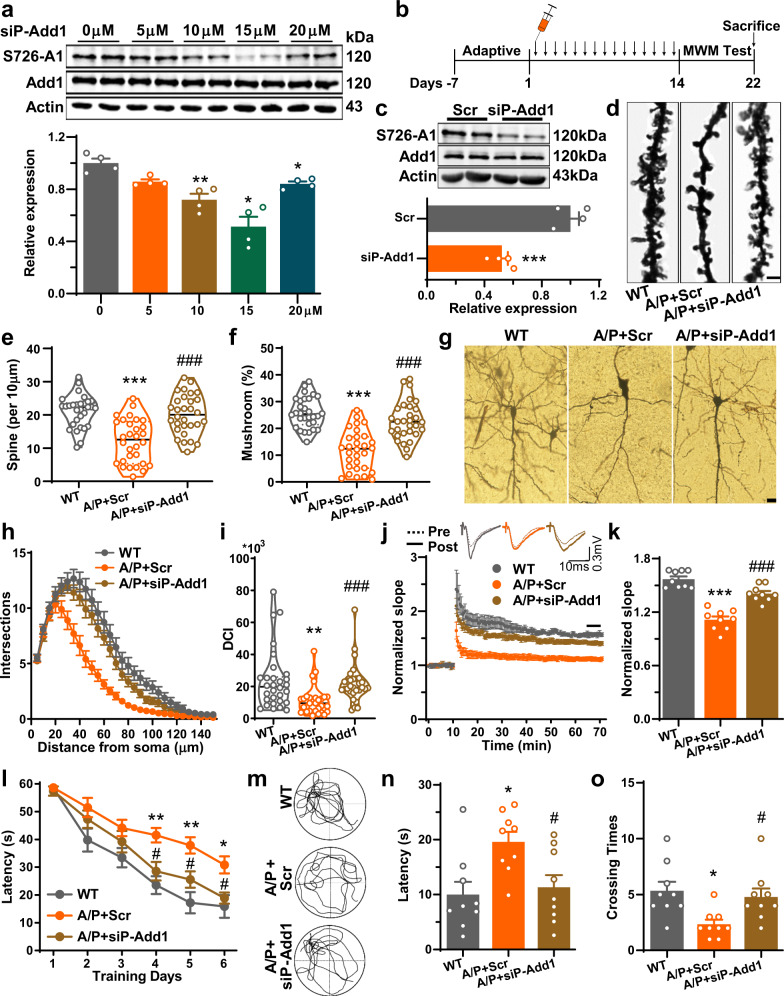
A peptide blocking the phosphorylation of Add1 rescues the memory impairment and synaptic disorder in AD mice. **a** HEK293T cells were co-transfected with Add1 and Rock2 for 24 h. Then, the cells were treated with siP-Add1 at 0–20 μM as indicated for another 3 h. The phosphorylation level of Ser726-Add1 (S726-A1) was detected by western blotting. Upper panel, the representative blots; lower panel, the quantification analysis data (*n* = 4 for each group, Welch’s one-way ANOVA, *p* = 0.0063, Dunnett’s T3 post hoc *p < 0.05, **p < 0.01 vs. 0 μM). **b** Schematic diagram for the experimental procedure. 9-month-old APP/PS1 mice were intraperitoneally injected with siP-Add1 or scrambled peptides (Scr) (15 mg/kg per day) for 2 weeks. Then mice were subjected to Morris water maze, electrophysiological recordings, western blotting, and Golgi staining. **c** Western blotting was used to analyze the expression of S726-Add1 in hippocampal homogenates from APP/PS1 injected with siP-Add1 or scrambled peptides (*n* = 4 for each group). **d**–**f** Golgi staining was used to examine the dendritic spines. The representative images for the dendritic spines in CA1 region (**d**) and quantitative analysis of the spine density (per 10 μm) (**e**) and percentage of the mushroom spine (**f**) (*n* = 30 neurons from 5 mice for each group, one-way ANOVA with Tukey’s post hoc, *p* < 0.0001). **g** The representative images of dendritic trees in the hippocampal CA1 neurons. Bar = 20 μm. **h**, **i** The Sholl analysis (**h**) and Dendritic Complexity Index (DCI) analysis (**i**) were performed to evaluate the dendritic complexity (*n* = 30 neurons from 5 mice for each group, Welch’s one-way ANOVA with Dunnett’s T3 post hoc, *p* = 0.0001). **j**, **k** The electrophysiological recording was performed to examine the LTP in the CA3–CA1 projections. Representative traces and normalized fEPSP slopes were shown in (**j**). The quantitative analysis (**k**) was calculated from last 5 min recording (*n* = 9 slices from 4 mice for each group, one-way ANOVA, *p* < 0.0001, Tukey’s post hoc, *p* < 0.0001). **l**–**o** The performances of mice in Morris water maze. Latencies in the learning stage from days 1–6 were recorded (**l**). The representative traces (**m**), first time to the platform region (**n**) and crossing times (**o**) at day 8 were analyzed and shown (*n* = 9 for each group, repeated measures two-way ANOVA with Tukey’s post hoc, *p* < 0.0001 for (**l**), one-way ANOVA with Tukey’s post hoc, *p* = 0.0082 for (**n**), *p* = 0.0106 for (**o**)). (Data are presented as mean ± S.E.M. and two-tailed *t* tests were used unless otherwise specified. Source data are provided as a Source Data file. *p < 0.05, **p < 0.01, ***p < 0.001 A/P + Scr vs. WT, ^#^*p* < 0.05, ^##^*p* < 0.01, ^###^*p* < 0.001 A/P + siP-Add1 vs. A/P + Scr).

Finally, we tested whether blocking the phosphorylation of Add1 can rescue memory impairments and synaptic disorders in AD model mice. According to our previous experience^43^, we generated a series of peptides of different lengths that overlap the phosphorylation sites (Supplementary Fig. 8a). We found that only peptide 3 could attenuate the phosphorylation of Add1 in vitro (Fig. 7a, Supplementary Fig. 8b); thus, we named this peptide siP-Add1 and administered it to 9-month-old APP/PS1 or wild type mice at a dose of 15 mg/kg for 14 consecutive days via *i.p*. injection (Fig. 7b). We observed that siP-Add1 had no effect on WT mice, including the bodyweight, open field test, dendritic spines and trees, and LTD (Supplementary Fig. 8c-m). Our results showed reduced levels of phosphorylated S726-Add1 in vivo (Fig. 7c). Moreover, siP-Add1 administration not only significantly restored the density of dendritic spines (Fig. 7d–f) and the complexity of neuronal dendrites (Fig. 7g-i) but also ameliorated LTP and LTD deficits (Fig. 7j, k, Supplementary Fig. 8k–m) and memory impairments (Fig. 7l–o) in AD model mice. Thus, blocking Rock2-mediated phosphorylation of Add1 effectively rescues AD-like synaptic and memory impairments in mice.

## Discussion

**Fig. 8:**
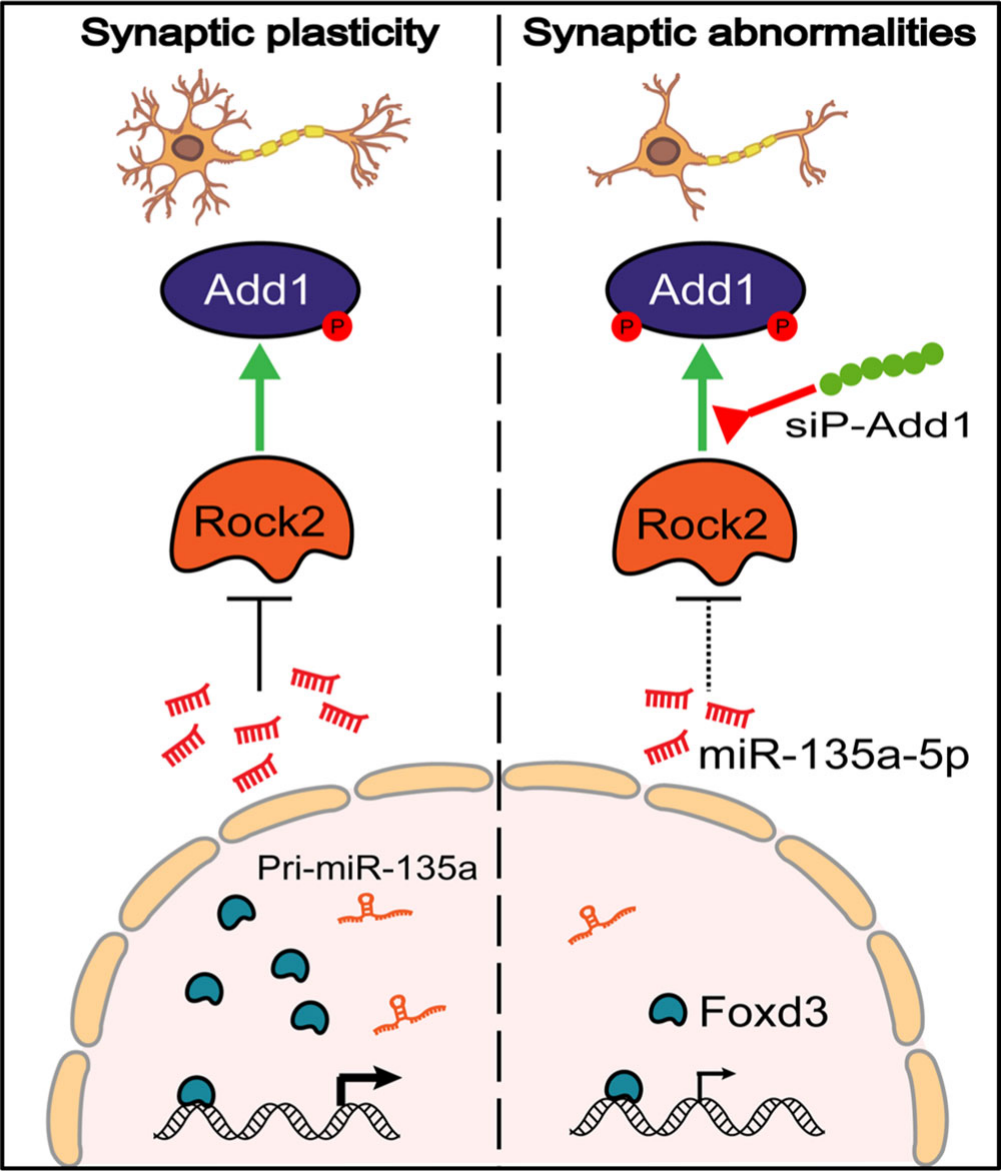
miR-135a-5p/Rock2/Add1 signal mediates the synaptic/memory impairments in AD. In normal conditions (left), miR-135a-5p was transcriptionally regulated by Foxd3 and suppressed the Rock2 expression, which was essential for normal synaptic plasticity. In AD (right), loss of Foxd3 results in the downregulation of miR-135a-5p and the overexpression of Rock2, which in turn caused the hyperphosphorylation of Add1 in the Ser726 site and the aberrant dendritic morphology, as well as the synaptic/memory impairments. Blocking the hyperphosphorylation of Add1 by siP-Add1 partially rescued those abnormalities.

In this study, we identified that miR-135a-5p, a synaptic-associated miRNA, is abnormally downregulated in AD. Loss of miR-135a-5p expression results in an increase in Rock2 activity and hyperphosphorylation of Add1 at Ser726, which in turn leads to dendritic abnormalities and memory impairments. Blocking the miR-135/Rock2/p-Add1 axis effectively rescued the AD-like synaptic and memory deficits in the APP/PS1 mouse model (Fig. 8).

Recently, we have demonstrated the critical roles of multiple miRNAs in mediating the synaptic disorders of AD^12,44,45^, but most of them are not associated with synaptic activity. Here, we first screened the alterations in miRNAs associated with the synaptic activity, and of those miRNAs, miR-135a-5p was of particular interest because it showed the most dramatic change in expression in the hippocampus of AD model mice at 9 months. Although the expression profile of miR-135a-5p in the hippocampal of patients with AD is not clear, some of the high throughout data suggested that miR-135a-5p was downregulated in the gray matter and serum of AD paitents^46,47^. miR-135a-5p is upregulated by long-term depression (LTD), a form of synaptic plasticity proposed as the primary cellular substrate of learning/memory^48^. Our data demonstrated that the downregulation of miR-135a-5p in AD is tau dependent because the loss of miR-135a-5p expression in *MAPT* KO neurons can be induced by tau overexpression but not by either APP or PS1. In line with this, tau pathology (hyperphosphorylation of Tau) in the hippocampus and cortex of APP/PS1 mice occurs no earlier than 6 months^49,50^, at which point miR-135a-5p levels began to decrease and the mice displayed significant synaptic disturbance. Soluble tau aggregates inhibit synaptic LTD in vivo^51^, and Tau is required for the synaptic toxicity of Aβ^52^. Therefore, tau pathology might play a critical role in mediating miR-135a-5p suppression in AD. Moreover, we also found no negative correlation of Tau with miR-135a-5p in normal mice, suggesting that the synergism of Aβ and Tau^53^ might be important to mediate the downregulation of miR-135a-5p in AD. In addition, we found that the decrease in miR-135a-5p expression is prominent in excitatory neurons and caused by a decrease in Foxd3 levels in AD. Foxd3 is a well-known transcriptional factor that is important for the development of the vertebrate nervous system, including the determination, migration, and differentiation of neural crest lineages^54^. There are many synaptic-related (or brain-enriched) genes, including many miRNAs^55^ that are transcriptionally regulated by Foxd3^56^. Importantly, a study of genome-wide identification of the binding region of Tau in neurons found that an AG-rich motif was present within Tau-binding regions, and Tau acted as a repressor of genes related to Tau-binding^57^. Meanwhile, we found that two of the variant AG-rich motif (GGAGGGAGAG, GGAGAGAGAG) were present in the promoter region of Foxd3 at −3807 to −3793 in the mouse genome. It is thus possible that Tau protein especially the pathological Tau could bind and repress the transcription of Foxd3, resulting in a reduction of Foxd3 protein. Consistently, the direct transcriptional regulation of miR-135a-5p by Foxd3 was identified in our study based on a series of experiments and previous array data. Thus, the loss of miR-135a-5p expression in AD is Tau dependent and mediated by Foxd3.

Numerous reports have revealed the crucial roles of miRNAs in controlling neuronal morphology and maintaining synaptic plasticity^58^. An image-based miRNome-wide functional miRNA screen in an induced neuronal differential cell model suggested that miR-135a-5p is the potent stimulator for developmental axon growth and branching, cortical neuronal migration, and axon regeneration^59^. Intravitreal injection of miR-135a/b mimic promoted the axon regeneration of retinal ganglion cell (RGC) after optic nerve injury in adult mice. While miR-135a/b sponge further destroyed the axon regeneration of RGC. This was consistent with our result that miR-135a-5p promoted dendritic complexity of hippocampal pyramidal neurons. Administration of miR-135a inhibitors effectively blocked NMDA-induced spine retraction^16^. These lines of evidence strongly suggest that miR-135a-5p deregulation is correlated with abnormalities in neuronal morphology. In this study, we found that the loss of miR-135a-5p expression results in a decrease in dendritic complexity and spine maturation, while overexpression of miR-135a-5p in vivo rescues those abnormalities. Moreover, loss of miR-135a-5p expression induces the reduction in LTP and basal transmission in the CA3–CA1 projection. This inhibition seems to be a postsynaptic process because we did not observe any differences in the PPF experiment. However, a recent study reported that overexpression of pre-miR-135a leads to a significant reduction in the number of spines in mouse primary hippocampal neurons^60^. We propose that this discrepancy is due to the different circumstances between the in vitro and in vivo experiments, and the CMV or constitutively active mutant Mef2-vp16 driven overexpression of pre-miR-135a would overwhelm the endogenous miRNA processing machinery^61,62^ and result in significant increases of both miR-135a-5p and miR-135a-1/2-3p, which is different from our findings here showing upregulation of miR-135a-5p alone. Similar to our research, Liu et al.^63^ reported decreased miR-135a-5p in the hippocampus of APP/PS1 mice and suggested that this is responsible for the overproduction of Aβ, the prime contributor to the synaptic disorder in AD. In another study, miR-135a-5p knockdown enhances the frequency of sEPSC events in BLA pyramidal neurons^64^, suggesting a regulatory role of miR-135a-5p in presynaptic function in the amygdala. Here, we demonstrated the critical role of miR-135a-5p in regulating postsynaptic maturation in the hippocampus. Interestingly, targeted knockdown of miR-135a-5p in the amygdala results in anxiety-like behavior in mice^64^. As anxiety is one of the most prevalent psychiatric manifestations in the early stage of AD, administration of miR-135a-5p might be a therapeutic approach for both emotional and cognitive disorders relating to AD. Besides, a series of important studies have demonstrated several other microRNAs, including miR-132-3p, were differentially expressed in response to LTP induction and regulated synaptic proteins relevant to persistent LTP^65,66^. Given the important roles of miRNA in the fine-tuning of gene regulation^67^, targeting these synaptic activity-regulated miRNAs might be promising therapeutic approaches for synaptic disorder-related neuropsychiatric diseases.

Our study further demonstrated the important role of Rock2-mediated hyperphosphorylation of Add1 in the synaptic/memory disorder caused by reduced miR-135a-5p expression in AD. It is well known that posttranscriptional regulation by miRNAs has pleiotropic effects, and it is reasonable to speculate that miR-135a-5p participates in synaptic/memory disorders by targeting different target mRNAs. A previous KEGG analysis in the rat hippocampus suggested that the predicted target genes of miR-135a-5p might be important for LTP and the synaptic vesicle cycle of glutamatergic synapses in AD^68^. Here, we pursued Rock2 as a direct target of miR-135a-5p based on a series of bioinformatics analyses and biochemical validation. The in vivo experiments further confirmed the remedial effects of silencing Rock2 on dendritic abnormalities and synaptic/memory deficits. Our study demonstrated that the miR-135a-5p/Rock2 signaling axis is important for actin cytoskeleton dynamics in dendrites and is consistent with a previous in vitro study^69^. Coincidentally, overexpression of miR-135a-5p results in the downregulation of Rock1 and Rock2 expression, which in turn suppresses the in vivo invasion abilities of prostate cancer cells, indicating that this pathway is also implicated in actin cytoskeleton organization in tumors^70^. Moreover, a recent screening series including quantitative proteomic screens and in silico analysis revealed Rock2 as one of six potential main regulators of synaptic and axonal degeneration in vivo^71^. Furthermore, Rock2 knockout mice displayed significant deficits in spine properties and synapse density as well as impairments in basal synaptic transmission and hippocampal LTP^72^. Our data provide evidence that overexpression of Rock2 can also impair dendritic morphology and LTP and affect basal transmission. Thus, maintaining a certain level of Rock2 is essential for physiological synaptic plasticity. We also identified the downstream targets of Rock2, such as Add1, that promote synaptic disorders, and disruption of the phosphorylation of Add1 by a specific peptide also rescued dendritic abnormalities and learning memory deficits in AD. It is known that there are many other substrates of Rock2, such as Limk, Mlc, Crmp2^73^, which mediate a variety of biological processes including cytoskeletal regulation, cellular responses and neuronal degeneration. The application of TAT peptide could specifically block the interaction between Rock2 and Add1 and phosphorylation of S726-Add1 without affecting other substrates^74,75^. This result provided evidence of a novel therapeutic approach for this devastating disease. Finally, we speculate that Rock2 might not be the sole downstream target of miR-135a-5p in AD. Our predicted miR-135a-5p targets include the following genes that are related to the disruption of neuronal morphology remodeling and learning memory in AD: glycogen synthase kinase-3 beta (*Gsk3b*), beta-site APP cleaving enzyme 1 (*Bace1*), glutamate receptor, ionotropic, NMDA2B (epsilon 2) (*Grin2b*), very low-density lipoprotein receptor (*Vldlr*), Golgi-associated, gamma adaptin ear containing, ARF binding protein 1 (*Gga1*), fermitin family member 2 (*Fermt2*), NADH:ubiquinone oxidoreductase subunit B9 (*Ndufb9*), and Ndufa4, mitochondrial complex associated (*Ndufa4*) (see Supplementary Table 3 for additional putative targets of miR-135a-5p that are associated with AD).

In summary, the results here provide evidence for an important role of the miR-135a-5p/Rock2 signaling pathway in mediating synaptic disorders in AD. As decreased miR-135a-5p levels have been observed in the gray matter of patients with AD^46^ and Rock2 levels are increased in the earliest stages of AD and remain elevated throughout disease progression, our study proposed a therapeutic target for overcoming synaptic and memory deficits in AD.

## Methods

### Animals and cells

Male APP/PS1 mice were purchased from the Jackson Laboratory (Bar Harbor, ME, NO. 034832). Adult male C57BL/6 mice were purchased from the National Resource Center of Model Mice (Nanjing, China). The Tau-Knockout (Tau−/−) mouse was generated using the CRISPR–Cas9 system by specifically knocking out the sequence of exon 5 (Beijing Biocytogen Co., Ltd., Beijing, China). Genotyping for Tau−/− mice was performed by multiplex polymerase chain reaction using a pair of primers (Forward primer: 5′-GCTACAGTGTGAGTGAGGTTCTAGC-3′; Reverse primer: 5′-GAACCACAGCTGCTTAGGGAAAAC-3′). The genetic background of Tau−/− mice is C57BL/6. All these mice and their nontransgenic littermates were bred in the Experimental Animal Central of Tongji Medical College, Huazhong University of Science and Technology. All these mice were housed under a 12-h light/dark cycle in a temperature (22–24 °C) and humidity (40–60%) controlled room with enough food and water. This study was approved by the Institutional Animal Care and Use Committee of the Huazhong University of Science and Technology. Mouse N2a cells and Human 293T cells were bought from the American Type Culture Collection (ATCC) bank (Manassas, VA, USA).

### Plasmids and viruses

miR-135a mimic/inhibitor/agomir and the scrambled control were purchased from RiboBio (Guangzhou, China). Mimic and inhibitor was used for transfection on cells, and agomir was used for stereotaxic injection in mice. 3′UTR of Rock2 was amplified and cloned into psiCHECK-2 (Promega, Madison, WI). The promoter sequence of miR-135a-1 was downloaded from UCSC, and the 3 kb, 2 kb, 1 kb sequence immediately upstream to the transcription start site were cloned into pGL3-Basic vector. The coding sequences for the mRNAs of Foxd3, Gata-1, and Foxo1 were cloned into pcDNA3.1(−) or pcDNA3.1(−)3xflag. Rock2 was cloned into a pcDNA4 vector. AAVs for miR-135a sponge and lentivirus for Rock2 short hairpin RNA was purchased from OBiO Technology (Shanghai, China). The target sequence of Rock2 shRNA was 5′-AACAATAGAGATCTACAAGAT-3′.

### Morris water maze

The Morris water maze was performed as previously described^45^. Briefly, Marks of different shapes were pasted around the swimming tank, the temperature of the water was kept between 20 and 22 °C and was made opaque with moderate titanium dioxide powder. The mice were trained for six consecutive days in the afternoon to find a platform hidden that submerged 1 cm below the water surface. Mice were trained from three different quadrants to find the platform for up to 60 s every day. Each mouse remained on the platform for 15 s if they found it or they were guided to the platform and stayed for 15 s. The movements of mice were recorded by a video camera connected to a computer. There was no task on the seventh day and mice got a rest. On the eighth day, the hidden platform was removed, mice were tested to find the platform from the opposite quadrant for 60 s (probe trial). The latency to reach the place of the platform, the percent time spent in the target quadrant, the crossing times to the platform regions, and the swimming velocity was recorded.

### Luciferase reporters assay

The pGL3 containing different promoter sequences of miR-135a was cotransfected into HEK293T cells with pRL-TK. And the vector containing 1 kb promoter of miR-135a was cotransfected with Foxd3, Gata-1 or Foxo1 in addition to pRL-TK. The psiCHECK2 containing WT or mutated (Mut) 3′UTR of Rock2 was cotransfected into HEK293T with mimic or scramble of miR-135a. Cells were harvested after 48 h and cell lysates were collected with 1× PLB buffer for firefly and renilla luciferase activities using the dual-luciferase reporter assay system (Promega, USA) according to the manufacturer’s protocol.

### Primary hippocampal neuron culture

The isolated embryonic hippocampal neurons were cultured as previously described^45^. Briefly, pregnant mice between 15 and 17 days were anesthetized and hippocampi of fetal mice were isolated under a dissection microscope at 4 °C. The collected hippocampi were cut into small pieces with scissors, digested with 4 ml of 0.125% trypsin for 10–15 min, and stopped by adding 5 ml of the neuronal planting medium (10% fetal bovine serum in DMEM/F12). After centrifugation, resuspending, and trituration with the planting medium, the neurons were filtered with a 200-mesh sieve and plated onto a 60 mm plastic culture dish or 6-well cell culture plate coated with poly d-lysine. The neurons were incubated in the humidified incubator at 37 °C for 4 h with 5% CO_2_, and the medium was replaced with a Neurobasal medium supplemented with 2% B27 (maintenance medium). The medium was changed every 3 days for half volume.

### Electrophysiological recording

The electrophysiological recording of hippocampal sections was performed as previously described^44^. Briefly, mice were decapitated and the brains were immediately immersed in ice-cold ACSF (125 mM NaCl, 2.0 mM KCl, 2.5 mM CaCl_2_, 1.2 mM MgSO_4_, 1.2 mM KH_2_PO_4_, 26 mM NaHCO_3_, and 11 mM glucose), which was continuously bubbled with 95% O_2_ and 5% CO_2_. The mouse brain was cut into 300-μm-thick transverse slices in a horizontal plane with a vibrating microtome (Leica). Slices were preincubated in ACSF at 32 °C for 30 min and then at room temperature for 30 min. A multi-electrophysiological recording setup (MED64; Alpha Med Sciences, Tokyo, Japan) was used to record the field excitatory postsynaptic potential (fEPSP). Slices were placed on 64 microelectrodes in the center of a recording chamber and perfused constantly with ACSF at 32 °C. I/O curves were obtained with an incremental stimulation intensity from 10 to 50 μA. The fEPSPs evoked at CA3-CA1 synapses were recorded from the dendritic layer of CA1 neurons. LTP was induced using a standard high-frequency stimulation paradigm consisting of 2 trains of stimuli at 100 Hz (each for 1 s, pulse duration 200 μs) with 30 s intervals. PPF was accomplished with two consecutive stimuli pulses separated by 50 ms. LTD was induced using low-frequency stimulation at 1 Hz (pulse duration 200 μs) with 900 pulses for 15 min. For mEPSCs recordings, hippocampal neurons were held at −70 mV in voltage-clamp mode and were recorded in 10 s epochs for a total duration of at least 200 s per recording^76^. The data were collected and analyzed with pClamp 10 and ClampFit 10.2, respectively. Statistical analysis was performed by using the data from the last 5 min of recording.

### Statistical analysis

All data are presented descriptively as the mean ± SEM and analyzed using SPSS 18.0 and Prism 6.0. The difference between the two groups was assessed using an unpaired Student’s *t* test. The variance among multiple groups was assessed by a one- or two-way analysis of variance with/without repeated measures followed by a post hoc test. All experiments were repeated three times except those specified, and *p* < 0.05 was considered statistically significant. All the statistical analysis data was supplied as Supplementary Table 4.

### Reporting summary

Further information on research design is available in the Nature Research Reporting Summary linked to this article.

## Supplementary information

Supplementary Information

Description of Additional Supplementary Files

Supplementary Data

Reporting Summary

## Data Availability

The source data for figures and supplementary figures of this paper are available in the source data file. Source data are provided with this paper.

## Source data

Source Data
