# Supplementary material for: miR-135a-5p mediates memory and synaptic impairments via the Rock2/Adducin1 signaling pathway in a mouse model of Alzheimer’s disease
Source: Nat Commun. 2021 Mar 26;12:1903. doi: 10.1038/s41467-021-22196-y (PMC7998005; doi:10.1038/s41467-021-22196-y)
Supplement: Supplementary file 1 — Supplementary Information [file 41467_2021_22196_MOESM1_ESM.pdf]

**miR-135a-5p mediates memory and synaptic impairments via the Rock2/Adducin1 signaling pathway in a mouse model of Alzheimers disease**

## SUPPLEMENTARY INFORMATION

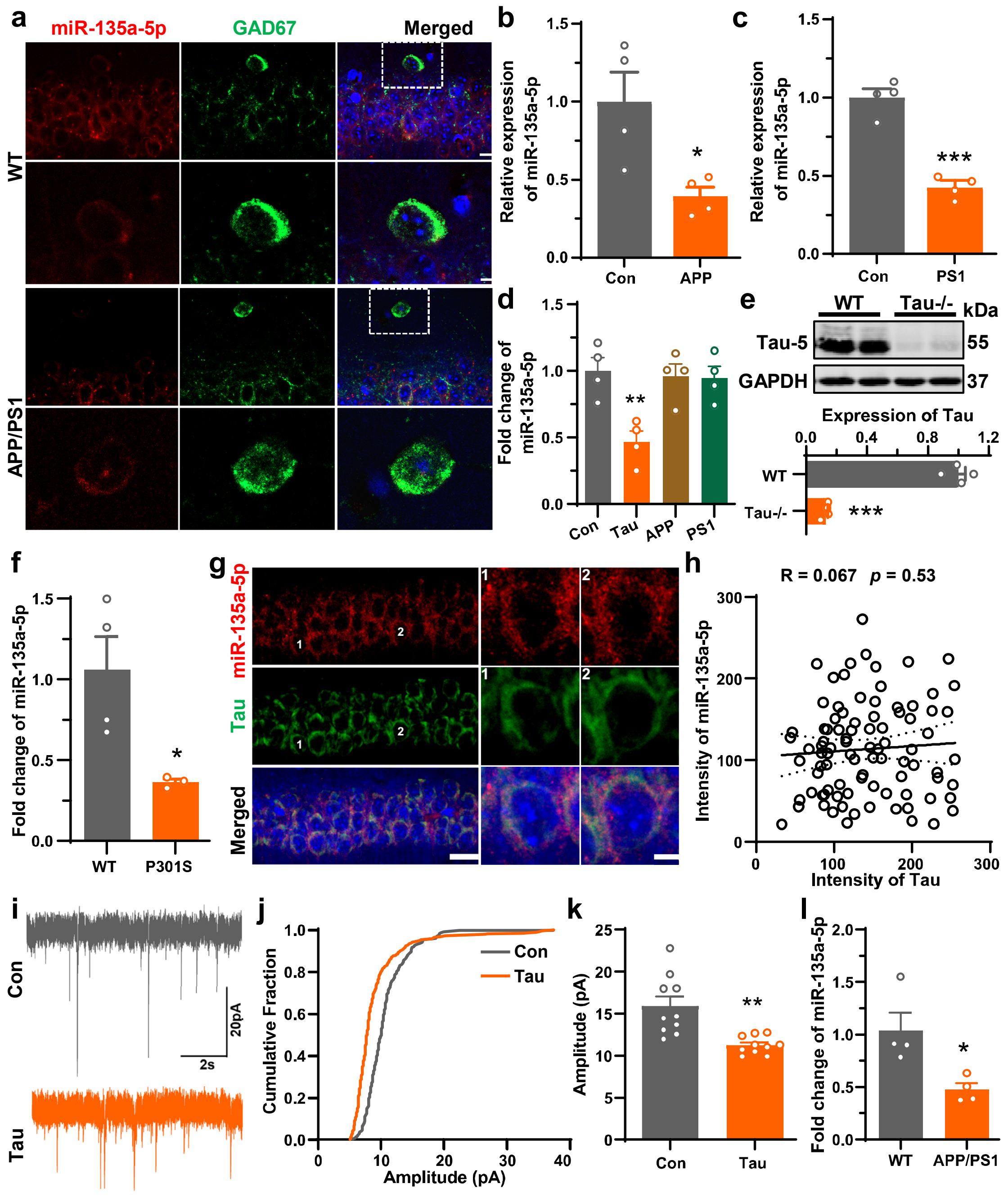


**Supplementary Figure 1. Alteration of miR-135a-5p in the hippocampus of AD mice**

**(a)** The representative immunofluorescence image of miR-135a-5p (Red) and GAD67 (Green) in hippocampus of APP/PS1 and wild type (WT) mice. Scale bar = 20μm

(upper), 5μm (lower).

**(b-c)** The expression of miR-135a-5p in the primary culture neurons after infection with APP (**b**), PS1 (**c**) or control virus (n = 4 for each group).

**(d)** The primary hippocampal neurons from Tau knockout mice at DIV 7 were infected with hTau, APP, PS1 and control virus. Then the expression of miR-135a-5p was detected by qRT-PCR (n = 4 for each group, one-way ANOVA with Dunnett's post hoc, $p = 0.0035$).

**(e)** The hippocampal lyses of wild type (WT) and Tau knockout (Tau-/-) mice were immunoblotted with anti-Tau-5 (representative blots in the upper panel) and then quantitative analysis was performed (lower panel) (n = 4 for each group, $p < 0.0001$).

**(f)** The expression of miR-135a-5p in hippocampus of 6 months P301S mice and wild type littermates (WT) was detected by qRT-PCR (n = 4, 3 for WT, APP/PS1).

**(g)** A representative immunofluorescence staining of miR-135a-5p (Red) and Tau-5 (Tau, Green) in hippocampus of 9 months WT mice. Scale bar = 20μm (left), 5μm (right).

**(h)** The correlative analysis for the fluorescence of miR-135a-5p and Tau-5 in WT mice (n = 70 neurons from 5 mice).

**(i-k)** The AAV packed hTau (Tau) or control (Con) virus was injected into hippocampus of 6-month-old WT mice. One month later, the hippocampal slices were used for mEPSCs recording. The representative traces (**i**), normalized cumulative probability distributions of mEPSCs amplitudes (**j**), mean mEPSCs amplitudes (**k**) were analyzed (n = 10 neurons from 4 mice for each group).

**(l)** The expression of miR-135a-5p in frontal cortex from APP/PS1 and WT mice were measured by qRT-PCR (n = 4 for each group).

(Data are presented as mean ± S.E.M. and two-tailed t-tests were used unless otherwise specified. Source data are provided as a Source Data file. **p*<0.05, ***p*<0.01, ****p*<0.001 *vs* WT/Con)

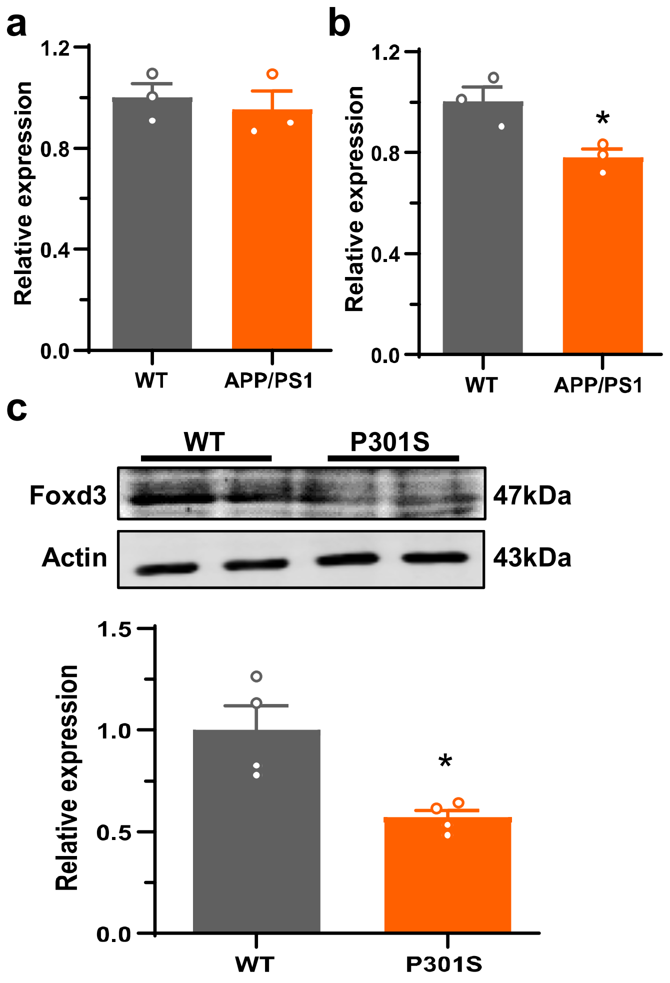


**Supplementary Figure 2. Loss of miR-135a-5p in AD is caused by the reduction of Foxd3**

**(a-b)** The expression of pri-miR-135a-1 in hippocampus of 3 months (**a**) and 6 months (**b**) APP/PS1 and age-matched WT mice (n = 3 for each group).

**(c)** The protein level of Foxd3 in hippocampus of 6 months P301S mice was analyzed by western blotting (n = 4 for each group).

(Data are presented as mean ± S.E.M. and two-tailed t-tests were used. Source data are provided as a Source Data file. **p*<0.05 *vs* WT)

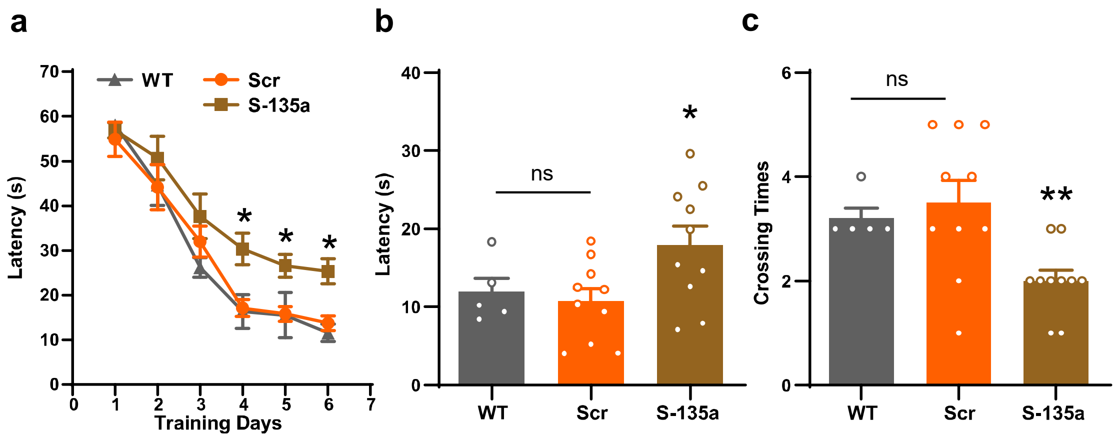


**Supplementary Figure 3. Inhibition of miR-135a-5p induces the memory impairments *in vivo***

The AAV packed miR-135a-5p sponge (S-135a) or scrambled control virus (Scr) were injected into hippocampus of 6-month-old wild type C57 mice. One month later, mice were subjected to the Morris water maze test.

**(a)** The representative traces to hidden platform on day 6 of Morris water maze (n = 5,10,10 for each group, repeated measures two-way ANOVA with Sidak post hoc, *p* = 0.0062).

**(b)** Latencies to the hidden platform in Morris water maze at learning stage were recorded (n = 5,10,10 for each group, one-way ANOVA with Tukeys post hoc, *p* = 0.0375).

**(c)** The representative traces on the probe trial at day 8 (n = 5,10,10 for each group,

one-way ANOVA with Tukeys post hoc , $p = 0.0072$).

(Data are presented as mean ± S.E.M. Source data are provided as a Source Data file. $*p < 0.05$, $**p < 0.01$ *vs* WT)

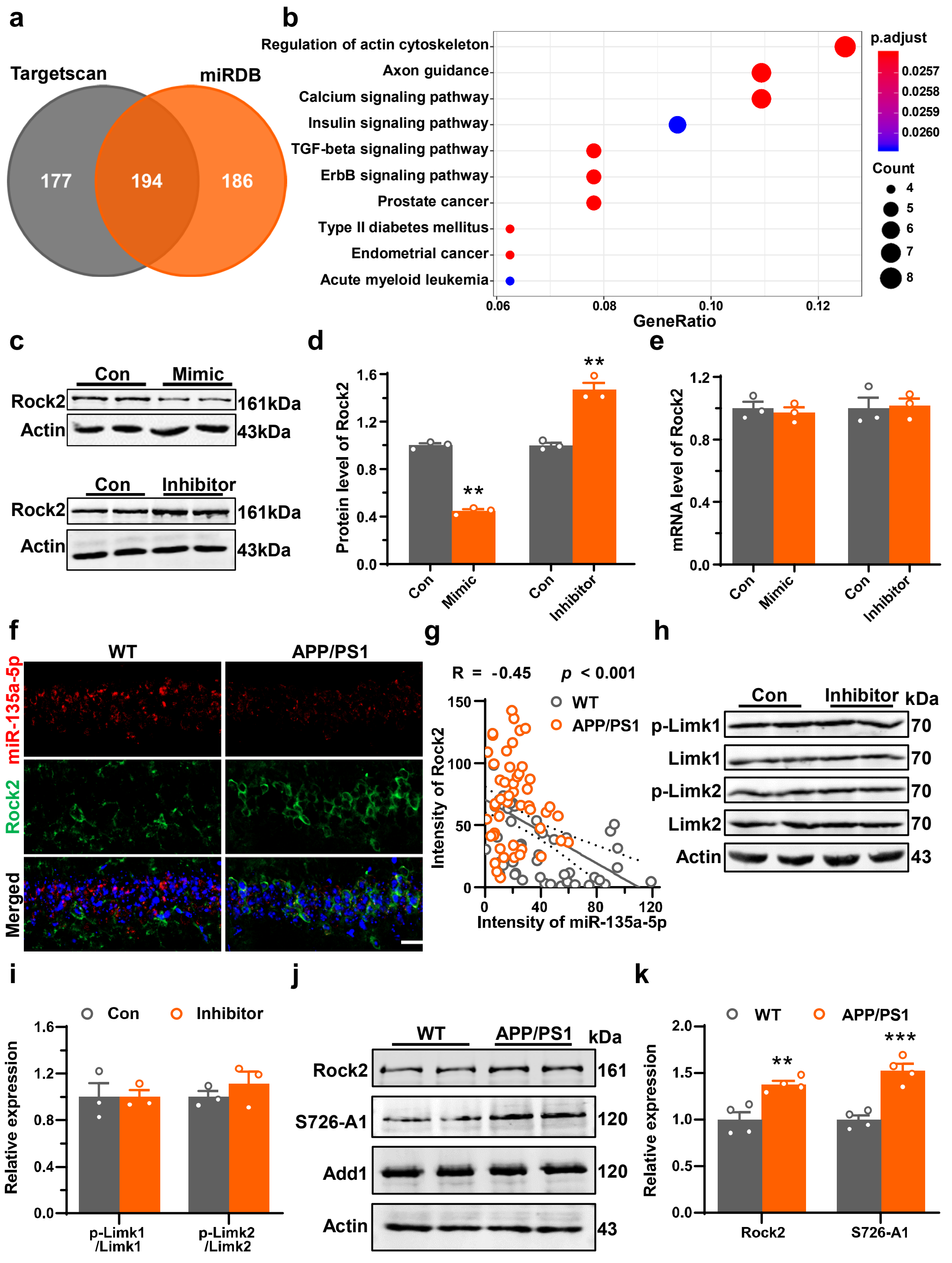


**Supplementary Figure 4. Loss of miR-135a-5p results in the activation of Rock2**

**signal pathway**

**(a)** Venn diagram shows the potential target genes of miR-135a-5p predicted by Targetscan (Gray) and miRDB (Orange).

**(b)** The enrichment plot of predicted target genes of miR-135a-5p after KEGG enrichment analysis with clusterProfiler.

**(c-e)** N2a cells were transfected with miR-135a-5p mimic (Mimic) or inhibitor (Inhibitor) or the corresponded scrambled control (Con). Western blotting was used to detect and quantify the protein level of Rock2 (**c-d**) and qRT-PCR was used to detect the mRNA level of Rock2 (**e**) at 48h later (n = 3 for each group).

**(f)** A representative immunofluorescence staining of miR-135a-5p and Rock2 in hippocampus of 9 months APP/PS1 and C57 mice. Scale bar = 20μm (left), 5μm (right).

**(g)** The correlative analysis for the fluorescence intensities of miR-135a-5p and Rock2 were evaluated in **f** (n = 98 neurons from 6 mice for each group).

**(h-i)** The primary hippocampal neurons at DIV 14 were transfected with miR-135a-5p inhibitor or scrambled control for 48h. Western blotting was used to analyze the protein and phosphorylation levels of Limk1 (p-Limk1 for T508) and Limk2 (p-Limk2 for T505) (n = 3 for each group).

**(j-k)** The expression of Rock2 and S726-Add1 (S726-A1) in frontal cortex homogenates from 9 months APP/PS1 and WT mice were measured by western blotting (**j**). The quantitative analysis was in **k** (n = 4 for each group).

(Data are presented as mean ± S.E.M. and two-tailed t-tests were used. Source data are

provided as a Source Data file. **$p$<0.01, ***$p$<0.001 *vs* WT/Con)

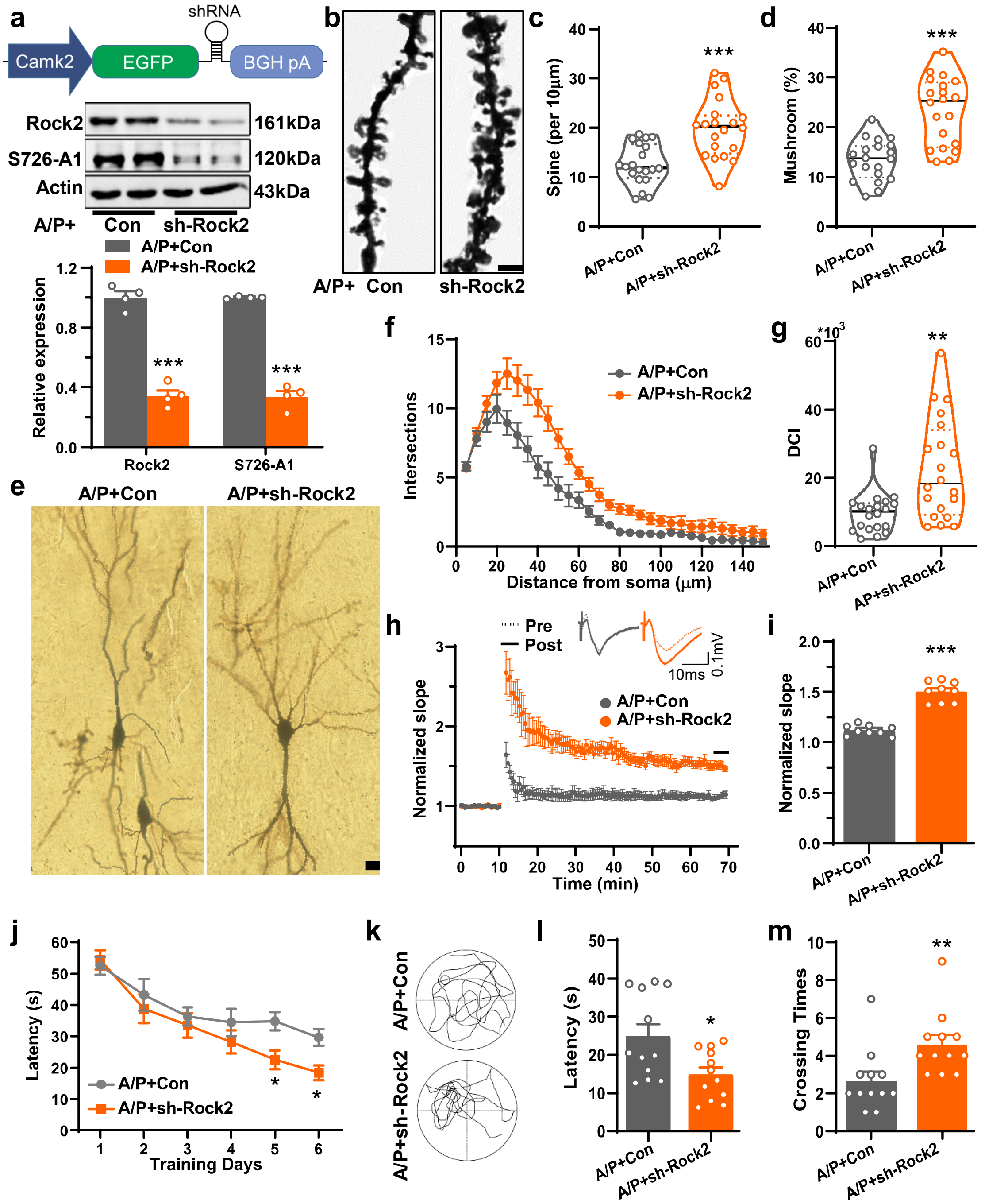


**Supplementary Figure 5. Silencing of Rock2 rescues the memory impairments and synaptic disorder in AD mice**

The sh-Rock2 (A/P+sh-Rock2) or control virus (A/P+Con) was injected into hippocampus of APP/PS1 mice; After 4 weeks, the mice were subjected to Morris

water maze, electrophysiological recordings, western blotting and Golgi staining.

**(a)** Schematic illustration (top) of the shRNA design and western blotting analyzed (down) hippocampal homogenates with Rock2, S726-Add1 antibody (n = 4 for each group, $p < 0.0001$).

**(b-d)** Golgi staining was performed to show spines distribution of mice. (**b**) Representative images of dendritic spine. The quantitative analysis of the spine density (per 10 μm) (**c**) and percentage of mushroom spines (**d**) was performed (n = 20 neurons from 6 mice for each group, $p < 0.0001$).

**(e)** Golgi staining showed the morphology of neurons in the CA1 region. Bar = 20μm.

**(f-g)** Sholl analysis (**f**) and Dendritic Complexity Index (DCI) analysis (**g**) were completed to evaluate the dendritic complexity of hippocampal neurons (n = 20 neurons from 6 mice for each group).

**(h)** The electrophysiological recording was done in CA3-CA1 projection; Representative traces showed in upper panel.

**(i)** The quantitative analysis was calculated from the last 5min recording in **h** (n = 9 slices from 6 mice for each group).

**(j)** The latencies of mice in Morris water maze from day 1 to 6 were recorded (n = 12 for each group, repeated measures two-way ANOVA with Tukeys post hoc , $p$ = 0.0394).

**(k-m)** The representative traces (**k**), latencies (**l**) and crossing times (**m**) at day 8 were analyzed and showed (n = 12 for each group).

(Data are presented as mean ± S.E.M. and two-tailed t-tests were used unless

otherwise specified. Source data are provided as a Source Data file. *$p<0.05$, **$p<0.01$, ***$p<0.001$ *vs* A/P+Con)

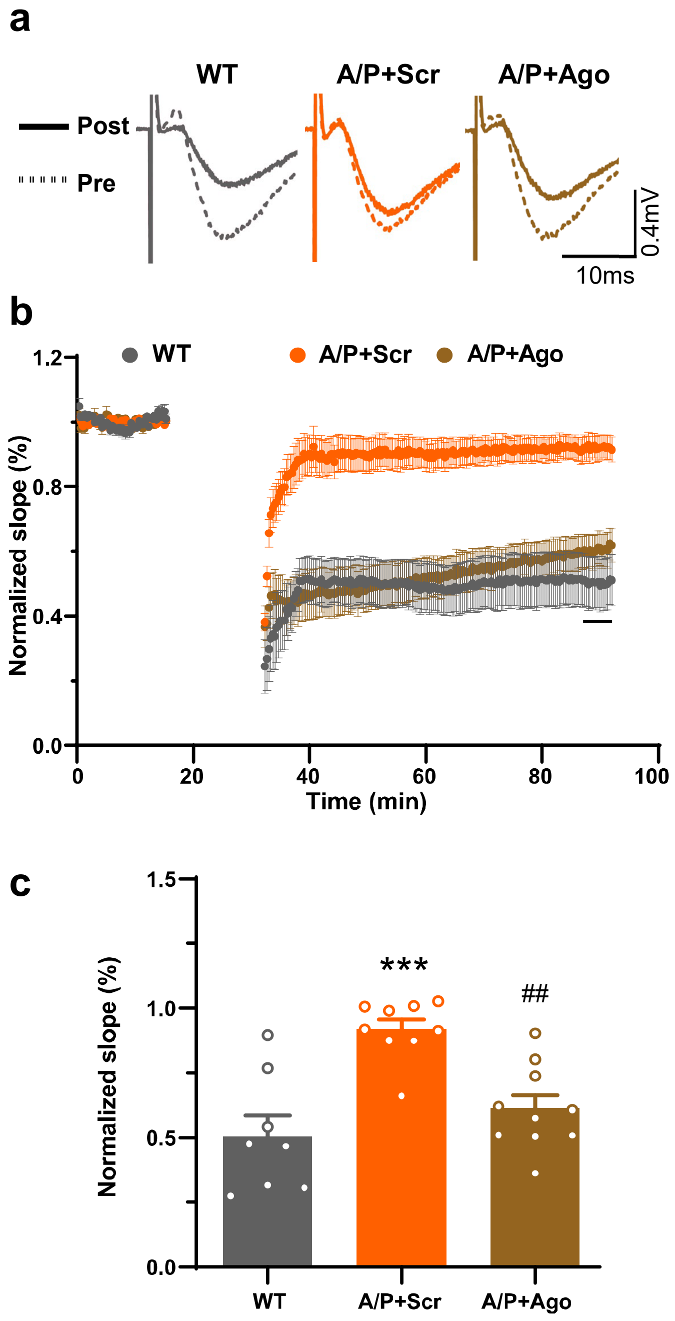


**Supplementary Figure 6. Overexpression of miR-135a-5p rescues the long-term depression in AD mice**

The long-term depression (LTD) electrophysiological recording was performed in

CA3-CA1 projection of APP/PS1 mice or wild type littermates injected with miR-135a-5p agomir (Ago) or scramble (Scr). WT: wild type mice; A/P+Scr: APP/PS1 mice treated with scramble; A/P+Ago: APP/PS1 mice treated with miR-135a-5p agomir.

**(a)** Representative traces were shown before (dash line, pre) and after (solid line, post) low frequency stimulation (LFS).

**(b)** The normalized fEPSP slope at hippocampal CA3-CA1 projection in S-135a or Scr injected mice at before and after LFS (n = 8-10 slices from 5 mice for each group).

**(c)** Quantitative analysis of fEPSP slope at last 5 min as indicated by a black line in panel **b** (n = 8, 9, 10 slices from 5 mice for WT, A/P+Scr, A/P+Ago, one-way ANOVA with Tukeys post hoc , $p < 0.0001$).

(Data are presented as mean ± S.E.M. Source data are provided as a Source Data file. ***$p$<0.001 A/P+Scr *vs* WT, ##$p$<0.01 A/P+Ago *vs* A/P+Scr)

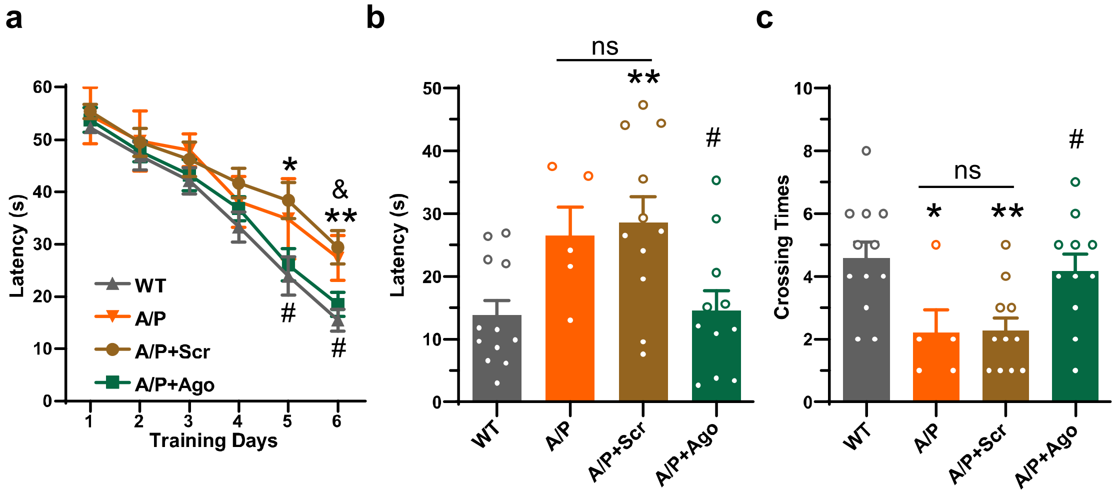


**Supplementary Figure 7. Overexpression of miR-135a-5p rescues the memory**

**impairments and synaptic disorder in AD mice**

The miR-135a-5p agomir (Ago) or scramble (Scr) was injected to CA1 region of APP/PS1 mice or wild type littermates at 8 month-old. 4 weeks later, the mice were subjected to Morris water maze.

**(a)** The representative traces to hidden platform on day 6 of Morris water maze (n = 12,5,11,11 for each group, repeated measures two-way ANOVA with Tukeys post hoc , $p = 0.0007$).

**(b)** Latencies to the hidden platform in Morris water maze at learning stage were recorded (n = 12,5,11,11 for each group, one-way ANOVA with Tukeys post hoc , $p = 0.0045$).

**(c)** The representative traces on the probe trial at day 8 (n = 12,5,11,11 for each group, one-way ANOVA with Tukeys post hoc , $p = 0.0033$).

(Data are presented as mean ± S.E.M. Source data are provided as a Source Data file. &$p < 0.05$ A/P *vs* WT in Day 6)

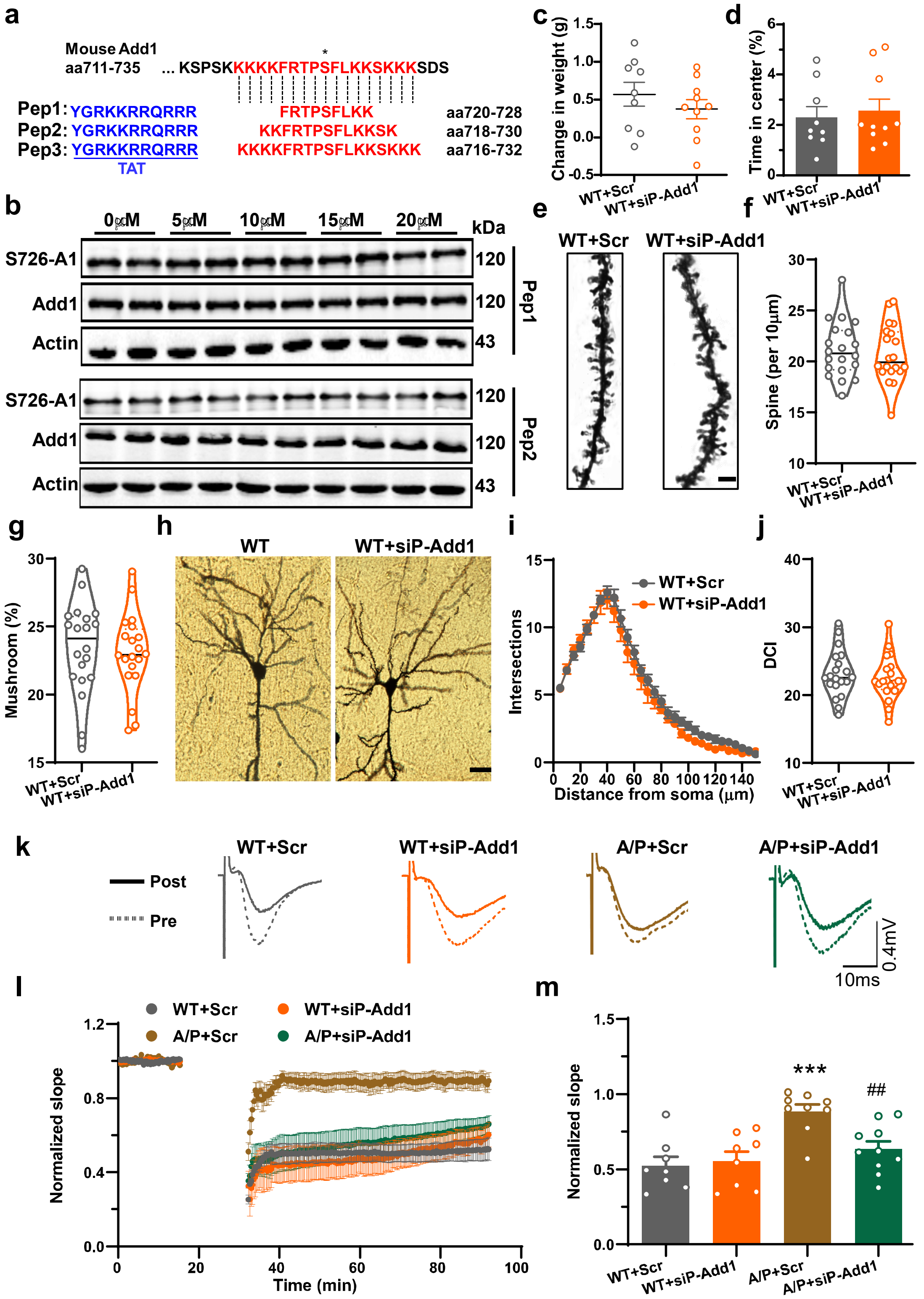


**Supplementary Figure 8. siP-Add1 recovers the long-term depression in AD mice**

**(a)** Schematic illustration of the peptides design and the phosphorylation site was indicated by an asterisk.

**(b)** HEK293T cells were cotransfected with Add1, Rock2 and then treated with peptide 1 (Pep1) and peptide 2 (Pep2). Western blotting was used to analyze level of Add1 and S726-Add1 (n = 4 for each group).

**(c-j)** Wild type (WT) mice were intraperitoneally injected with siP-Add1 or scrambled peptide for two weeks (15mg/kg per day) at 9 months (n = 9, 10 for WT+Scr, WT+siP-Add1). Then mice were subjected to Open field test and Golgi staining.

**(c)** The change of body weight between WT mice intraperitoneally injected with siP-Add1 (WT+siP-Add1) or scrambled peptide (WT+Scr) (n = 9, 10 for WT+Scr, WT+siP-Add1).

**(d)** Percentage of time spent in centre area of two group mice in Open field test (n = 9, 10 for WT+Scr, WT+siP-Add1).

**(e-g)** Golgi staining was used to evaluate the dendritic spines. The representative images for the dendritic spines in CA1 region (**e**) and quantitative analysis of the spine density (per 10 μm) (**f**) and percentage of mushroom spine (**g**) (n = 20 neurons from 4 mice for each group).

**(h-j)** Golgi staining was used to analyze the dendritic trees in the hippocampal CA1 neurons. (**h**) The representative images. Bar = 20μm. The Sholl analysis (**i**) and Dendritic Complexity Index (DCI) analysis (**j**) were performed to evaluate the dendritic complexity (n = 20 neurons from 4 mice for each group).

**(k-m)** The long-term depression (LTD) electrophysiological recording was performed in CA3-CA1 projection of APP/PS1 mice or wild type littermates intraperitoneally injected with siP-Add1 or scrambled peptide (Scr).

**(k)** Representative traces were shown before (dash line, pre) and after (solid line, post) LFS.

**(l)** The normalized fEPSP slope at hippocampal CA3-CA1 projection in four group mice at before and after LFS (n = 8, 8, 9, 10 slices from 5 mice for WT+Scr, WT+siP-Add1, A/P+Scr, A/P+siP-Add1).

**(m)** Quantitative analysis of fEPSP slope at last 5 min as indicated by a black line in panel **l** (n = 8, 8, 9, 10 slices from 5 mice for WT+Scr, WT+siP-Add1, A/P+Scr, A/P+siP-Add1, one-way ANOVA with Tukeys post hoc , $p = 0.0002$).

(Data are presented as mean ± S.E.M. and two-tailed t-tests were used unless otherwise specified. Source data are provided as a Source Data file. ***$p<0.001$ A/P+Scr *vs* WT+Scr, ##$p<0.01$ A/P+siP-Add1 *vs* A/P+Scr)
