# Supplementary material for: miR-135a-5p mediates memory and synaptic impairments via the Rock2/Adducin1 signaling pathway in a mouse model of Alzheimer’s disease
Source: Nat Commun. 2021 Mar 26;12:1903. doi: 10.1038/s41467-021-22196-y (PMC7998005; doi:10.1038/s41467-021-22196-y)
Supplement: Supplementary file 2 — Description of Additional Supplementary Files [file 41467_2021_22196_MOESM2_ESM.docx]

**Description of Additional Supplementary Files**

File Name: Supplementary Data 1.

Description: The predicted transcription factor binding sites in pri-miR-135a-1 promoter by RegRNA2.0.

File Name: Supplementary Data 2.

Description: The predicted transcription factor binding sites in pri-miR-135a-1 promoter by PROMO.

File Name: Supplementary Data 3.

Description: The predicted transcription factor binding sites in pri-miR-135a-1 promoter by LASAGNA.

File Name: Supplementary Data 4.

Description: The predicted the potential targets of miR-135a-5p by Targetscan.

File Name: Supplementary Data 5.

Description: The predicted the potential targets of miR-135a-5p by miRDB.
