# Supplementary material for: miR-135a-5p mediates memory and synaptic impairments via the Rock2/Adducin1 signaling pathway in a mouse model of Alzheimer’s disease
Source: Nat Commun. 2021 Mar 26;12:1903. doi: 10.1038/s41467-021-22196-y (PMC7998005; doi:10.1038/s41467-021-22196-y)
Supplement: Supplementary file 3 — Supplementary Data [file 41467_2021_22196_MOESM3_ESM.docx]

**Supplementary Data 1. The predicted transcription factor binding sites in pri-miR-135a-1 promoter by RegRNA2.0.**

| Motif Name | Position | Length | Sequence |
| --- | --- | --- | --- |
| Ik-1 | 1635 ~ 1647 | 13 | caatgggaatacg |
| Ik-2 | 1635 ~ 1646 | 12 | caatgggaatac |
| Ik-3 | 1635 ~ 1647 | 13 | caatgggaatacg |
| c-Myc:Max | 1552 ~ 1565 | 14 | tgctcacgtgttta |
| Max | 1552 ~ 1565 | 14 | tgctcacgtgttta |
| USF | 1552 ~ 1565 | 14 | tgctcacgtgttta |
| GATA-1 | 1287 ~ 1299 | 13 | accagataaatag |
| FOXD3 | 1260 ~ 1271 | 12 | atttgtttattt |
| Pax-5 | 1898 ~ 1925 | 28 | ccagagcccagtggagatgggaggccct |
| SRY | 297 ~ 308 | 12 | gaaaacaatggc |
| AhR:Arnt | 1019 ~ 1034 | 16 | tattaaaagcgtgcac |
| Olf-1 | 1712 ~ 1733 | 22 | acaaaatccccagagtctctga |
| HFH8_(FOXF1A) | 1260 ~ 1272 | 13 | atttgtttatttc |
| Pax-3 | 1959 ~ 1979 | 21 | tcacaccctcacgatggaaga |
| SOX9 | 296 ~ 309 | 14 | ggaaaacaatggca |
| TTF1_(Nkx2-1) | 980 ~ 989 | 10 | actcaagaga |
| POU3F2 | 737 ~ 750 | 14 | agccattcattcat |
| FOXO3 | 1259 ~ 1272 | 14 | aatttgtttatttc |
| Arnt | 1549 ~ 1568 | 20 | cactgctcacgtgtttacct |
| c-Myc:Max | 1549 ~ 1568 | 20 | cactgctcacgtgtttacct |
| LXRalpha:RXRalpha | 1650 ~ 1664 | 15 | cccaaggtcaagaac |
| TEF-1 | 50 ~ 55 | 6 | ggaatg |
| myogenin | 670 ~ 677 | 8 | ggcagctg |
| USF2 | 1556 ~ 1561 | 6 | cacgtg |
| Osf2 | 206 ~ 213 | 8 | accacaaa |
| SMAD | 1574 ~ 1582 | 9 | agacaccaa |
| TTF-1_(Nkx2-1) | 979 ~ 990 | 12 | cactcaagagat |
| Pit-1 | 740 ~ 757 | 18 | cattcattcatttattta |
| FOX_factors | 1260 ~ 1272 | 13 | atttgtttatttc |
| GR | 884 ~ 910 | 27 | tgctgctgctgctgctgttcttggtga |
| SMAD | 1404 ~ 1414 | 11 | aaggcagacac |
| Pax-6 | 939 ~ 952 | 14 | atgtcctggaactc |
| TBX5 | 119 ~ 128 | 10 | tcaggtgtgt |
| AP-2alphaA | 327 ~ 341 | 15 | ttcgccttgggctgg |
| Kid3 | 149 ~ 153 | 5 | ccacc |
| Kid3 | 266 ~ 270 | 5 | ccacc |
| Kid3 | 1034 ~ 1038 | 5 | ccacc |
| Kid3 | 1483 ~ 1487 | 5 | ccacg |
| FOXO1 | 1218 ~ 1226 | 9 | aaaaacaaa |
| MAFB | 496 ~ 501 | 6 | gctgac |
| GTF2IRD1-isoform2 | 1162 ~ 1170 | 9 | gggattata |
| IPF1 | 709 ~ 718 | 10 | ccataattag |
| Nanog | 1214 ~ 1233 | 20 | agaaaaaaacaaaaaaaaca |
| NFAT1 | 296 ~ 301 | 6 | ggaaaa |
| Neuro_D | 672 ~ 677 | 6 | cagctg |
| Pax-4 | 707 ~ 723 | 17 | atccataattagtaaga |
| dlx5 | 708 ~ 723 | 16 | tccataattagtaaga |
| STAT3 | 769 ~ 784 | 16 | agtgctgggaactcaa |
| MAFA | 1457 ~ 1463 | 7 | tcagcag |
| RORBETA | 1945 ~ 1951 | 7 | tgaccta |
| AML2 | 205 ~ 212 | 8 | gaccacaa |
| GKLF | 1825 ~ 1831 | 7 | cctcctt |
| Dec2 | 1553 ~ 1562 | 10 | gctcacgtgt |
| IRF-4 | 606 ~ 612 | 7 | gaaagta |
| NF-AT4 | 296 ~ 301 | 6 | ggaaaa |
| Smad3 | 1405 ~ 1417 | 13 | aggcagacacggg |
| ERR3 | 1652 ~ 1659 | 8 | caaggtca |
| GATA-5 | 351 ~ 356 | 6 | tatctg |
| GCNF | 1652 ~ 1661 | 10 | caaggtcaag |
| HNF-3beta | 1331 ~ 1339 | 9 | ctgtttgct |
| MEF-2C | 752 ~ 758 | 7 | tatttat |
| MEF-2C | 761 ~ 767 | 7 | tattttt |
| MEF-2D | 1293 ~ 1299 | 7 | taaatag |
| Cdx-1 | 750 ~ 755 | 6 | tttatt |
| Cdx-1 | 754 ~ 759 | 6 | tttatt |
| Cdx-1 | 759 ~ 764 | 6 | tttatt |
| Cdx-1 | 1265 ~ 1270 | 6 | tttatt |
| ERR1 | 1652 ~ 1662 | 11 | caaggtcaaga |
| LHX3 | 1020 ~ 1025 | 6 | attaaa |
| myogenin | 672 ~ 677 | 6 | cagctg |
| OC-2 | 1132 ~ 1137 | 6 | tcaata |
| SOX10 | 208 ~ 214 | 7 | cacaaag |
| SOX10 | 300 ~ 306 | 7 | aacaatg |
| Sox30 | 296 ~ 311 | 16 | ggaaaacaatggcaca |
| Zic1 | 356 ~ 369 | 14 | gagccccagggggc |
| Arid5a | 1637 ~ 1653 | 17 | atgggaatacgttccca |
| Foxl1 | 1214 ~ 1229 | 16 | agaaaaaaacaaaaaa |
| Rxra | 1309 ~ 1324 | 16 | tatagaaggttatgcc |
| Sox14 | 296 ~ 310 | 15 | ggaaaacaatggcac |
| Zic3 | 1453 ~ 1467 | 15 | gccctcagcaggcaa |

**Supplementary Data 2. The predicted transcription factor binding sites in pri-miR-135a-1 promoter by PROMO.**

| Factor name | Start | End | Dissimilarity | String | RE equally | RE query |
| --- | --- | --- | --- | --- | --- | --- |
| abaA [T01085] | 49 | 58 | 1.113237 | GGAATGGAGC | 0.0248 | 0.02399 |
| ABF1 [T00056] | 383 | 388 | 4.757663 | ATCAAA | 0.97656 | 0.97881 |
| ABI4 [T05743] | 1505 | 1510 | 0.083884 | AGCACC | 0.97656 | 0.99885 |
| Adf-1 [T00008] | 890 | 897 | 2.706324 | GCTGCTGC | 0.06104 | 0.05998 |
| ADR1 [T00011] | 1697 | 1704 | 2.458834 | ACCCCAGC | 0.12207 | 0.13262 |
| AGL3 [T03025] | 1757 | 1762 | 1.226163 | AGAAAT | 0.97656 | 0.98502 |
| AhR [T00018] | 1023 | 1031 | 8.477296 | AAAGCGTGC | 0.2594 | 0.26408 |
| AhR [T01795] | 1026 | 1033 | 2.665859 | GCGTGCAC | 0.45776 | 0.45717 |
| AhR:Arnt [T05394] | 1022 | 1030 | 9.325207 | AAAAGCGTG | 0.20599 | 0.20751 |
| AIRE [T05990] | 30 | 39 | 8.565165 | AGCAAACCAT | 0.11444 | 0.12752 |
| ALF1B [T01496] | 795 | 802 | 14.87844 | ACACATGC | 0.54932 | 0.54618 |
| Alfin1 [T04733] | 1030 | 1036 | 1.550756 | GCACCAC | 0.24414 | 0.2559 |
| aMEF-2 [T01006] | 1262 | 1274 | 13.87633 | TGTTTATTTCTAT | 0.02745 | 0.0284 |
| AML1 [T01067] | 1318 | 1330 | 5.411546 | TATGCCCACAAAC | 0.00358 | 0.00386 |
| AML1a [T02256] | 205 | 214 | 0.69261 | ACCACAAAGA | 0.04005 | 0.04761 |
| ANF [T00025] | 1283 | 1294 | 9.771221 | ATGACCAGATAA | 0.03219 | 0.03161 |
| ANF [T01183] | 1283 | 1294 | 9.771221 | ATGACCAGATAA | 0.03219 | 0.03161 |
| ANF [T01184] | 1283 | 1294 | 9.771221 | ATGACCAGATAA | 0.03219 | 0.03161 |
| ANT [T02639] | 231 | 241 | 11.86245 | TTACAGATCCC | 0.05817 | 0.05909 |
| Antp [T00026] | 1162 | 1167 | 5.347237 | GGATTA | 0.48828 | 0.48213 |
| AP-1 [T00029] | 419 | 426 | 5.881359 | TGACTGAC | 0.12207 | 0.11875 |
| AP-1 [T00031] | 907 | 917 | 14.31903 | TGAGACAGGGG | 0.48065 | 0.47535 |
| AP-1 [T00032] | 83 | 90 | 14.84926 | TGACTGGT | 0.12207 | 0.1233 |
| AP-1 [T01140] | 907 | 913 | 14.28571 | TGAGACA | 2.56348 | 2.51916 |
| AP-2 [T00034] | 1717 | 1726 | 6.521557 | TCCCCAGAGT | 0.01907 | 0.0194 |
| AP-2alpha [T00033] | 1476 | 1484 | 0 | TGGTCCCCA | 0.06104 | 0.06177 |
| AP-2alphaA [T00035] | 1788 | 1793 | 1.437215 | GCCTGT | 0.48828 | 0.4861 |
| AP-2beta [T02469] | 1411 | 1422 | 4.98533 | CACGGGGAGCCT | 0.00966 | 0.00969 |
| AP-3 (2) [T00039] | 250 | 256 | 1.444018 | CTAAGTC | 0.12207 | 0.11875 |
| AP-3 [T01150] | 1397 | 1405 | 14.13275 | GTGACCAAG | 0.03052 | 0.03083 |
| AP3:PI [T03251] | 1078 | 1088 | 14.56513 | TCTTAGTTGCC | 0.14019 | 0.13954 |
| AP-4 [T00036] | 667 | 677 | 3.001548 | ACGGCAGCTGA | 0.01764 | 0.01732 |
| AR [T00040] | 1839 | 1846 | 2.872832 | TAGGAACA | 0.12207 | 0.11876 |
| AR [T00042] | 1694 | 1700 | 3.331665 | AGAACCC | 0.48828 | 0.50883 |
| AREB6 [T00625] | 119 | 126 | 0.330765 | CAGGTGTG | 0.09155 | 0.09355 |
| Arnt [T01346] | 1022 | 1030 | 9.260885 | AAAAGCGTG | 0.18311 | 0.18523 |
| ARP-1 [T00045] | 497 | 505 | 12.8338 | TGACCGTGA | 0.06104 | 0.05974 |
| AT-BP1 [T01015] | 1713 | 1722 | 0 | AAAATCCCCA | 0.01717 | 0.0186 |
| AT-BP2 [T01016] | 1713 | 1722 | 0 | AAAATCCCCA | 0.01717 | 0.0186 |
| ATF3 [T01095] | 423 | 430 | 6.137987 | TGACTTGC | 0.24414 | 0.24254 |
| ATHB-5 [T04066] | 702 | 712 | 11.76155 | AATAATCCATA | 0.04196 | 0.04138 |
| Bcd [T00063] | 1159 | 1167 | 0.934775 | CTGGGATTA | 0.04578 | 0.04773 |
| BR-C Z1 [T01477] | 1261 | 1272 | 6.307381 | TTGTTTATTTCT | 0.00954 | 0.01001 |
| BR-C Z2 [T01478] | 1760 | 1764 | 3.410996 | AATGG | 1.95312 | 1.91622 |
| BR-C Z3 [T01479] | 1217 | 1226 | 2.764555 | AAAAACAAAA | 0.01717 | 0.01914 |
| BR-C Z4 [T01480] | 753 | 765 | 13.02943 | TTTATTTTATTTT | 0.01127 | 0.01289 |
| BTEB3 [T05051] | 1773 | 1781 | 1.158757 | ACTCCACTT | 0.04578 | 0.04664 |
| BTEB4 [T05053] | 1991 | 1999 | 0 | GGCCGCCCC | 0.04578 | 0.05072 |
| C/EBP [T01386] | 1864 | 1869 | 1.583727 | TGCAAG | 0.97656 | 0.95817 |
| C/EBPalpha [T00104] | 853 | 858 | 0 | TGTTGC | 0.48828 | 0.50883 |
| C/EBPalpha [T00105] | 1486 | 1491 | 0 | GAGCAA | 0.48828 | 0.48417 |
| C/EBPalpha [T00108] | 1863 | 1868 | 0.527529 | CTGCAA | 0.97656 | 0.95817 |
| C/EBPbeta [T00017] | 582 | 589 | 7.42946 | TGTGGCAA | 0.18311 | 0.17787 |
| C/EBPbeta [T00459] | 1486 | 1491 | 0.386721 | GAGCAA | 0.97656 | 0.97027 |
| C/EBPbeta [T00581] | 427 | 433 | 1.681833 | TTGCTCT | 0.24414 | 0.24209 |
| C/EBPbeta(p20) [T01420] | 1762 | 1769 | 10.50019 | TGGTACAA | 1.19019 | 1.17388 |
| C/EBPgamma [T00216] | 582 | 589 | 3.747937 | TGTGGCAA | 0.24414 | 0.23798 |
| C/EBPgamma [T02028] | 580 | 589 | 13.87528 | ATTGTGGCAA | 0.03052 | 0.02988 |
| C1 (long form) [T01592] | 1662 | 1669 | 12.5 | ACACGGGG | 0.73242 | 0.72324 |
| C1 (short form) [T01593] | 1662 | 1669 | 12.5 | ACACGGGG | 0.73242 | 0.72324 |
| C1-I [T02946] | 1662 | 1669 | 12.5 | ACACGGGG | 0.73242 | 0.72324 |
| CAC-binding protein [T00076] | 1030 | 1037 | 2.152461 | GCACCACC | 0.18311 | 0.19617 |
| Cart-1 [T03978] | 712 | 716 | 0 | AATTA | 1.95312 | 1.90095 |
| CAT8 [T03227] | 474 | 485 | 13.51903 | AGGATAAATGGG | 0.23174 | 0.23186 |
| CBF1 [T00080] | 1553 | 1560 | 4.898644 | CTCACGTG | 0.06104 | 0.05974 |
| CD28RC [T00102] | 1254 | 1266 | 13.21602 | GAGGAATTTGTTT | 0.02897 | 0.02982 |
| Cdx-1 [T01484] | 1837 | 1840 | 2.545394 | CTTA | 7.8125 | 7.64982 |
| CDX2 [T03246] | 758 | 769 | 13.89357 | TTTATTTTTCAG | 0.04148 | 0.04201 |
| c-Ets-1 [T00111] | 1777 | 1786 | 10.94606 | CACTTCCCTA | 0.14496 | 0.14368 |
| c-Ets-1 [T00112] | 1637 | 1643 | 11.27844 | TGGGAAT | 0.12207 | 0.12279 |
| c-Ets-2 [T00113] | 1255 | 1262 | 2.827022 | AGGAATTT | 0.36621 | 0.35628 |
| c-Ets-2 [T01397] | 1756 | 1764 | 4.495539 | AAGAAATGG | 0.03052 | 0.02938 |
| CF2-II [T00120] | 727 | 736 | 5.692764 | TGTGCATATA | 0.02098 | 0.02015 |
| c-Fos [T00122] | 83 | 90 | 4.258138 | TGACTGGT | 0.18311 | 0.18219 |
| c-Fos [T00123] | 1534 | 1541 | 7.473109 | CTGACACC | 0.12207 | 0.12026 |
| c-Fos [T00124] | 83 | 89 | 8.666606 | TGACTGG | 0.36621 | 0.36225 |
| Chx10 [T04139] | 711 | 721 | 2.890583 | TAATTAGTAAG | 0.04005 | 0.03897 |
| CIZ6-1 [T05137] | 759 | 768 | 8.334427 | TTATTTTTCA | 0.06485 | 0.06838 |
| c-Jun [T00131] | 1470 | 1475 | 4.581115 | TGACAG | 0.97656 | 0.95389 |
| c-Jun [T00132] | 1944 | 1950 | 2.639894 | TGACCTA | 0.24414 | 0.23847 |
| c-Jun [T00133] | 1470 | 1476 | 9.796968 | TGACAGT | 0.24414 | 0.23751 |
| c-Myb [T00137] | 316 | 323 | 14.45932 | TAACTCTG | 0.36621 | 0.36757 |
| c-Myb [T00138] | 1324 | 1332 | 3.792511 | CACAAACTG | 0.11444 | 0.11259 |
| c-Myc [T00140] | 1555 | 1560 | 0 | CACGTG | 0.48828 | 0.47791 |
| c-Myc [T00142] | 1552 | 1563 | 9.257019 | GCTCACGTGTTT | 0.07725 | 0.07572 |
| c-Myc [T00143] | 1007 | 1013 | 6.683183 | CCAAGTG | 0.24414 | 0.23847 |
| COE1 [T01112] | 408 | 414 | 0.459822 | TCCCTGG | 0.12207 | 0.11972 |
| COE2 [T05006] | 434 | 442 | 2.349284 | ACAGTCCCT | 0.06104 | 0.0633 |
| COE3 [T05008] | 1713 | 1725 | 6.810722 | AAAATCCCCAGAG | 0.01341 | 0.0132 |
| COUP-TF1 [T00149] | 1650 | 1658 | 0.307042 | CCAAGGTCA | 0.11444 | 0.11152 |
| CP2 [T00152] | 924 | 931 | 0 | CTGGGTAG | 0.03052 | 0.03165 |
| CPRF-2 [T01092] | 1553 | 1561 | 3.662545 | CTCACGTGT | 0.04578 | 0.04503 |
| CPRF-3 [T01093] | 1553 | 1562 | 14.22349 | CTCACGTGTT | 0.09537 | 0.09327 |
| c-Rel [T01154] | 324 | 333 | 5.420538 | GCTTCGCCTT | 0.07057 | 0.07051 |
| CREMtau [T01309] | 26 | 32 | 11.08965 | ACACAGC | 0.24414 | 0.2549 |
| CREMtau1 [T02108] | 26 | 32 | 11.08965 | ACACAGC | 0.24414 | 0.2549 |
| CREMtau2 [T02109] | 26 | 32 | 11.08965 | ACACAGC | 0.24414 | 0.2549 |
| CRF [T00170] | 1349 | 1360 | 12.35604 | CCAATTCAGACC | 0.06437 | 0.06912 |
| Croc [T02291] | 751 | 760 | 0 | TATTTATTTT | 0.00572 | 0.00592 |
| Crx [T03461] | 1340 | 1345 | 2.308066 | TAATGG | 0.97656 | 0.95428 |
| CUTL1 [T00100] | 1203 | 1208 | 0.630875 | ATTGAT | 0.48828 | 0.48022 |
| Cutl1 [T02042] | 1632 | 1637 | 1.543329 | TCCAAT | 0.97656 | 0.99082 |
| DBP [T00183] | 369 | 375 | 2.113317 | AGCAGAG | 0.12207 | 0.11925 |
| DBP [T04875] | 1160 | 1169 | 2.898434 | TGGGATTATA | 0.0515 | 0.05439 |
| DEAF-1 [T05885] | 1038 | 1046 | 4.831696 | ACTGCCCGA | 0.09155 | 0.09605 |
| DEC2 [T05845] | 1603 | 1612 | 9.210675 | CTCCTGAAGC | 0.2861 | 0.27935 |
| DEF:GLO [T03216] | 293 | 300 | 3.646766 | ATGGAAAA | 0.12207 | 0.11867 |
| DEF:GLO:SQUA [T03217] | 627 | 633 | 4.026689 | AAAGTAA | 0.48828 | 0.47527 |
| Dl [T00196] | 759 | 767 | 8.681123 | TTATTTTTC | 0.06866 | 0.06904 |
| DPBF-1 [T04363] | 1323 | 1332 | 14.85988 | CCACAAACTG | 0.06866 | 0.07149 |
| DPBF-2 [T04364] | 1323 | 1332 | 14.85988 | CCACAAACTG | 0.06866 | 0.07149 |
| DRF1.1 [T05835] | 1988 | 1997 | 10.80214 | AGGGGCCGCC | 0.08774 | 0.09009 |
| DRF1.3 [T05837] | 1988 | 1997 | 10.80214 | AGGGGCCGCC | 0.08774 | 0.09009 |
| dri [T04679] | 1199 | 1209 | 2.131658 | TGATATTGATG | 0.02718 | 0.02675 |
| DSXF [T00955] | 137 | 143 | 4.354651 | TATGTAG | 0.73242 | 0.73402 |
| DSXM [T00956] | 137 | 143 | 4.354651 | TATGTAG | 0.73242 | 0.73402 |
| DTF-1 [T00201] | 288 | 298 | 1.39007 | GCAACATGGAA | 0.01287 | 0.01291 |
| E12 [T01786] | 1806 | 1814 | 1.073741 | GCCAGCTCA | 0.04578 | 0.04797 |
| E2 [T00205] | 80 | 85 | 1.805876 | CGGTGA | 1.46484 | 1.44612 |
| E2F-1 [T01542] | 1585 | 1591 | 9.608274 | TGCGGCT | 0.97656 | 0.9659 |
| E2F-5 [T01607] | 678 | 685 | 14.50569 | GAGCCAAA | 0.64087 | 0.65966 |
| E47 [T00207] | 1806 | 1812 | 3.634763 | GCCAGCT | 0.12207 | 0.11972 |
| E47 [T05421] | 118 | 127 | 4.788264 | TCAGGTGTGT | 0.04578 | 0.04572 |
| EBF [T05427] | 357 | 367 | 1.581175 | GCCCCAGGGGG | 0.00381 | 0.00375 |
| EFII [T00239] | 584 | 594 | 13.50434 | TGGCAAAATCC | 0.14877 | 0.15243 |
| EIIaE-A [T00246] | 284 | 290 | 4.354651 | GAGGGCA | 0.73242 | 0.72692 |
| Elf-1 [T01019] | 1218 | 1227 | 6.915783 | AAAACAAAAA | 0.06104 | 0.06874 |
| Elk-1 [T00250] | 1973 | 1977 | 0 | GGAAG | 1.95312 | 1.9239 |
| EmBP-1a [T02669] | 530 | 539 | 14.3813 | GCCAAGTGCA | 0.12016 | 0.11798 |
| EmBP-1a [T04819] | 1856 | 1865 | 14.66699 | GCCAGGTCTG | 0.40054 | 0.3981 |
| EmBP-1b [T01098] | 1554 | 1566 | 7.290915 | TCACGTGTTTACC | 0.00581 | 0.00571 |
| En-1 [T02016] | 1741 | 1746 | 3.789256 | ACAAGG | 0.48828 | 0.48417 |
| ENKTF-1 [T00255] | 1802 | 1809 | 5.687009 | CAGAGCCA | 0.73242 | 0.74096 |
| ER-alpha [T00261] | 1397 | 1404 | 2.304668 | GTGACCAA | 0.18311 | 0.18391 |
| ER-alpha [T00264] | 1944 | 1953 | 6.554287 | TGACCTAGAG | 0.05341 | 0.05169 |
| ER-beta [T04651] | 1944 | 1952 | 1.269573 | TGACCTAGA | 0.03052 | 0.0295 |
| ERR1 [T04849] | 1652 | 1662 | 2.578868 | AAGGTCAAGAA | 0.01287 | 0.01247 |
| ERRalpha1 [T05682] | 1648 | 1660 | 14.49251 | TCCCAAGGTCAAG | 0.03809 | 0.03692 |
| ETF [T00270] | 1360 | 1368 | 7.587476 | CCTGGCCCC | 0.04578 | 0.0474 |
| Eve [T00272] | 1016 | 1024 | 6.673115 | GGTATTAAA | 0.07629 | 0.07372 |
| Evi-1 [T00273] | 1284 | 1296 | 13.8561 | TGACCAGATAAAT | 0.02342 | 0.02281 |
| f(alpha)-f(epsilon) [T00287] | 1585 | 1590 | 4.800249 | TGCGGC | 0.97656 | 0.96594 |
| FOXA4a [T01051] | 750 | 760 | 6.078238 | TTATTTATTTT | 0.01335 | 0.01336 |
| FOXD3 [T02290] | 1228 | 1235 | 2.983153 | AAACAGTC | 0.06104 | 0.06269 |
| FOXD3 [T04166] | 1260 | 1270 | 0.786582 | TTTGTTTATTT | 0.00381 | 0.00482 |
| FOXF1 [T02461] | 1259 | 1271 | 12.60637 | ATTTGTTTATTTC | 0.01878 | 0.02006 |
| FOXI1 [T02474] | 1214 | 1226 | 11.1999 | GAAAAAAACAAAA | 0.01931 | 0.02358 |
| FOXI1a [T02294] | 1260 | 1271 | 4.83871 | TTTGTTTATTTC | 0.01335 | 0.01428 |
| FOXJ1 [T02460] | 1218 | 1230 | 12.05378 | AAAACAAAAAAAA | 0.01073 | 0.01303 |
| FOXJ2 (long isoform) [T04169] | 1262 | 1271 | 0.457647 | TGTTTATTTC | 0.00763 | 0.00843 |
| FOXM1a [T02517] | 1218 | 1229 | 9.32737 | AAAACAAAAAAA | 0.00358 | 0.00513 |
| FOXM1b [T02516] | 1218 | 1229 | 9.32737 | AAAACAAAAAAA | 0.00358 | 0.00513 |
| FOXN2 [T04206] | 193 | 203 | 11.14005 | CTGCTCCTCCA | 0.0515 | 0.05172 |
| FOXO1 [T04203] | 1555 | 1565 | 7.929297 | CACGTGTTTAC | 0.00858 | 0.00854 |
| FOXO3a [T02938] | 488 | 495 | 2.511511 | TACCTAAG | 0.12207 | 0.11851 |
| FOXO4 [T04176] | 1262 | 1271 | 1.225626 | TGTTTATTTC | 0.02289 | 0.02499 |
| FOXP3 [T04280] | 1713 | 1721 | 14.66822 | AAAATCCCC | 0.03052 | 0.034 |
| Fra-1 [T01462] | 419 | 431 | 13.6028 | TGACTGACTTGCT | 0.02238 | 0.02169 |
| FXR [T04494] | 1652 | 1662 | 11.03467 | AAGGTCAAGAA | 0.04673 | 0.04609 |
| FXR:RXR-alpha [T05318] | 1648 | 1659 | 9.33583 | TCCCAAGGTCAA | 0.01931 | 0.01885 |
| GA-BF [T00297] | 1826 | 1832 | 1.537547 | TCCTTCT | 0.73242 | 0.74241 |
| GAGA factor [T00301] | 1148 | 1153 | 0.646054 | CTCTCG | 0.48828 | 0.47791 |
| GAL4 [T00302] | 985 | 992 | 12.77171 | GAGATCCG | 0.73242 | 0.72489 |
| GAMYB [T02679] | 498 | 504 | 9.706667 | GACCGTG | 0.61035 | 0.60173 |
| GATA-1 [T00267] | 1287 | 1295 | 0.561185 | CCAGATAAA | 0.03052 | 0.03039 |
| GATA-1 [T00305] | 1287 | 1294 | 0.283306 | CCAGATAA | 0.24414 | 0.24026 |
| GATA-1 [T00306] | 1688 | 1693 | 1.908093 | GATACC | 2.92969 | 2.8668 |
| GATA-1 [T05705] | 1272 | 1278 | 1.02303 | TATCATC | 0.36621 | 0.35839 |
| GATA-2 [T00308] | 1199 | 1207 | 7.777778 | TGATATTGA | 0.30518 | 0.29968 |
| GATA-2 [T01302] | 1688 | 1695 | 7.288299 | GATACCAG | 0.12207 | 0.11852 |
| GATA-3 [T00311] | 347 | 354 | 3.079083 | TGGTATCT | 0.24414 | 0.24502 |
| GBF [T00315] | 98 | 108 | 10.04531 | AAACCCCTACA | 0.06199 | 0.07127 |
| GCF [T00320] | 1989 | 1999 | 9.92255 | GGGGCCGCCCC | 0.07725 | 0.07983 |
| GCM [T02302] | 798 | 806 | 14.9103 | CATGCTAGA | 0.22888 | 0.22667 |
| GCMa [T02306] | 798 | 806 | 14.9103 | CATGCTAGA | 0.22888 | 0.22667 |
| GCMa [T02307] | 798 | 806 | 14.9103 | CATGCTAGA | 0.22888 | 0.22667 |
| GCMb [T02308] | 798 | 806 | 14.9103 | CATGCTAGA | 0.22888 | 0.22667 |
| GR [T00333] | 1840 | 1846 | 4.03759 | AGGAACA | 0.48828 | 0.48417 |
| GR [T00335] | 894 | 905 | 8.936404 | CTGCTGTTCTTG | 0.02861 | 0.02892 |
| GR [T05076] | 1656 | 1668 | 4.238532 | TCAAGAACACGGG | 0.00262 | 0.0026 |
| GR-alpha [T00337] | 897 | 904 | 0.205677 | CTGTTCTT | 0.06104 | 0.06295 |
| GR-beta [T01920] | 1839 | 1846 | 12.52626 | TAGGAACA | 0.24414 | 0.25288 |
| Gt [T00328] | 700 | 710 | 11.88104 | CTAATAATCCA | 0.12016 | 0.11809 |
| GT-1 [T00339] | 153 | 160 | 5.054824 | ATTCACAC | 0.12207 | 0.12078 |
| GT-1 [T01089] | 1051 | 1061 | 11.28521 | TTTTAAAGGTC | 0.05341 | 0.05145 |
| Hb [T00395] | 1225 | 1231 | 0.245491 | AAAAAAC | 0.36621 | 0.41439 |
| HBP-1a(1) [T01394] | 1644 | 1651 | 8.66752 | ACGTTCCC | 0.06104 | 0.06177 |
| HBP-1a(c14) [T01395] | 1644 | 1651 | 8.66752 | ACGTTCCC | 0.06104 | 0.06177 |
| HELIOS [T06012] | 1929 | 1934 | 2.857143 | AGGACA | 0.97656 | 0.96016 |
| HES-1 [T01649] | 1967 | 1973 | 4.514243 | CACGATG | 0.24414 | 0.23895 |
| HFH-1 [T02288] | 1259 | 1270 | 5.797006 | ATTTGTTTATTT | 0.00262 | 0.00287 |
| Hlf [T01071] | 486 | 494 | 4.893846 | TTTACCTAA | 0.07629 | 0.07405 |
| HMG I(Y) [T02368] | 1256 | 1262 | 4.038828 | GGAATTT | 0.36621 | 0.36078 |
| HNF-1A [T00368] | 175 | 182 | 4.541662 | GTTAAACC | 0.24414 | 0.23655 |
| HNF-1B [T01950] | 711 | 718 | 3.679411 | TAATTAGT | 0.15259 | 0.15123 |
| HNF-1C [T01951] | 710 | 718 | 5.995984 | ATAATTAGT | 0.08392 | 0.08282 |
| HNF-3 [T00370] | 1711 | 1719 | 10.84893 | ACAAAATCC | 0.04578 | 0.05469 |
| HNF-3 [T02277] | 1050 | 1055 | 1.824994 | TTTTTA | 0.48828 | 0.50278 |
| HNF-3alpha [T00371] | 1558 | 1565 | 4.525228 | GTGTTTAC | 0.09155 | 0.09927 |
| HNF-3alpha [T02512] | 750 | 759 | 1.029523 | TTATTTATTT | 0.02289 | 0.02279 |
| HNF-3beta [T01049] | 1225 | 1232 | 0.693573 | AAAAAACA | 0.06104 | 0.07134 |
| HNF-3beta [T02344] | 853 | 859 | 8.430882 | TGTTGCT | 0.12207 | 0.13109 |
| HNF-3beta [T02513] | 483 | 489 | 8.004022 | GGGTTTA | 0.24414 | 0.25336 |
| HNF-3beta [T03256] | 974 | 983 | 4.316993 | CAAACACTCA | 0.03433 | 0.04057 |
| HNF-3gamma [T01050] | 1302 | 1311 | 2.878714 | AGCAAATATA | 0.02289 | 0.0238 |
| HNF-4alpha [T05287] | 1050 | 1061 | 6.275401 | TTTTTAAAGGTC | 0.02289 | 0.02208 |
| HNF-6 [T03296] | 1198 | 1208 | 12.91732 | TTGATATTGAT | 0.04673 | 0.04603 |
| HNF-6alpha [T03257] | 1200 | 1212 | 14.45817 | GATATTGATGGAG | 0.02512 | 0.02515 |
| HNF-6beta [T03258] | 748 | 759 | 14.37233 | ATTTATTTATTT | 0.01502 | 0.01642 |
| HOXA3 [T00378] | 1933 | 1937 | 0.859912 | CACAT | 1.95312 | 1.98165 |
| HOXA4 [T00128] | 711 | 719 | 1.630718 | TAATTAGTA | 0.05341 | 0.05161 |
| HOXD10 [T01425] | 754 | 763 | 4.321431 | TTATTTTATT | 0.03433 | 0.03736 |
| HOXD10 [T01757] | 754 | 763 | 4.321431 | TTATTTTATT | 0.03433 | 0.03736 |
| HOXD10 [T01758] | 754 | 763 | 4.321431 | TTATTTTATT | 0.03433 | 0.03736 |
| HOXD8 [T01426] | 1162 | 1168 | 3.772054 | GGATTAT | 0.48828 | 0.4915 |
| HOXD8 [T01754] | 1162 | 1168 | 3.772054 | GGATTAT | 0.48828 | 0.4915 |
| HOXD9 [T01424] | 754 | 763 | 4.321431 | TTATTTTATT | 0.03433 | 0.03736 |
| HOXD9 [T01755] | 754 | 763 | 4.321431 | TTATTTTATT | 0.03433 | 0.03736 |
| HOXD9 [T01756] | 754 | 763 | 4.321431 | TTATTTTATT | 0.03433 | 0.03736 |
| HSF1 (long) [T01042] | 1277 | 1284 | 6.723057 | TCTAGAAT | 0.18311 | 0.1768 |
| HSF1 (short) [T02104] | 1277 | 1284 | 6.723057 | TCTAGAAT | 0.18311 | 0.1768 |
| HTF [T05026] | 1552 | 1560 | 5.256295 | GCTCACGTG | 0.19836 | 0.19507 |
| IA-1 [T05887] | 358 | 370 | 12.20793 | CCCCAGGGGGCAG | 0.00748 | 0.00777 |
| Ik-1 [T02702] | 1152 | 1164 | 11.87149 | CGTGTACCTGGGA | 0.06446 | 0.06582 |
| INO2 [T01241] | 1445 | 1453 | 9.339767 | ATGTGAAGC | 0.03052 | 0.02969 |
| IPF1 [T02057] | 556 | 563 | 0.604047 | TAATGTGA | 0.12207 | 0.11877 |
| IRF-1 [T00423] | 1749 | 1757 | 3.078495 | AGAAGGAAA | 0.12207 | 0.1215 |
| IRF-2 [T00425] | 1390 | 1402 | 10.98892 | CAGGAAAGTGACC | 0.01115 | 0.01081 |
| IRF-2 [T01491] | 1395 | 1405 | 6.873928 | AAGTGACCAAG | 0.19312 | 0.18773 |
| IRF-3 [T04673] | 1392 | 1398 | 0.238426 | GGAAAGT | 0.24414 | 0.23799 |
| Isl-1 [T01956] | 454 | 462 | 5.955048 | TCCTCTAAT | 0.18311 | 0.17718 |
| JunB [T00436] | 497 | 502 | 5.882353 | TGACCG | 0.48828 | 0.47791 |
| JunD [T00437] | 817 | 823 | 3.911519 | TGACCAC | 0.24414 | 0.24612 |
| JunD [T01978] | 419 | 431 | 9.264391 | TGACTGACTTGCT | 0.0124 | 0.01202 |
| Kr [T00456] | 479 | 489 | 13.23873 | AAATGGGTTTA | 0.01001 | 0.00957 |
| LCR-F1 [T01599] | 1272 | 1277 | 3.719461 | TATCAT | 1.46484 | 1.4345 |
| LEF-1 [T02905] | 679 | 686 | 9.741481 | AGCCAAAG | 0.18311 | 0.18283 |
| LF-A1 [T00467] | 1402 | 1408 | 4.069855 | CAAGGCA | 0.12207 | 0.12333 |
| LIM1 [T04817] | 1877 | 1880 | 0 | CCAA | 7.8125 | 8.20975 |
| Lmo2 [T02251] | 118 | 125 | 1.525983 | TCAGGTGT | 0.09155 | 0.09114 |
| LVb-binding factor [T00477] | 1928 | 1934 | 14.28571 | CAGGACA | 2.56348 | 2.51924 |
| LVc [T00478] | 1618 | 1622 | 0 | CCTGC | 1.95312 | 1.93174 |
| LyF-1 [T00479] | 1004 | 1012 | 2.282962 | CTCCCAAGT | 0.02289 | 0.0235 |
| MAC1 [T01265] | 1486 | 1492 | 0 | GAGCAAA | 0.12207 | 0.12284 |
| MafG [T01437] | 1283 | 1287 | 0 | ATGAC | 1.95312 | 1.91635 |
| mat1-Mc [T01275] | 207 | 219 | 13.92695 | CACAAAGAGACCA | 0.0245 | 0.02786 |
| MATa1 [T00488] | 1930 | 1939 | 9.378876 | GGACACATCA | 0.05531 | 0.0576 |
| MATalpha1 [T00486] | 709 | 720 | 4.961352 | CATAATTAGTAA | 0.01431 | 0.01388 |
| MATalpha2 [T00487] | 1445 | 1450 | 2.576772 | ATGTGA | 0.48828 | 0.48213 |
| Max [T05056] | 1551 | 1561 | 2.404057 | TGCTCACGTGT | 0.01717 | 0.01675 |
| MAZ [T00490] | 1955 | 1965 | 14.42564 | CCCTCACACCC | 0.14019 | 0.16052 |
| MBF1 [T00492] | 1220 | 1230 | 14.63647 | AACAAAAAAAA | 0.23174 | 0.25951 |
| MCB1 [T06035] | 1579 | 1587 | 1.715773 | CAAGGATGC | 0.18311 | 0.18419 |
| MCB2 [T06036] | 1579 | 1587 | 1.715773 | CAAGGATGC | 0.18311 | 0.18419 |
| MCBF [T00499] | 1390 | 1401 | 13.66885 | CAGGAAAGTGAC | 0.02253 | 0.02223 |
| MCM1 [T00500] | 489 | 499 | 13.29393 | ACCTAAGCTGA | 0.04959 | 0.04982 |
| mec-3 [T01076] | 703 | 711 | 4.853037 | ATAATCCAT | 0.04578 | 0.04415 |
| MED8 [T03491] | 1754 | 1762 | 0.406282 | GAAAGAAAT | 0.03052 | 0.03046 |
| MEDEA (MED) [T04379] | 1914 | 1924 | 12.40475 | TGGGAGGCCCT | 0.01717 | 0.01735 |
| MEF1 [T00506] | 184 | 193 | 7.74156 | CTCAACACCC | 0.02289 | 0.02602 |
| MEF-2C/delta8 [T01769] | 1301 | 1311 | 3.686082 | GAGCAAATATA | 0.00858 | 0.00827 |
| MEF-2DAB [T02505] | 1289 | 1300 | 9.58838 | AGATAAATAGGT | 0.03004 | 0.03043 |
| Meis-1a [T03388] | 1470 | 1478 | 3.614643 | TGACAGTGG | 0.18311 | 0.1781 |
| Meis-1b [T03389] | 1470 | 1478 | 3.621437 | TGACAGTGG | 0.16022 | 0.15597 |
| MF3 [T00507] | 1908 | 1912 | 1.755599 | TGGAG | 3.90625 | 4.11268 |
| MIG1 [T00509] | 1698 | 1708 | 6.294055 | CCCCAGCGTAA | 0.01526 | 0.01599 |
| Mitf [T01554] | 614 | 626 | 4.592158 | AGCACATGATGAC | 0.00548 | 0.00562 |
| MNB1a [T01059] | 1428 | 1433 | 0 | AAAAAG | 0.48828 | 0.50487 |
| MRF-2 [T04675] | 1640 | 1649 | 14.08311 | GAATACGTTC | 0.07248 | 0.06986 |
| mtTFA [T04783] | 442 | 451 | 3.812478 | TGATAAACCA | 0.12016 | 0.11643 |
| muEBP-C2 [T00215] | 796 | 801 | 0 | CACATG | 0.48828 | 0.48411 |
| MYB2 [T02536] | 1328 | 1332 | 0 | AACTG | 1.95312 | 1.91635 |
| MYBAS1 [T05553] | 1995 | 1999 | 1.224657 | GCCCC | 1.95312 | 2.00558 |
| Myf-3 [T00519] | 670 | 677 | 1.122441 | GCAGCTGA | 0.54932 | 0.54432 |
| Myf-5 [T00521] | 886 | 898 | 11.87149 | TGCTGCTGCTGCT | 0.06446 | 0.06376 |
| MyoD [T00525] | 670 | 677 | 1.122441 | GCAGCTGA | 0.54932 | 0.54432 |
| MyoD [T00526] | 368 | 375 | 3.625964 | CAGCAGAG | 0.06104 | 0.05974 |
| MyoD [T01128] | 670 | 677 | 1.122441 | GCAGCTGA | 0.54932 | 0.54541 |
| myogenin [T00528] | 1457 | 1463 | 0.482381 | CAGCAGG | 0.24414 | 0.23944 |
| MZF-1 [T00529] | 1717 | 1723 | 2.559408 | TCCCCAG | 0.48828 | 0.48796 |
| NBF [T00951] | 1445 | 1453 | 10.48529 | ATGTGAAGC | 0.03052 | 0.02969 |
| NBF [T01190] | 1445 | 1453 | 10.48529 | ATGTGAAGC | 0.03052 | 0.02969 |
| NF-1 (-like proteins) [T00601] | 1088 | 1095 | 2.05371 | CTAGGCTG | 0.12207 | 0.12356 |
| NF-1 [T00535] | 526 | 534 | 3.696156 | TCATGCCAA | 0.08392 | 0.08395 |
| NF-1 [T00536] | 638 | 645 | 4.730993 | TTGGCCAG | 0.12207 | 0.13381 |
| NF-1 [T00537] | 1856 | 1860 | 0.686673 | GCCAG | 1.95312 | 1.93187 |
| NF-1 [T00538] | 47 | 54 | 0 | TTGGAATG | 0.12207 | 0.12668 |
| NF-1 [T00539] | 680 | 684 | 0 | GCCAA | 1.95312 | 1.98986 |
| NF-1 [T01298] | 1895 | 1900 | 2.586354 | GTCCAG | 0.48828 | 0.47791 |
| NF-1/L [T00599] | 891 | 903 | 13.83907 | CTGCTGCTGTTCT | 0.05043 | 0.04932 |
| NF-AT1 [T00550] | 604 | 612 | 2.41749 | GGAAAGTAT | 0.06866 | 0.06652 |
| NF-AT1 [T01944] | 295 | 301 | 0.59313 | GGAAAAC | 0.24414 | 0.2452 |
| NF-AT1 [T01948] | 1392 | 1399 | 2.13792 | GGAAAGTG | 0.18311 | 0.18185 |
| NF-AT2 [T01945] | 1753 | 1761 | 5.673151 | GGAAAGAAA | 0.09918 | 0.09727 |
| NF-AT3 [T02462] | 295 | 304 | 5.555555 | GGAAAACAAT | 0.09155 | 0.08966 |
| NF-AT4 [T01946] | 757 | 768 | 14.00105 | TTTTATTTTTCA | 0.01717 | 0.01734 |
| NF-AT4 [T01949] | 1257 | 1264 | 7.507392 | GAATTTGT | 0.61035 | 0.60496 |
| NFdeltaE3A [T00976] | 201 | 210 | 8.086215 | CCAGACCACA | 0.07439 | 0.08557 |
| NF-E4 [T00560] | 1783 | 1792 | 6.14045 | CCTAAGCCTG | 0.11444 | 0.11284 |
| NFI/CTF [T00094] | 86 | 93 | 8.241664 | CTGGTTGG | 0.18311 | 0.2104 |
| NF-InsE2 [T00585] | 527 | 537 | 13.0894 | CATGCCAAGTG | 0.03433 | 0.03368 |
| NF-InsE3 [T00586] | 527 | 537 | 13.0894 | CATGCCAAGTG | 0.03433 | 0.03368 |
| NF-kappaB [T00588] | 1919 | 1928 | 6.303258 | GGCCCTTCCC | 0.01335 | 0.01302 |
| NF-kappaB1 [T00593] | 1414 | 1425 | 6.706586 | GGGGAGCCTCCA | 0.00858 | 0.00855 |
| NF-muNR [T01083] | 742 | 752 | 14.65974 | TCATTCATTTA | 0.1545 | 0.15429 |
| NF-X3 [T01514] | 829 | 836 | 12.06902 | CAGACCTC | 0.12207 | 0.12306 |
| NF-Y [T00150] | 1346 | 1353 | 3.105715 | GGCCCAAT | 0.18311 | 0.18268 |
| NF-Y [T00613] | 1348 | 1356 | 9.235602 | CCCAATTCA | 0.09155 | 0.09354 |
| NHP-1 [T00621] | 1858 | 1864 | 4.484808 | CAGGTCT | 0.24414 | 0.23799 |
| NIT2 [T00627] | 1286 | 1293 | 5.771507 | ACCAGATA | 0.36621 | 0.35991 |
| Nkx2-1 [T00856] | 1655 | 1660 | 0.762554 | GTCAAG | 0.48828 | 0.47598 |
| Nkx2-1 [T00857] | 198 | 204 | 0 | CCTCCAG | 0.12207 | 0.12379 |
| Nkx2-2 [T02384] | 179 | 190 | 14.36927 | AACCACTCAACA | 0.07236 | 0.07147 |
| Nkx2-5 [T01675] | 1777 | 1785 | 8.861753 | CACTTCCCT | 0.18311 | 0.18026 |
| Nkx6-2 [T02050] | 1290 | 1297 | 1.162791 | GATAAATA | 0.18311 | 0.17686 |
| N-Myc [T01445] | 1555 | 1560 | 0 | CACGTG | 0.48828 | 0.47791 |
| Nrf2:MafK [T05666] | 1850 | 1856 | 6.857881 | ACAGGAG | 0.24414 | 0.24257 |
| OCSBF-1 [T02999] | 1553 | 1562 | 10.71429 | CTCACGTGTT | 0.12207 | 0.11947 |
| Oct-B1 [T00545] | 726 | 733 | 12.5 | ATGTGCAT | 0.73242 | 0.71933 |
| Olf-1 [T01040] | 1677 | 1689 | 13.40636 | CACTCCTAGAGGA | 0.03767 | 0.03746 |
| Opaque-2 [T00668] | 1552 | 1560 | 6.217121 | GCTCACGTG | 0.13733 | 0.13523 |
| OSH15 [T05493] | 1469 | 1476 | 5.364689 | GTGACAGT | 0.15259 | 0.14911 |
| P (long form) [T01590] | 1491 | 1500 | 13.45288 | AACCTACTCT | 0.06866 | 0.07213 |
| p300 [T01427] | 1853 | 1857 | 3.869475 | GGAGC | 1.95312 | 1.93174 |
| p53 [T00671] | 1979 | 1985 | 10.91783 | AGGGCCC | 0.12207 | 0.1202 |
| Pax-2 [T01823] | 1281 | 1287 | 2.578483 | GAATGAC | 0.36621 | 0.35723 |
| Pax-2a [T00678] | 1734 | 1740 | 3.075094 | CCTGTGC | 0.12207 | 0.11972 |
| Pax-4a [T02983] | 1265 | 1270 | 1.167421 | TTATTT | 0.97656 | 0.98306 |
| Pax-5 [T00070] | 1026 | 1032 | 7.762216 | GCGTGCA | 0.24414 | 0.24401 |
| Pax-5 [T01201] | 1699 | 1705 | 2.389902 | CCCAGCG | 0.61035 | 0.6094 |
| Pax-6 [T00681] | 938 | 952 | 10.4323 | ATGTCCTGGAACTCA | 0.00014 | 0.00013 |
| Pax-6 [T00682] | 1895 | 1899 | 3.743085 | GTCCA | 7.8125 | 7.77763 |
| Pax-6 [T01122] | 792 | 802 | 7.791946 | TTCACACATGC | 0.04959 | 0.04854 |
| Pax-8 [T01828] | 1622 | 1627 | 5.938178 | CAACTC | 0.48828 | 0.5087 |
| Pax-9a [T03593] | 1538 | 1544 | 3.29756 | CACCAGT | 0.24414 | 0.24253 |
| Pax-9b [T03594] | 1538 | 1544 | 3.29756 | CACCAGT | 0.24414 | 0.24253 |
| PBF [T02693] | 1309 | 1316 | 7.830456 | ATAGAAGG | 0.12207 | 0.11875 |
| Pbx1b [T02087] | 416 | 425 | 8.356737 | CTGTGACTGA | 0.09155 | 0.08911 |
| Pbx1b [T02088] | 1972 | 1982 | 10.94603 | TGGAAGAAGGG | 0.0515 | 0.05475 |
| PEA3 [T00684] | 1750 | 1757 | 1.069584 | GAAGGAAA | 0.06104 | 0.05927 |
| PEA3 [T00685] | 936 | 943 | 0.428942 | GGATGTCC | 0.12207 | 0.12743 |
| PHO4 [T00690] | 1480 | 1491 | 13.2639 | CCCCACGAGCAA | 0.02253 | 0.02386 |
| PHR1 [T05369] | 111 | 119 | 12.87666 | TGCATACTC | 0.54932 | 0.53484 |
| PKNOX1 [T04122] | 1466 | 1476 | 11.26752 | ATGGTGACAGT | 0.05722 | 0.05579 |
| POU1F1 [T00707] | 748 | 757 | 13.79464 | ATTTATTTAT | 0.16022 | 0.15448 |
| POU1F1a [T00691] | 580 | 584 | 5.663955 | ATTGT | 1.95312 | 1.97387 |
| POU1F1b [T01516] | 132 | 139 | 3.500065 | ACATTTAT | 0.27466 | 0.26392 |
| POU1F1c [T01902] | 132 | 139 | 3.500065 | ACATTTAT | 0.27466 | 0.26392 |
| POU2F1 [T00641] | 724 | 733 | 13.62662 | ACATGTGCAT | 0.64087 | 0.62676 |
| POU2F1 [T00959] | 726 | 733 | 12.5 | ATGTGCAT | 0.73242 | 0.71933 |
| POU2F1 [T01466] | 727 | 734 | 1.994941 | TGTGCATA | 0.06104 | 0.05926 |
| POU2F1a [T00644] | 528 | 535 | 12.5555 | ATGCCAAG | 0.18311 | 0.17977 |
| POU2F1b [T01862] | 726 | 736 | 12.59484 | ATGTGCATATA | 0.73242 | 0.71933 |
| POU2F1c [T01863] | 726 | 736 | 12.59484 | ATGTGCATATA | 0.73242 | 0.71933 |
| POU2F2 (Oct-2.1) [T00646] | 1267 | 1274 | 5.153746 | ATTTCTAT | 0.03052 | 0.02964 |
| POU2F2 (Oct-2.1) [T01864] | 723 | 733 | 13.38827 | CACATGTGCAT | 0.48065 | 0.46849 |
| POU2F2 (Oct-2.1) [T01870] | 723 | 733 | 13.38827 | CACATGTGCAT | 0.48065 | 0.46849 |
| POU2F2 (Oct-2.3) [T01865] | 723 | 733 | 13.38827 | CACATGTGCAT | 0.48065 | 0.46849 |
| POU2F2 (Oct-2.4) [T01866] | 723 | 733 | 13.38827 | CACATGTGCAT | 0.48065 | 0.46849 |
| POU2F2 (Oct-2.6) [T01867] | 723 | 733 | 13.38827 | CACATGTGCAT | 0.48065 | 0.46849 |
| POU2F2 [T00647] | 723 | 733 | 13.05187 | CACATGTGCAT | 0.40054 | 0.3914 |
| POU2F2 [T00648] | 723 | 733 | 13.38827 | CACATGTGCAT | 0.48065 | 0.46849 |
| POU2F2 [T01032] | 1636 | 1643 | 11.98268 | ATGGGAAT | 0.12207 | 0.11854 |
| POU2F2B [T00662] | 726 | 733 | 12.5 | ATGTGCAT | 0.73242 | 0.71933 |
| POU2F2B [T00964] | 723 | 734 | 14.2127 | CACATGTGCATA | 0.18525 | 0.18033 |
| POU2F2B [T01871] | 726 | 733 | 12.5 | ATGTGCAT | 0.73242 | 0.71933 |
| POU2F2C [T00665] | 1636 | 1643 | 11.98268 | ATGGGAAT | 0.12207 | 0.11854 |
| POU3F1 [T00656] | 726 | 736 | 12.86448 | ATGTGCATATA | 0.20599 | 0.19928 |
| POU3F1 [T00969] | 1063 | 1069 | 2.289542 | TTAAAAT | 0.36621 | 0.35246 |
| POU3F2 [T00630] | 1759 | 1765 | 8.108417 | AAATGGT | 0.24414 | 0.23702 |
| POU4F1(l) [T01877] | 709 | 718 | 9.210675 | CATAATTAGT | 0.2861 | 0.27656 |
| POU5F1 (Oct-5) [T00653] | 726 | 734 | 12.87666 | ATGTGCATA | 0.54932 | 0.53733 |
| POU5F1 [T00651] | 726 | 733 | 13.64864 | ATGTGCAT | 0.64087 | 0.63081 |
| POU6F1 [T04470] | 1193 | 1203 | 7.870358 | CATACTTGATA | 0.07153 | 0.07023 |
| PPAR-alpha [T00694] | 1944 | 1950 | 1.122687 | TGACCTA | 0.48828 | 0.47598 |
| PPAR-alpha:RXR-alpha [T05221] | 355 | 365 | 6.51544 | GAGCCCCAGGG | 0.03719 | 0.03679 |
| PR A [T01661] | 1329 | 1335 | 2.830632 | ACTGTTT | 0.24414 | 0.24515 |
| PR B [T00696] | 1329 | 1335 | 2.830632 | ACTGTTT | 0.24414 | 0.24515 |
| PR B [T00697] | 1840 | 1846 | 2.069021 | AGGAACA | 0.24414 | 0.2416 |
| PR-alpha [T01660] | 1659 | 1667 | 1.918551 | AGAACACGG | 0.27466 | 0.28483 |
| PR-beta [T00698] | 1659 | 1667 | 1.918551 | AGAACACGG | 0.27466 | 0.28483 |
| Prd [T00699] | 711 | 717 | 8.430882 | TAATTAG | 0.12207 | 0.1178 |
| PTF1 [T01227] | 1909 | 1919 | 7.881492 | GGAGATGGGAG | 0.03433 | 0.03523 |
| PTF1-beta [T00701] | 1909 | 1918 | 6.461772 | GGAGATGGGA | 0.06866 | 0.07046 |
| Pu box binding factor [T00704] | 759 | 768 | 1.112096 | TTATTTTTCA | 0.01144 | 0.01138 |
| PU.1 [T00702] | 1251 | 1259 | 1.42401 | TGTGAGGAA | 0.11444 | 0.11301 |
| PU.1 [T02068] | 1254 | 1263 | 2.219236 | GAGGAATTTG | 0.02289 | 0.02234 |
| PUR alpha [T05167] | 999 | 1008 | 12.13723 | TCTGCCTCCC | 0.09346 | 0.09681 |
| PUR beta [T05172] | 999 | 1008 | 12.13723 | TCTGCCTCCC | 0.09346 | 0.09681 |
| R [T00710] | 1178 | 1186 | 9.89927 | CACCAGGCC | 0.10681 | 0.1099 |
| R1 [T00711] | 1959 | 1970 | 9.060757 | CACACCCTCACG | 0.03719 | 0.03918 |
| R2 [T00712] | 465 | 470 | 1.824994 | TCCAGC | 0.48828 | 0.48603 |
| RAR-alpha1 [T00719] | 1650 | 1659 | 4.599928 | CCAAGGTCAA | 0.01526 | 0.01519 |
| RAR-beta [T00721] | 1398 | 1405 | 7.533632 | TGACCAAG | 0.12207 | 0.12127 |
| RAR-beta:RXR-alpha [T05420] | 1894 | 1905 | 14.95599 | TGTCCAGAGCCC | 0.20957 | 0.20854 |
| RAR-beta2 [T01326] | 604 | 615 | 14.97845 | GGAAAGTATCAG | 0.13852 | 0.13489 |
| RAR-gamma [T00720] | 1809 | 1816 | 0.919959 | AGCTCACC | 0.09155 | 0.09151 |
| RC2 [T00724] | 1763 | 1768 | 13.78485 | GGTACA | 1.95312 | 1.95674 |
| RCS1 [T03538] | 1523 | 1534 | 14.16218 | GTGGATGCATTC | 0.02754 | 0.02811 |
| RelA [T00594] | 1920 | 1929 | 12.11484 | GCCCTTCCCA | 0.05341 | 0.05295 |
| RelA [T00595] | 1779 | 1789 | 14.46797 | CTTCCCTAAGC | 0.03433 | 0.03429 |
| RF2a [T02811] | 578 | 587 | 5.850141 | CCATTGTGGC | 0.0515 | 0.05189 |
| RFX1 [T01673] | 1083 | 1091 | 5.101965 | GTTGCCTAG | 0.05341 | 0.05519 |
| RITA-1 [T02786] | 1555 | 1562 | 2.808502 | CACGTGTT | 0.09155 | 0.08962 |
| ROM1 [T02809] | 1806 | 1814 | 11.11111 | GCCAGCTCA | 0.20599 | 0.2168 |
| ROM2 [T02810] | 1806 | 1814 | 11.11111 | GCCAGCTCA | 0.20599 | 0.2168 |
| RORalpha1 [T01527] | 1649 | 1658 | 5.215967 | CCCAAGGTCA | 0.03242 | 0.0314 |
| RORalpha2 [T01528] | 1291 | 1302 | 11.94112 | ATAAATAGGTGA | 0.04578 | 0.04439 |
| ROX1 [T01286] | 298 | 307 | 1.883357 | AAACAATGGC | 0.02098 | 0.02116 |
| RP58 [T05040] | 1930 | 1940 | 13.89724 | GGACACATCAG | 0.16022 | 0.15675 |
| RPN4 [T04539] | 582 | 590 | 11.11111 | TGTGGCAAA | 0.20599 | 0.20146 |
| RXR-alpha [T01345] | 1650 | 1658 | 0.699444 | CCAAGGTCA | 0.04578 | 0.04466 |
| RXR-beta [T01332] | 1653 | 1662 | 3.587401 | AGGTCAAGAA | 0.02289 | 0.02227 |
| S8 [T01483] | 710 | 716 | 1.44008 | ATAATTA | 0.24414 | 0.23513 |
| SBF-1 [T00739] | 1019 | 1027 | 7.662751 | ATTAAAAGC | 0.06104 | 0.0589 |
| SF-1 [T01147] | 1742 | 1750 | 3.723894 | CAAGGACAG | 0.03052 | 0.03083 |
| SF-1 [T02769] | 1649 | 1659 | 1.383409 | CCCAAGGTCAA | 0.01144 | 0.0112 |
| SF-1 [T04014] | 1649 | 1658 | 0.524236 | CCCAAGGTCA | 0.01144 | 0.01128 |
| SGF-3 [T00746] | 754 | 764 | 14.39275 | TTATTTTATTT | 0.00858 | 0.00885 |
| Smad3 [T04096] | 1403 | 1412 | 0.777683 | AAGGCAGACA | 0.0267 | 0.0273 |
| Smad4 [T04292] | 1403 | 1413 | 3.483393 | AAGGCAGACAC | 0.01812 | 0.01827 |
| Sox13 [T02420] | 580 | 587 | 0.673105 | ATTGTGGC | 0.54932 | 0.56168 |
| Sox17 [T06029] | 214 | 224 | 4.313106 | AGACCAACAAA | 0.00191 | 0.00229 |
| Sox2 [T01836] | 1260 | 1266 | 0 | TTTGTTT | 0.12207 | 0.1427 |
| Sp1 [T00752] | 1990 | 1999 | 3.390871 | GGGCCGCCCC | 0.05531 | 0.06014 |
| Sp1 [T00753] | 1993 | 1998 | 0 | CCGCCC | 0.48828 | 0.51482 |
| Sp1 [T00754] | 1990 | 1999 | 2.278889 | GGGCCGCCCC | 0.06104 | 0.06522 |
| Sp1 [T00755] | 1991 | 1999 | 1.626329 | GGCCGCCCC | 0.03815 | 0.0405 |
| Sp1 [T00759] | 989 | 997 | 10.54487 | TCCGCCTGC | 0.16022 | 0.15986 |
| Sp3 [T02338] | 364 | 372 | 7.890688 | GGGGCAGCA | 0.03052 | 0.03024 |
| Sp3 [T02419] | 403 | 413 | 6.239006 | TCACATCCCTG | 0.04005 | 0.04057 |
| SPF1 [T03975] | 806 | 812 | 0 | AATAGTA | 0.12207 | 0.11781 |
| Spz1 [T04668] | 1962 | 1968 | 2.910575 | ACCCTCA | 0.48828 | 0.55292 |
| SRF [T00766] | 708 | 719 | 12.45055 | CCATAATTAGTA | 0.01216 | 0.01198 |
| SRF [T05114] | 1337 | 1346 | 3.773585 | CTATAATGGG | 0.06104 | 0.05899 |
| SRY [T00997] | 851 | 857 | 2.335519 | GTTGTTG | 0.24414 | 0.27489 |
| Sry-delta [T00767] | 1907 | 1919 | 14.15729 | GTGGAGATGGGAG | 0.01121 | 0.01113 |
| Staf [T02247] | 1629 | 1640 | 12.45933 | GTGTCCAATGGG | 0.00572 | 0.0061 |
| STAT1 [T01575] | 766 | 778 | 8.152314 | TCAGTGCTGGGAA | 0.00638 | 0.00631 |
| STAT1beta [T01573] | 1749 | 1758 | 9.807397 | AGAAGGAAAG | 0.14877 | 0.14592 |
| STAT4 [T01577] | 1922 | 1927 | 5.882353 | CCTTCC | 0.48828 | 0.48597 |
| STAT5A [T04683] | 1973 | 1976 | 0 | GGAA | 7.8125 | 7.68075 |
| STAT6 [T01581] | 1924 | 1932 | 6.546023 | TTCCCAGGA | 0.03052 | 0.0295 |
| STE12 [T00772] | 809 | 816 | 14.91228 | AGTATCAT | 1.28174 | 1.28215 |
| Stra13 [T02327] | 1554 | 1564 | 5.630873 | TCACGTGTTTA | 0.01049 | 0.0103 |
| Su(H) [T01615] | 1645 | 1657 | 14.05099 | CGTTCCCAAGGTC | 0.01037 | 0.01012 |
| SXR:RXR-alpha [T05670] | 533 | 541 | 6.176442 | AAGTGCAGA | 0.15259 | 0.14808 |
| T3R-alpha [T00838] | 1278 | 1289 | 10.42539 | CTAGAATGACCA | 0.03505 | 0.03518 |
| T3R-alpha [T00841] | 1284 | 1292 | 6.131888 | TGACCAGAT | 0.04578 | 0.04431 |
| T3R-alpha [T01351] | 1944 | 1952 | 7.213398 | TGACCTAGA | 0.15259 | 0.14849 |
| T3R-alpha1 [T01152] | 1284 | 1294 | 13.69019 | TGACCAGATAA | 0.18311 | 0.17906 |
| T3R-beta1 [T00851] | 1281 | 1291 | 13.06862 | GAATGACCAGA | 0.18024 | 0.17593 |
| T3R-beta1 [T00853] | 1649 | 1658 | 9.95106 | CCCAAGGTCA | 0.04578 | 0.04455 |
| T3R-beta2 [T01350] | 816 | 824 | 5.453423 | TTGACCACA | 0.04578 | 0.04641 |
| TAF [T00778] | 1965 | 1971 | 11.99205 | CTCACGA | 1.95312 | 1.9531 |
| T-Ag [T00788] | 998 | 1006 | 3.069503 | CTCTGCCTC | 0.09155 | 0.09122 |
| Tal-1 [T01799] | 119 | 127 | 4.498518 | CAGGTGTGT | 0.09155 | 0.0917 |
| TBP [T00794] | 1338 | 1345 | 5.261567 | TATAATGG | 0.54932 | 0.53155 |
| TBP [T00798] | 749 | 756 | 3.734581 | TTTATTTA | 0.03052 | 0.03103 |
| TCF-1(P) [T01109] | 832 | 841 | 0.939405 | ACCTCTTGTT | 0.01526 | 0.01494 |
| TCF-1A [T00999] | 389 | 397 | 12.45419 | AGCCCAAAG | 0.03052 | 0.03083 |
| TCF-2 [T01110] | 915 | 924 | 13.45891 | GGGTTCTCTC | 0.20027 | 0.20956 |
| TCF-4E [T02878] | 680 | 686 | 12.60477 | GCCAAAG | 0.48828 | 0.50886 |
| TF68 [T00809] | 619 | 628 | 12.54884 | ATGATGACAA | 0.25749 | 0.25478 |
| TFIIB [T00818] | 1374 | 1381 | 1.13481 | TCTGAGAG | 0.06104 | 0.05988 |
| TFIID [T00820] | 1338 | 1345 | 6.54118 | TATAATGG | 0.61035 | 0.59399 |
| TFII-I [T00824] | 933 | 943 | 11.0483 | ACTGGATGTCC | 0.00954 | 0.00931 |
| TGA1a [T00829] | 1644 | 1649 | 3.461701 | ACGTTC | 0.48828 | 0.47598 |
| TGGCA-binding protein [T00832] | 1630 | 1636 | 1.451226 | TGTCCAA | 0.36621 | 0.35982 |
| TGIF [T04076] | 1535 | 1542 | 2.101752 | TGACACCA | 0.39673 | 0.39375 |
| TGT3 [T00833] | 1327 | 1339 | 3.367519 | AAACTGTTTGCTA | 0.00197 | 0.00211 |
| Tll [T00789] | 1019 | 1024 | 0.107555 | ATTAAA | 1.95312 | 1.90095 |
| TMF [T00835] | 1338 | 1344 | 2.605613 | TATAATG | 0.48828 | 0.47215 |
| TOXE [T05645] | 980 | 989 | 9.256836 | CTCAAGAGAT | 0.02289 | 0.02301 |
| TRM1 [T05311] | 1392 | 1399 | 0 | GGAAAGTG | 0.09155 | 0.08887 |
| Ttk 69K [T00843] | 258 | 265 | 13.58591 | GGTCATGC | 0.64087 | 0.63548 |
| Ubx [T00863] | 1340 | 1345 | 0 | TAATGG | 0.48828 | 0.48213 |
| UME6 [T01247] | 1481 | 1491 | 14.55272 | CCCACGAGCAA | 0.13733 | 0.13698 |
| unc-86 [T01882] | 1305 | 1309 | 0 | AAATA | 1.95312 | 1.96612 |
| USF [T00873] | 1552 | 1560 | 6.658644 | GCTCACGTG | 0.20599 | 0.2014 |
| USF1 [T00874] | 1553 | 1561 | 2.082246 | CTCACGTGT | 0.09918 | 0.09756 |
| USF-1 [T00875] | 1984 | 1990 | 4.610088 | CCACAGG | 0.48828 | 0.48803 |
| USF-1 [T00877] | 103 | 113 | 11.57685 | CCTACACATGC | 0.01335 | 0.01309 |
| USF2 [T00878] | 493 | 501 | 11.04964 | AAGCTGACC | 0.09155 | 0.09004 |
| USF2 [T02115] | 1944 | 1949 | 1.796148 | TGACCT | 0.48828 | 0.47598 |
| USF2b [T02377] | 493 | 500 | 5.687009 | AAGCTGAC | 0.73242 | 0.72091 |
| VDR [T00885] | 1962 | 1965 | 0 | ACCC | 7.8125 | 8.24185 |
| v-Myb [T00895] | 663 | 670 | 6.694042 | AGAAACGG | 0.73242 | 0.72409 |
| Vpr [T02399] | 1675 | 1682 | 0 | GCCACTCC | 0.06104 | 0.06368 |
| WIZZ [T03807] | 182 | 192 | 14.43282 | CACTCAACACC | 0.23174 | 0.24112 |
| WT1 I [T01840] | 1845 | 1851 | 0 | CACACAC | 0.12207 | 0.14435 |
| XPF-1 [T00906] | 1983 | 1991 | 11.42573 | CCCACAGGG | 0.27466 | 0.27056 |
| Xvent-1 [T04665] | 209 | 216 | 3.950759 | CAAAGAGA | 0.24414 | 0.25217 |
| YAP1 [T00028] | 710 | 722 | 7.558331 | ATAATTAGTAAGA | 0.01717 | 0.01677 |
| Yi [T00913] | 1322 | 1331 | 9.523389 | CCCACAAACT | 0.12589 | 0.13335 |
| YY1 [T00278] | 268 | 274 | 4.543742 | CCATCCT | 0.12207 | 0.12328 |
| YY1 [T00865] | 1342 | 1347 | 2.437874 | ATGGGG | 0.48828 | 0.51069 |
| YY1 [T00915] | 1758 | 1764 | 2.647969 | GAAATGG | 0.12207 | 0.11875 |
| YY1 [T04970] | 905 | 913 | 2.349284 | GGTGAGACA | 0.06104 | 0.0595 |
| Zen-1 [T00917] | 1015 | 1024 | 5.592134 | TGGTATTAAA | 0.03433 | 0.03309 |
| Zen-2 [T02099] | 1015 | 1024 | 5.592134 | TGGTATTAAA | 0.03433 | 0.03309 |
| Zeste [T00918] | 1772 | 1778 | 4.712018 | CACTCCA | 0.48828 | 0.51069 |
| Zeste [T02100] | 1772 | 1778 | 4.14974 | CACTCCA | 0.48828 | 0.51069 |
| Zic1 [T04669] | 1962 | 1965 | 0 | ACCC | 7.8125 | 8.24185 |
| Zic3 [T04671] | 1962 | 1965 | 0 | ACCC | 7.8125 | 8.24185 |
| Zta [T00923] | 719 | 726 | 8.780461 | AAGACACA | 0.09155 | 0.09955 |

**Supplementary Data 3. The predicted transcription factor binding sites in pri-miR-135a-1 promoter by LASAGNA.**

| Name | Sequence | Position | Strand | Score | p-value | E-value |
| --- | --- | --- | --- | --- | --- | --- |
| AhR:Arnt(M00235) | TATTAAAAGCGTGCAC | 1018 | + | 13.86 | 0.0001 | 0.199 |
| AP-1(M00172) | GATGACAAAGT | 621 | + | 9.44 | 0.0006 | 1.19 |
| AP-1(M00173) | GATGACAAAGT | 621 | + | 9.83 | 0.000475 | 0.95 |
| AP-1(M00174) | TGTGACTGACT | 417 | + | 8.88 | 0.000625 | 1.24 |
| AP-1(M00188) | GATGACAAAGT | 621 | + | 9.66 | 0.000225 | 0.45 |
| AP-1(M00199) | CTGTCTCA | 907 | - | 11.23 | 0.000875 | 1.74 |
| AP-1(M00517) | CCCTGTCTCACCA | 904 | - | 7.88 | 0.000825 | 1.64 |
| AP-2(M00189) | ATTCTAGATGAT | 1273 | - | 8.62 | 0.00025 | 0.5 |
| AP-2alpha(M00469) | GCCTTGGGC | 329 | + | 7.94 | 0.0004 | 0.8 |
| AP-2gamma(M00470) | GCCTCAAAC | 970 | + | 8.22 | 0.000425 | 0.85 |
| AP-2rep(M00468) | CAGTGGA | 1546 | - | 7.54 | 0.00015 | 0.299 |
| AP-4(M00005) | TGAACCTGCTTTGGGCTT | 388 | - | 11.15 | 0.000225 | 0.45 |
| AP-4(M00175) | ATCAGCTCTG | 1936 | + | 10.69 | 5.00E-05 | 0.1 |
| AP-4(M00176) | CTCAGCTGCC | 669 | - | 10.57 | 5.00E-05 | 0.1 |
| AREB6(M00412) | CACACACCTGAGT | 116 | - | 13.74 | 2.50E-05 | 0.05 |
| AREB6(M00413) | ACACACCTGAGT | 116 | - | 11.45 | 2.50E-05 | 0.05 |
| AREB6(M00414) | GTGTACCTGGGA | 1153 | + | 9.41 | 0.000325 | 0.65 |
| AREB6(M00415) | CCGTTTCTC | 662 | - | 7.87 | 0.000675 | 1.34 |
| Arnt(M00236) | GTAAACACGTGAGCAG | 1550 | - | 12.23 | 0.0001 | 0.199 |
| Arnt(M00539) | CACTGCTCACGTGTTTACCT | 1548 | + | 12.07 | 0 | 0 |
| ARP-1(M00155) | TGAGTGTTTGAGGCCA | 968 | - | 9.52 | 5.00E-05 | 0.099 |
| ATF(M00017) | CTGTGAGGTAAACA | 1559 | - | 8.08 | 0.000775 | 1.54 |
| Bach2(M00490) | CCTGAGTC | 652 | + | 15.25 | 0.000375 | 0.75 |
| Barbie Box(M00238) | ATCAAAAGCCCAAAG | 383 | + | 10.97 | 5.00E-05 | 0.099 |
| Brachyury(M00150) | TTCCTCACAGTTAGAGCTCAATAG | 1236 | - | -3.44 | 0.000925 | 1.83 |
| BSAP(M00143) | CTGAAAAGCACCCATGGGTACACTGTGG | 1499 | + | 11.88 | 2.50E-05 | 0.049 |
| BSAP(M00144) | CACATGCATACTCAGGTGTGTGCACACA | 107 | + | 6.57 | 0.000975 | 1.92 |
| C/EBP(M00159) | TGTGTGCACACAC | 122 | - | 9.21 | 0.000175 | 0.35 |
| C/EBPalpha(M00116) | GGTTTACCTAAGCT | 484 | + | 8.22 | 0.000675 | 1.34 |
| C/EBPbeta(M00109) | AGCTTAGGTAAACC | 484 | - | 8.76 | 0.00065 | 1.29 |
| C/EBPbeta(M00117) | AGCGTGCACCACCA | 1025 | + | 8.93 | 0.00075 | 1.49 |
| cap(M00253) | TCAGTGGT | 278 | - | 7.42 | 2.50E-05 | 0.05 |
| CCAAT box(M00254) | TATAGCCATTCA | 733 | + | 12.33 | 2.50E-05 | 0.05 |
| Cdc5(M00478) | GCTTTAATGTGA | 552 | + | 9.13 | 0.00035 | 0.7 |
| CDP(M00095) | CTAATTATGGAT | 706 | - | 8.49 | 0.000375 | 0.75 |
| CdxA(M00100) | CTTTACG | 1704 | - | 6.81 | 0 | 0 |
| c-Ets-1(p54)(M00032) | ACTGGATGTC | 933 | + | 10.86 | 0.00015 | 0.299 |
| c-Ets-1(p54)(M00074) | CTGGATGTCC | 934 | + | 12.76 | 0.00095 | 1.89 |
| c-Myc:Max(M00118) | TGCTCACGTGTTTA | 1551 | + | 8.13 | 7.50E-05 | 0.149 |
| c-Myc:Max(M00123) | TAGCATGTGTG | 794 | - | 14.54 | 0.000275 | 0.55 |
| c-Myc:Max(M00615) | AGGTAAACACGTGAGCAGTG | 1548 | - | 12.59 | 5.00E-05 | 0.099 |
| COMP1(M00057) | TAGTATCATTGACCACA | 808 | + | 22.53 | 0.000125 | 0.247 |
| COUP-TF, HNF-4(M00158) | TGACCTTGGGAACG | 1645 | - | 10.11 | 0.00025 | 0.5 |
| CP2(M00072) | GCCCTTCCCAG | 1920 | + | 8.31 | 0.00065 | 1.29 |
| CREB(M00177) | TGTGAGGTAAAC | 1560 | - | 8.1 | 0.0008 | 1.59 |
| CREB(M00178) | TGTGAGGTAAAC | 1560 | - | 8.27 | 0.000975 | 1.94 |
| c-Rel(M00053) | TGGGTTTACC | 482 | + | 8.51 | 0.00055 | 1.1 |
| deltaEF1(M00073) | ACACACCTGA | 118 | - | 13.63 | 0.000125 | 0.249 |
| E2F(M00024) | ATGGCTGGAGAATTA | 459 | - | 7.6 | 0.000575 | 1.14 |
| E47(M00071) | CTAGCATGTGTGAAG | 791 | - | 12.36 | 0.000425 | 0.84 |
| En-1(M00396) | ATAATCC | 703 | + | 6.83 | 0.000325 | 0.65 |
| ER(M00191) | CCCCGTGTTCTTGACCTTG | 1651 | - | 8.22 | 0.000925 | 1.83 |
| Evi-1(M00011) | ACCAGATAA | 1286 | + | 7.45 | 0.000675 | 1.34 |
| Evi-1(M00079) | TGACCAGATAA | 1284 | + | 9.29 | 0.0002 | 0.4 |
| Evi-1(M00080) | TGACCAGATAA | 1284 | + | 7.53 | 0.000725 | 1.44 |
| Evi-1(M00081) | GGCTAAGTCTGGTCA | 248 | + | 9.59 | 0.000125 | 0.248 |
| Evi-1(M00082) | TGACCAGATA | 1284 | + | 10.72 | 0.0008 | 1.59 |
| FAC1(M00456) | GCCCACAAACTGTT | 1321 | + | 8.69 | 0.0001 | 0.199 |
| FOXD3(M00130) | CTATATTTGCTC | 1301 | - | 9.04 | 0.0002 | 0.4 |
| FOXJ2(M00422) | TGTGAGGTAAACACGTGA | 1554 | - | 10.52 | 0.00015 | 0.297 |
| FOXJ2(M00423) | CCACAATGGTGCT | 574 | - | 12.95 | 0.00045 | 0.89 |
| FOXO1(M00473) | GGTAAACACG | 1557 | - | 11.68 | 0 | 0 |
| FOXO3(M00477) | CACGTGTTTAC | 1555 | + | 16.56 | 0.000125 | 0.248 |
| FOXO4(M00472) | GTAAACACGT | 1556 | - | 14.99 | 0 | 0 |
| FOXO4(M00476) | ACCTTGCTTACC | 1814 | + | 13.83 | 0.000575 | 1.14 |
| Freac-2(M00290) | GTGAGGTAAACACGTG | 1555 | - | 12.06 | 0 | 0 |
| Freac-3(M00291) | GGTGAGCAAATATAGA | 1298 | + | 7.49 | 0.000675 | 1.34 |
| Freac-4(M00292) | CTTAGGTAAACCCATT | 480 | - | 7.91 | 0.00035 | 0.69 |
| Freac-7(M00293) | GTGAGGTAAACACGTG | 1555 | - | 11.65 | 5.00E-05 | 0.099 |
| GATA-1(M00075) | GCTGATAC | 609 | - | 12.24 | 0.000825 | 1.64 |
| GATA-1(M00127) | GATTATAGG | 1163 | + | 17.96 | 0.000625 | 1.24 |
| GATA-1(M00128) | CCTGATAAACCA | 440 | + | 14.32 | 0.000125 | 0.248 |
| GATA-1(M00346) | CCTGATAAAC | 440 | + | 9.89 | 0.000175 | 0.35 |
| GATA-1(M00347) | CCTGATAAAC | 440 | + | 7.91 | 0.00095 | 1.89 |
| GATA-3(M00077) | CAGATACCA | 347 | - | 7.77 | 0.000925 | 1.84 |
| GATA-3(M00350) | AGAGATCCCA | 1069 | - | 8.41 | 0.000675 | 1.34 |
| GATA-6(M00462) | CCTGATAAAC | 440 | + | 8.28 | 0.000275 | 0.55 |
| GATA-X(M00203) | GATAAACCAT | 443 | + | 11.66 | 0.00065 | 1.29 |
| GC box(M00255) | TATAGGTAGAGTCT | 1432 | - | 10.14 | 5.00E-05 | 0.099 |
| GCNF(M00526) | CCCAAGGTCAAGAACACG | 1649 | + | 14.86 | 0 | 0 |
| GR(M00192) | CTGCAACTCAGTGTCCAAT | 1619 | + | 10.53 | 0.0001 | 0.198 |
| GR(M00205) | CTTTCCTTCTGTCCT | 1744 | - | 11.46 | 0.00075 | 1.49 |
| Hand1:E47(M00222) | CAAGAGGTCTGGGAAT | 824 | - | 10.17 | 7.50E-05 | 0.149 |
| HEN1(M00058) | TTGGCTCTCAGCTGC | 670 | - | 13.6 | 0.000175 | 0.35 |
| HFH-3(M00289) | ACGTGTTTACCTC | 1556 | + | 10.43 | 0.000175 | 0.35 |
| HFH-8(M00294) | ACGTGTTTACCTC | 1556 | + | 7.89 | 0.00085 | 1.69 |
| HLF(M00260) | GTTGGGCCAT | 1872 | - | 9.72 | 0.000375 | 0.75 |
| Hmx3(M00433) | CAAGTGCTGG | 1008 | + | 7.22 | 0.000875 | 1.74 |
| HNF-3beta(M00131) | CTATATTTGCTCA | 1300 | - | 15.4 | 0.00015 | 0.298 |
| Hox-1.3(M00023) | CTAATAATCCATAATTAGTAAGACACATGT | 700 | + | 9.44 | 0.0002 | 0.39 |
| Ik-1(M00086) | CAATGGGAATACG | 1634 | + | 11.08 | 2.50E-05 | 0.05 |
| Ik-2(M00087) | CAATGGGAATAC | 1634 | + | 10.74 | 0.000125 | 0.249 |
| Ik-3(M00088) | TCTTAGGAACACA | 1836 | + | 11.33 | 5.00E-05 | 0.099 |
| IRF-1(M00062) | AGAGTGAAAGC | 1096 | - | 7.99 | 0.00055 | 1.09 |
| IRF-2(M00063) | AGAGTGAAAGC | 1096 | - | 9.47 | 0.00035 | 0.7 |
| ISRE(M00258) | CAGGTTCACATCCCT | 398 | + | 9.57 | 0.00015 | 0.298 |
| Lmo2 complex(M00277) | CACATGTGCAT | 723 | + | 12.74 | 0.00025 | 0.5 |
| Lyf-1(M00141) | CCTGGGAAG | 1923 | - | 8.88 | 0.000475 | 0.95 |
| Max(M00119) | TGCTCACGTGTTTA | 1551 | + | 11.07 | 0 | 0 |
| MEIS1(M00419) | TTCTGACAC | 1532 | + | 15.09 | 0.0004 | 0.8 |
| MRF-2(M00454) | CACAATATTCC | 4 | + | 17.26 | 0.0002 | 0.4 |
| Msx-1(M00394) | CTCTAACTG | 1244 | + | 7.45 | 0.000575 | 1.15 |
| MyoD(M00001) | CATCATGTGCTG | 613 | - | 11.85 | 0 | 0 |
| MyoD(M00184) | TGCACTTGGC | 530 | - | 8.09 | 0.00075 | 1.49 |
| myogenin / NF-1(M00056) | TAGTTGCCTAGGCTGGCTTTCACTCTGTA | 1081 | + | 7.79 | 0.000375 | 0.74 |
| MZF1(M00083) | TCTGGGGA | 1717 | - | 7.71 | 0.000325 | 0.65 |
| MZF1(M00084) | GTGTGAGGGGCTC | 1951 | - | 9.1 | 0.000125 | 0.248 |
| NF-1(M00193) | ATTTGGCCAGCAAAAACC | 636 | + | 9.07 | 0.000475 | 0.94 |
| NF-AT(M00302) | TACAGGAAAGTG | 1388 | + | 7.69 | 0.000975 | 1.94 |
| NF-kappaB(M00194) | TGGGAACTCAACCC | 773 | + | 7.57 | 0.00065 | 1.29 |
| NF-Y(M00185) | TGTCCAATGGG | 1630 | + | 11.78 | 7.50E-05 | 0.149 |
| NF-Y(M00209) | ACTGGTTGGACACA | 85 | + | 8.54 | 0.0005 | 0.99 |
| NF-Y(M00287) | TGTCCAATGGGAAT | 1630 | + | 16.53 | 0 | 0 |
| Nkx2-5(M00240) | TCAAGTA | 1195 | - | 9.02 | 0.000875 | 1.74 |
| NKX6-1(M00424) | CTCTTAGTTGCCT | 1077 | + | 8.32 | 0.000575 | 1.14 |
| N-Myc(M00055) | GCTCACGTGTTT | 1552 | + | 9.98 | 0.000225 | 0.45 |
| NRSF(M00256) | TGTCTCACCAAGAACAGCAGC | 893 | - | 2.29 | 0.00015 | 0.297 |
| Oct-1(M00135) | ATATGCACATGTGTC | 721 | - | 16.3 | 0.000275 | 0.55 |
| Oct-1(M00136) | GAGTATGCATGTG | 107 | - | 16.07 | 0.000175 | 0.35 |
| Oct-1(M00195) | CTATATGTGAAGCCC | 1441 | + | 10.06 | 5.00E-05 | 0.099 |
| Oct-1(M00248) | GCCATGGAAA | 599 | + | 10.25 | 0.000975 | 1.94 |
| OCT-x(M00210) | CACATGTGCATAT | 723 | + | 10.23 | 0.000375 | 0.75 |
| Olf-1(M00261) | TACAGATCCCTGGAGAGGCTAA | 232 | + | 11.03 | 0.000125 | 0.247 |
| p300(M00033) | AGGAGTGGCTC | 1673 | - | 14.16 | 0.00035 | 0.7 |
| p53(M00034) | GGCTAAGTCTGGTCATGCC | 248 | + | 2.46 | 0.000875 | 1.73 |
| p53(M00272) | TGACTTGC | 423 | + | 10.5 | 0.00085 | 1.69 |
| Pax-2(M00098) | ACTAATTATGGATTA | 704 | - | 17.73 | 0.0006 | 1.19 |
| Pax-2(M00486) | GTGAAAGCC | 1095 | - | 7.6 | 0.000225 | 0.45 |
| Pax-3(M00360) | TCCTCACAGTTAG | 1246 | - | 8.73 | 0.0003 | 0.6 |
| Pax-4(M00378) | GAGGTCCACAC | 70 | + | 10.73 | 0.000375 | 0.75 |
| Poly A downstream element(M00211) | AGTGGTCCC | 1474 | + | 11.21 | 5.00E-05 | 0.1 |
| POU3F2(M00463) | ATTCATTTATTTAT | 744 | + | 6.38 | 0.00055 | 1.09 |
| PPAR(M00528) | CTCTAGGTCAGAGCTGA | 1937 | - | 8.59 | 0.000525 | 1.04 |
| PPARalpha:RXR-alpha(M00242) | GGGCTCTAGGTCAGAGCTGA | 1937 | - | 9.96 | 0.00015 | 0.297 |
| PPARG(M00512) | CTCTAGGTCAGAGCTGA | 1937 | - | 7.51 | 0.000625 | 1.24 |
| Retroviral Poly A(M00212) | CATTAAAGCCTTCTCATT | 543 | - | 10.59 | 0.000125 | 0.248 |
| RFX1(M00281) | CCAGCCTAGGCAACTA | 1081 | - | 10.76 | 0.000825 | 1.64 |
| Roaz(M00467) | GCACCCATGGGTAC | 1506 | + | 17.1 | 0 | 0 |
| RORalpha1(M00156) | GGCTCTAGGTCA | 1944 | - | 15.13 | 5.00E-05 | 0.099 |
| RORalpha2(M00157) | GGCTCTAGGTCAG | 1943 | - | 8.75 | 0.000125 | 0.248 |
| RP58(M00532) | TGCACATGTGTC | 721 | - | 9.58 | 0.0001 | 0.199 |
| S8(M00099) | TGGGCCCCATTA | 1340 | - | 15.98 | 0.000525 | 1.04 |
| Sox-5(M00042) | AACAATGG | 299 | + | 15.48 | 0 | 0 |
| SOX-9(M00410) | GGAAAACAATGGCA | 295 | + | 10.49 | 0.000125 | 0.248 |
| Sp1(M00008) | AAGGCAGACA | 1403 | + | 7.89 | 0.000125 | 0.249 |
| Sp1(M00196) | TCTAGGAGTGGCT | 1674 | - | 7.65 | 0.000675 | 1.34 |
| Spz1(M00446) | TCAGCAGGCAAT | 1456 | + | 12.59 | 0.000525 | 1.04 |
| SREBP-1(M00220) | CATCATGTGCT | 614 | - | 10.88 | 0.000225 | 0.45 |
| SREBP-1(M00221) | GGTCATGCCAC | 258 | + | 8.08 | 0.000425 | 0.85 |
| SRF(M00152) | ATGACAAAGTAAGGATTT | 622 | + | 3.44 | 0.000525 | 1.04 |
| SRY(M00148) | AATCTGC | 223 | + | 6.22 | 5.00E-05 | 0.1 |
| SRY(M00160) | GAAAACAATGGC | 296 | + | 9.18 | 0.000125 | 0.249 |
| Staf(M00262) | GTTGCCCTCAGTGGTTTGAGGA | 271 | - | 3.34 | 0.00085 | 1.68 |
| Staf(M00264) | GTTTTCCATGTTGCCCTCA | 283 | - | 16.29 | 0.000175 | 0.35 |
| STAT1(M00224) | CTCCACTTCCC | 1774 | + | 11.62 | 0.000725 | 1.44 |
| STAT1(M00496) | GAGTTCCC | 774 | - | 7.03 | 0.000725 | 1.44 |
| STAT3(M00225) | GGGAAGTGGAG | 1774 | - | 11.22 | 0.0004 | 0.79 |
| STAT3(M00497) | GAGTTCCA | 944 | - | 7.59 | 0.0002 | 0.4 |
| STAT5A (homodimer)(M00457) | TGTCCTGGAACTC | 939 | + | 13.71 | 0.0003 | 0.6 |
| STAT5A (homotetramer)(M00460) | TTCAGAGTAGGTTTGCTCGTGGGG | 1480 | - | 8.66 | 0.00025 | 0.49 |
| STAT5A(M00499) | GAGTTCCA | 944 | - | 8.94 | 0 | 0 |
| STAT5B (homodimer)(M00459) | TGTCCTGGAACTC | 939 | + | 14.72 | 0.0001 | 0.199 |
| STATx(M00223) | TTCCCAGCA | 770 | - | 9.59 | 0.000225 | 0.45 |
| TATA(M00216) | CCTTTAAAAA | 1050 | - | 9.52 | 0.000275 | 0.55 |
| TATA(M00252) | CTTTAAAAAGGGTCG | 1044 | - | 10.54 | 0.000175 | 0.35 |
| Tax/CREB(M00114) | ATGGGTTTACCTAAG | 481 | + | 7.99 | 0.000925 | 1.84 |
| Tax/CREB(M00115) | ATGGCACATCCCCTA | 303 | + | 8.61 | 0.000275 | 0.55 |
| TBP(M00471) | GGTAAACC | 484 | - | 8.32 | 0 | 0 |
| TCF11(M00285) | GTCATTCTAG | 1278 | - | 16.62 | 0 | 0 |
| TCF11:MafG(M00284) | ACATGATGACAAAGTAAGGAT | 617 | + | 12.79 | 0.000375 | 0.74 |
| TGIF(M00418) | TGTCAGAA | 1532 | - | 16.15 | 0.000125 | 0.249 |
| USF(M00121) | TCACGTGTTTA | 1554 | + | 15.22 | 0.000325 | 0.65 |
| USF(M00122) | TCACGTGTTTA | 1554 | + | 15.41 | 0 | 0 |
| USF(M00187) | CTCACGTGTT | 1553 | + | 11.1 | 7.50E-05 | 0.149 |
| USF(M00217) | CACGTGA | 1554 | - | 12.69 | 0.000125 | 0.249 |
| v-ErbA(M00239) | AAGTCTGGTCATGCCA | 252 | + | 8.63 | 0.00065 | 1.29 |
| v-Maf(M00035) | ACATGATGACAAAGTAAGG | 617 | + | 11.42 | 7.50E-05 | 0.149 |
| v-Myb(M00227) | CTAACTGT | 1246 | + | 13.09 | 0.00035 | 0.7 |
| XBP-1(M00251) | GTAAACACGTGAGCAGT | 1549 | - | 7.29 | 0.001 | 1.98 |
| XFD-1(M00267) | CATGTGCATATAGC | 725 | + | 7.99 | 0.000875 | 1.74 |
| Xvent-1(M00445) | AGCAGATTTGT | 220 | - | 14.01 | 0.0005 | 0.99 |
| YY1(M00059) | GAGCACCATTGTGGCAA | 573 | + | 10.7 | 5.00E-05 | 0.099 |
| YY1(M00069) | ATGCCACCATCCTCAAACCA | 262 | + | 9.35 | 0.00035 | 0.69 |
| Zic2(M00449) | ACAGTGGTC | 1472 | + | 6.71 | 0.000525 | 1.05 |
| Zic3(M00450) | TGGGAACTC | 773 | + | 7.26 | 0.000375 | 0.75 |
| ZID(M00085) | GGGCTCTAGGTCA | 1944 | - | 11.25 | 7.50E-05 | 0.149 |

**Supplementary Data 4. The predicted the potential targets of miR-135a-5p by Targetscan.**

| Target gene | Representative transcript | Cumulative weighted context++ score | Total context++ score | Aggregate PCT |
| --- | --- | --- | --- | --- |
| Syt2 | ENSMUST00000121990.1 | -0.86 | -0.86 | > 0.99 |
| Strbp | ENSMUST00000028279.4 | -0.12 | -0.59 | > 0.99 |
| Cplx2 | ENSMUST00000026985.8 | -0.87 | -0.94 | > 0.99 |
| Cplx1 | ENSMUST00000046892.9 | -1.27 | -1.27 | > 0.99 |
| Zfp652 | ENSMUST00000107717.2 | -0.13 | -0.47 | 0.99 |
| Ergic2 | ENSMUST00000136008.2 | -0.4 | -0.69 | 0.98 |
| Kcnb1 | ENSMUST00000059826.8 | -0.38 | -0.85 | 0.98 |
| Foxn3 | ENSMUST00000046859.5 | -0.9 | -0.91 | 0.98 |
| Nr3c2 | ENSMUST00000109913.3 | -0.68 | -0.7 | 0.98 |
| Slc8a1 | ENSMUST00000163680.3 | -0.16 | -0.64 | 0.97 |
| Kcnn3 | ENSMUST00000000811.7 | -0.19 | -0.7 | 0.97 |
| Rbmx | ENSMUST00000098470.3 | -0.57 | -0.77 | 0.97 |
| Tnpo1 | ENSMUST00000109399.3 | -0.59 | -0.61 | 0.96 |
| Slc6a5 | ENSMUST00000056442.6 | -0.31 | -0.31 | 0.95 |
| Esrra | ENSMUST00000025906.5 | -0.55 | -0.55 | 0.94 |
| Cacna1e | ENSMUST00000187541.1 | -0.48 | -0.48 | 0.94 |
| Frmpd4 | ENSMUST00000112149.2 | -0.22 | -0.22 | 0.94 |
| Vldlr | ENSMUST00000167487.2 | -1 | -1 | 0.94 |
| Trpc6 | ENSMUST00000050433.6 | -0.66 | -0.82 | 0.94 |
| Cramp1l | ENSMUST00000073337.6 | -0.32 | -0.32 | 0.94 |
| Bahcc1 | ENSMUST00000044985.8 | -0.22 | -0.22 | 0.94 |
| Hcn1 | ENSMUST00000006991.7 | -0.13 | -0.41 | 0.94 |
| Faxc | ENSMUST00000029908.7 | -0.07 | -0.27 | 0.94 |
| Mtmr12 | ENSMUST00000038172.9 | -1.7 | -1.82 | 0.94 |
| Ppp6r3 | ENSMUST00000113997.3 | -0.37 | -0.39 | 0.94 |
| Cacna1d | ENSMUST00000112249.2 | -0.23 | -0.23 | 0.94 |
| Mob1b | ENSMUST00000113229.2 | -0.5 | -0.51 | 0.93 |
| Ebf1 | ENSMUST00000081265.6 | -0.44 | -0.89 | 0.93 |
| Phldb2 | ENSMUST00000036355.7 | -0.33 | -0.42 | 0.93 |
| Zbtb46 | ENSMUST00000029106.7 | -0.3 | -0.32 | 0.93 |
| Glyctk | ENSMUST00000036382.7 | -0.43 | -0.48 | 0.93 |
| Man1a | ENSMUST00000003843.9 | -0.49 | -0.5 | 0.93 |
| Ubox5 | ENSMUST00000028761.4 | -0.58 | -0.63 | 0.93 |
| Arhgef2 | ENSMUST00000176500.2 | -0.33 | -0.35 | 0.93 |
| Dyrk1b | ENSMUST00000085901.7 | -0.24 | -0.27 | 0.93 |
| Acvr1b | ENSMUST00000000544.10 | -0.54 | -0.54 | 0.93 |
| Usp13 | ENSMUST00000072312.6 | -0.35 | -0.35 | 0.93 |
| Rock2 | ENSMUST00000020904.6 | -0.34 | -0.35 | 0.93 |
| Zranb2 | ENSMUST00000106057.2 | -0.52 | -0.55 | 0.93 |
| Elk3 | ENSMUST00000008542.6 | -0.51 | -0.58 | 0.93 |
| Srsf3 | ENSMUST00000130216.1 | -0.32 | -0.84 | 0.93 |
| Jade1 | ENSMUST00000026865.9 | -0.18 | -0.34 | 0.93 |
| Slco5a1 | ENSMUST00000188454.1 | -0.33 | -0.33 | 0.93 |
| Setd7 | ENSMUST00000037141.7 | -0.23 | -0.39 | 0.93 |
| Arhgef4 | ENSMUST00000047664.10 | -0.28 | -0.28 | 0.93 |
| Rorb | ENSMUST00000040153.9 | -0.27 | -0.27 | 0.93 |
| Ghsr | ENSMUST00000057186.1 | -0.32 | -0.32 | 0.93 |
| Rsbn1l | ENSMUST00000036489.5 | -0.25 | -0.5 | 0.93 |
| Kdm7a | ENSMUST00000002305.8 | -0.36 | -0.39 | 0.93 |
| Cdyl2 | ENSMUST00000109102.2 | -0.11 | -0.23 | 0.93 |
| Atg14 | ENSMUST00000042988.6 | -0.38 | -0.43 | 0.93 |
| Ndufb9 | ENSMUST00000022980.3 | 0 | -0.44 | 0.93 |
| Ubfd1 | ENSMUST00000033158.4 | -0.22 | -0.29 | 0.93 |
| Slc24a2 | ENSMUST00000107157.3 | -0.61 | -0.61 | 0.93 |
| Fkbp1a | ENSMUST00000044011.6 | -0.56 | -0.89 | 0.93 |
| C030046E11Rik | ENSMUST00000043610.7 | -0.13 | -0.32 | 0.93 |
| Smim13 | ENSMUST00000165561.2 | -0.77 | -0.82 | 0.93 |
| Wscd2 | ENSMUST00000094452.3 | -0.39 | -0.41 | 0.93 |
| 1600012H06Rik | ENSMUST00000052691.8 | -0.68 | -0.69 | 0.92 |
| Slc30a4 | ENSMUST00000005952.5 | -0.35 | -0.4 | 0.92 |
| Stk35 | ENSMUST00000166282.2 | -0.08 | -0.48 | 0.92 |
| Ext1 | ENSMUST00000077273.3 | -0.02 | -0.31 | 0.92 |
| Rasal2 | ENSMUST00000078308.7 | -0.37 | -0.41 | 0.92 |
| Pde1a | ENSMUST00000102655.4 | -0.5 | -0.5 | 0.92 |
| Ywhag | ENSMUST00000055808.5 | -0.28 | -0.29 | 0.92 |
| Ccsap | ENSMUST00000034452.6 | -0.41 | -0.48 | 0.92 |
| Zfp385b | ENSMUST00000111830.3 | -0.53 | -0.53 | 0.92 |
| Rock1 | ENSMUST00000067947.5 | -0.3 | -0.3 | 0.92 |
| Thrb | ENSMUST00000091471.5 | -0.4 | -0.42 | 0.92 |
| Sdk1 | ENSMUST00000085774.5 | -0.16 | -1.03 | 0.91 |
| Mturn | ENSMUST00000190641.1 | -0.95 | -1.1 | 0.91 |
| Camk1g | ENSMUST00000016323.5 | -0.49 | -0.5 | 0.91 |
| Fcho2 | ENSMUST00000099277.5 | -0.35 | -0.42 | 0.91 |
| Ntng1 | ENSMUST00000156177.3 | -0.44 | -0.44 | 0.91 |
| Caln1 | ENSMUST00000111288.2 | -0.05 | -0.37 | 0.91 |
| Parn | ENSMUST00000058884.8 | -0.32 | -0.32 | 0.91 |
| Clcn3 | ENSMUST00000093490.3 | -0.29 | -0.34 | 0.91 |
| Slc39a13 | ENSMUST00000073575.6 | -0.22 | -0.43 | 0.91 |
| Hmgxb3 | ENSMUST00000091884.4 | -0.37 | -0.37 | 0.9 |
| Kdm5b | ENSMUST00000112198.2 | -0.02 | -0.26 | 0.9 |
| Prune2 | ENSMUST00000087689.4 | -0.12 | -0.13 | 0.9 |
| Bsn | ENSMUST00000035208.8 | -0.15 | -0.15 | 0.9 |
| Kcnd1 | ENSMUST00000009875.4 | -0.37 | -0.37 | 0.9 |
| Scn2a1 | ENSMUST00000028377.8 | -0.13 | -0.13 | 0.9 |
| Mapre2 | ENSMUST00000170802.2 | -0.38 | -0.45 | 0.9 |
| Ddx3x | ENSMUST00000000804.6 | -0.34 | -0.34 | 0.9 |
| Chmp4b | ENSMUST00000044277.9 | -0.36 | -0.61 | 0.9 |
| Pcyt1b | ENSMUST00000113933.3 | -0.25 | -0.39 | 0.9 |
| Klf16 | ENSMUST00000038558.8 | -0.38 | -0.43 | 0.9 |
| Zbtb34 | ENSMUST00000113158.2 | -0.2 | -0.2 | 0.9 |
| Ppargc1b | ENSMUST00000075299.7 | -0.27 | -0.29 | 0.89 |
| Entpd7 | ENSMUST00000081079.5 | -1.39 | -1.96 | 0.89 |
| Net1 | ENSMUST00000091853.5 | -0.32 | -0.34 | 0.89 |
| Dlgap2 | ENSMUST00000133298.2 | 0 | -0.26 | 0.89 |
| Suv420h2 | ENSMUST00000098853.3 | -0.38 | -0.38 | 0.89 |
| Cttnbp2 | ENSMUST00000090601.6 | -1.24 | -1.24 | 0.89 |
| Peli2 | ENSMUST00000073150.4 | -0.09 | -0.52 | 0.89 |
| Taf4a | ENSMUST00000041618.8 | -0.32 | -0.32 | 0.89 |
| Lzts1 | ENSMUST00000185176.2 | -0.63 | -0.63 | 0.88 |
| Mef2c | ENSMUST00000163888.2 | -0.53 | -0.54 | 0.88 |
| Mtss1 | ENSMUST00000080371.6 | -0.25 | -0.32 | 0.88 |
| Bach1 | ENSMUST00000026703.5 | -0.42 | -0.47 | 0.88 |
| Grin2b | ENSMUST00000053880.7 | -1.06 | -1.06 | 0.88 |
| Msl2 | ENSMUST00000085177.4 | -0.12 | -0.2 | 0.88 |
| Vcan | ENSMUST00000109546.3 | -0.24 | -0.25 | 0.87 |
| Ago2 | ENSMUST00000044113.10 | -0.31 | -0.4 | 0.87 |
| Stat6 | ENSMUST00000092074.6 | -0.18 | -0.48 | 0.87 |
| Ssr2 | ENSMUST00000035785.7 | -0.51 | -0.55 | 0.87 |
| Rspo2 | ENSMUST00000063492.6 | -0.54 | -0.54 | 0.86 |
| Zdhhc18 | ENSMUST00000084238.4 | -0.16 | -0.36 | 0.86 |
| Anxa7 | ENSMUST00000065504.10 | -0.67 | -0.67 | 0.86 |
| Shisa7 | ENSMUST00000172377.2 | -0.27 | -0.49 | 0.86 |
| Sp3 | ENSMUST00000102689.4 | -0.5 | -0.52 | 0.85 |
| Evi5 | ENSMUST00000112642.2 | -0.28 | -0.4 | 0.85 |
| Med13 | ENSMUST00000043624.8 | -0.41 | -0.66 | 0.85 |
| Wac | ENSMUST00000167020.2 | -0.11 | -0.11 | 0.85 |
| Asph | ENSMUST00000108333.2 | -0.72 | -0.8 | 0.85 |
| Ado | ENSMUST00000075686.4 | -0.51 | -0.52 | 0.84 |
| Ptprd | ENSMUST00000107289.3 | -0.16 | -0.18 | 0.84 |
| Zfp518a | ENSMUST00000050092.6 | -0.34 | -0.34 | 0.83 |
| Smad5 | ENSMUST00000069557.8 | -0.28 | -0.29 | 0.83 |
| Pik3r2 | ENSMUST00000034296.9 | -0.41 | -0.41 | 0.83 |
| Erbb4 | ENSMUST00000119142.2 | -0.14 | -0.16 | 0.83 |
| Gabrb2 | ENSMUST00000007797.4 | -0.12 | -0.32 | 0.83 |
| Gsk3b | ENSMUST00000023507.7 | -0.24 | -0.27 | 0.83 |
| Ddi2 | ENSMUST00000102484.4 | -0.26 | -0.3 | 0.83 |
| Rsc1a1 | ENSMUST00000105782.1 | -0.21 | -0.34 | 0.83 |
| Cadm3 | ENSMUST00000111220.2 | -0.1 | -0.29 | 0.83 |
| Foxo1 | ENSMUST00000053764.5 | -0.24 | -0.43 | 0.83 |
| Bace1 | ENSMUST00000034591.5 | -0.2 | -0.23 | 0.82 |
| Col5a1 | ENSMUST00000028280.8 | -0.07 | -0.26 | 0.82 |
| Lancl3 | ENSMUST00000069763.2 | -0.13 | -0.28 | 0.82 |
| Wapal | ENSMUST00000048263.8 | -0.25 | -0.35 | 0.82 |
| E130309D14Rik | ENSMUST00000100866.2 | -0.33 | -0.38 | 0.82 |
| Mphosph9 | ENSMUST00000031344.7 | -0.03 | -0.18 | 0.81 |
| Aebp2 | ENSMUST00000032359.9 | -0.27 | -0.27 | 0.81 |
| Gabrb1 | ENSMUST00000031122.7 | -0.36 | -0.73 | 0.81 |
| Syt3 | ENSMUST00000118831.2 | -0.4 | -0.4 | 0.81 |
| Zfp131 | ENSMUST00000177916.2 | -0.49 | -0.49 | 0.81 |
| Cpd | ENSMUST00000021201.5 | -0.09 | -0.1 | 0.8 |
| Rbak | ENSMUST00000165318.2 | -0.45 | -0.45 | 0.8 |
| Lmtk2 | ENSMUST00000041804.7 | -1.26 | -1.28 | 0.8 |
| Kcnab3 | ENSMUST00000018614.2 | -0.66 | -0.66 | 0.8 |
| Tcf7l2 | ENSMUST00000111656.2 | -0.43 | -0.47 | 0.79 |
| Fbxo28 | ENSMUST00000051431.4 | -0.07 | -0.76 | 0.79 |
| Nckipsd | ENSMUST00000035218.7 | -0.35 | -0.4 | 0.79 |
| Nxph1 | ENSMUST00000160300.1 | -0.01 | -0.37 | 0.79 |
| Fgf11 | ENSMUST00000102585.1 | -0.81 | -0.83 | 0.79 |
| Lats2 | ENSMUST00000022531.8 | -0.31 | -0.33 | 0.79 |
| Atp2b4 | ENSMUST00000048953.8 | -0.19 | -0.19 | 0.78 |
| Kcnq5 | ENSMUST00000029667.7 | -0.3 | -0.31 | 0.78 |
| Adcy1 | ENSMUST00000020706.4 | -0.73 | -1.24 | 0.77 |
| Zswim4 | ENSMUST00000039480.5 | -0.23 | -0.26 | 0.77 |
| Rgl1 | ENSMUST00000111859.2 | -0.35 | -0.43 | 0.77 |
| Skor1 | ENSMUST00000119146.2 | -0.34 | -0.34 | 0.77 |
| Tet3 | ENSMUST00000089622.5 | -1.24 | -1.25 | 0.77 |
| Hoxa10 | ENSMUST00000125581.1 | -0.41 | -0.41 | 0.76 |
| Elovl6 | ENSMUST00000071402.2 | -0.32 | -0.35 | 0.75 |
| Kcns3 | ENSMUST00000164495.2 | -0.26 | -0.26 | 0.75 |
| Dnajc16 | ENSMUST00000038014.5 | -0.24 | -0.25 | 0.75 |
| Cblb | ENSMUST00000114471.1 | -0.2 | -0.2 | 0.75 |
| Clasp1 | ENSMUST00000188710.1 | -0.2 | -0.23 | 0.75 |
| Wdr20 | ENSMUST00000095410.2 | -0.19 | -0.19 | 0.75 |
| Npat | ENSMUST00000035850.7 | -0.27 | -0.4 | 0.75 |
| Csmd1 | ENSMUST00000082104.6 | -0.19 | -0.19 | 0.75 |
| Btaf1 | ENSMUST00000099494.3 | -0.18 | -0.21 | 0.75 |
| Lmx1b | ENSMUST00000041730.5 | -0.15 | -0.15 | 0.75 |
| Ptprf | ENSMUST00000049074.7 | -0.1 | -0.13 | 0.75 |
| Hif1an | ENSMUST00000040455.4 | -1.48 | -1.63 | 0.75 |
| Dcun1d4 | ENSMUST00000063882.6 | -0.27 | -0.27 | 0.75 |
| Mb21d2 | ENSMUST00000100023.1 | -0.18 | -0.2 | 0.75 |
| Traf4 | ENSMUST00000017530.3 | -0.14 | -0.15 | 0.75 |
| Prrc2c | ENSMUST00000182149.2 | -0.17 | -0.18 | 0.75 |
| Dcp2 | ENSMUST00000025350.8 | -0.06 | -0.29 | 0.75 |
| Foxn2 | ENSMUST00000112238.3 | -0.44 | -0.45 | 0.75 |
| Ssr1 | ENSMUST00000021864.6 | -0.33 | -0.4 | 0.75 |
| Robo1 | ENSMUST00000023600.7 | -0.22 | -0.23 | 0.75 |
| Exoc5 | ENSMUST00000162175.2 | -0.2 | -0.2 | 0.75 |
| Lypd1 | ENSMUST00000159417.1 | -0.13 | -0.17 | 0.75 |
| Lonrf1 | ENSMUST00000065297.5 | -0.33 | -0.38 | 0.75 |
| Naa15 | ENSMUST00000029303.7 | -0.02 | -0.09 | 0.75 |
| Mfhas1 | ENSMUST00000037666.5 | -0.09 | -0.1 | 0.75 |
| Paf1 | ENSMUST00000003529.8 | 0 | -0.17 | 0.75 |
| C2cd2 | ENSMUST00000170757.1 | -0.32 | -0.33 | 0.75 |
| Ccdc50 | ENSMUST00000100026.4 | -0.09 | -0.1 | 0.75 |
| Tgfbr2 | ENSMUST00000061101.5 | -0.18 | -0.18 | 0.75 |
| Cxcl12 | ENSMUST00000112866.2 | -0.3 | -0.3 | 0.75 |
| Usp31 | ENSMUST00000046929.6 | -0.16 | -0.16 | 0.75 |
| Slc16a6 | ENSMUST00000070152.6 | -0.06 | -0.17 | 0.75 |
| Atad1 | ENSMUST00000070210.4 | -0.27 | -0.72 | 0.75 |
| Hic2 | ENSMUST00000090190.6 | -0.18 | -0.2 | 0.75 |
| Irs2 | ENSMUST00000040514.6 | -0.14 | -0.19 | 0.75 |
| Vamp2 | ENSMUST00000117780.1 | -0.32 | -0.32 | 0.74 |
| Mbnl1 | ENSMUST00000099087.2 | -0.23 | -0.29 | 0.74 |
| Rora | ENSMUST00000034766.8 | -0.16 | -0.18 | 0.74 |
| Ago3 | ENSMUST00000069097.7 | -0.21 | -0.22 | 0.74 |
| Luc7l3 | ENSMUST00000107820.1 | -0.16 | -0.29 | 0.74 |
| Trp53inp1 | ENSMUST00000029865.3 | -0.13 | -0.14 | 0.74 |
| Efnb2 | ENSMUST00000001319.8 | -0.25 | -0.26 | 0.74 |
| Syt1 | ENSMUST00000105276.2 | -0.28 | -0.28 | 0.74 |
| Ildr2 | ENSMUST00000111416.1 | -0.32 | -0.34 | 0.74 |
| Tfap2a | ENSMUST00000110193.3 | -0.25 | -0.26 | 0.74 |
| Smarce1 | ENSMUST00000103133.3 | -0.02 | -0.31 | 0.74 |
| Pik3cd | ENSMUST00000038859.8 | -0.23 | -0.25 | 0.74 |
| Dpf1 | ENSMUST00000049977.7 | -0.29 | -0.29 | 0.74 |
| Sesn3 | ENSMUST00000034507.7 | -0.12 | -0.12 | 0.73 |
| Nrg1 | ENSMUST00000073884.4 | 0 | -0.19 | 0.73 |
| Sv2b | ENSMUST00000165175.2 | -0.24 | -0.24 | 0.73 |
| Myo9a | ENSMUST00000136740.2 | -0.23 | -0.26 | 0.73 |
| Edem3 | ENSMUST00000059498.6 | -0.31 | -0.32 | 0.72 |
| Rps6kb1 | ENSMUST00000154617.2 | -0.16 | -0.26 | 0.72 |
| Fam78b | ENSMUST00000165874.2 | -0.23 | -0.52 | 0.72 |
| Erp44 | ENSMUST00000030028.4 | -0.02 | -0.28 | 0.72 |
| Kcnk12 | ENSMUST00000055221.7 | -0.18 | -0.43 | 0.72 |
| Bbx | ENSMUST00000138166.2 | -0.4 | -0.53 | 0.71 |
| Mras | ENSMUST00000035045.9 | -0.29 | -0.44 | 0.71 |
| Zkscan1 | ENSMUST00000019660.5 | -0.23 | -0.36 | 0.7 |
| Igsf10 | ENSMUST00000039419.6 | -0.17 | -0.17 | 0.7 |
| Rybp | ENSMUST00000101118.2 | -0.05 | -0.15 | 0.7 |
| Lrp11 | ENSMUST00000130590.2 | -0.19 | -0.2 | 0.7 |
| Grik3 | ENSMUST00000030676.7 | -0.35 | -0.36 | 0.7 |
| Gucy1a2 | ENSMUST00000115733.1 | -0.21 | -0.27 | 0.7 |
| Pip4k2c | ENSMUST00000013970.7 | -0.18 | -0.19 | 0.69 |
| Zfp292 | ENSMUST00000098163.3 | -0.47 | -0.81 | 0.69 |
| Ppp1cc | ENSMUST00000102528.5 | -0.39 | -0.42 | 0.69 |
| Zdhhc6 | ENSMUST00000076891.5 | -0.31 | -0.31 | 0.69 |
| Entpd4 | ENSMUST00000184973.2 | -0.26 | -0.26 | 0.69 |
| Nup153 | ENSMUST00000021803.9 | -0.26 | -0.29 | 0.69 |
| Grk5 | ENSMUST00000003313.8 | -0.23 | -0.46 | 0.69 |
| Pggt1b | ENSMUST00000025354.3 | -0.22 | -0.22 | 0.69 |
| Mtdh | ENSMUST00000022865.10 | -0.21 | -0.31 | 0.69 |
| Siah1a | ENSMUST00000045296.4 | -0.21 | -0.24 | 0.69 |
| Kctd1 | ENSMUST00000168989.2 | -0.2 | -0.44 | 0.69 |
| Calml4 | ENSMUST00000034777.7 | -0.17 | -0.56 | 0.69 |
| Gm21685 | ENSMUST00000185072.2 | -0.29 | -0.29 | 0.69 |
| Tmem189 | ENSMUST00000006587.6 | -0.28 | -0.28 | 0.69 |
| D17Wsu92e | ENSMUST00000114863.3 | -0.34 | -0.37 | 0.69 |
| Josd1 | ENSMUST00000023061.5 | -0.4 | -0.4 | 0.69 |
| Chst11 | ENSMUST00000040110.7 | -0.13 | -0.26 | 0.69 |
| Klf4 | ENSMUST00000107619.2 | -0.36 | -0.36 | 0.69 |
| Trpm7 | ENSMUST00000103224.4 | -0.25 | -0.33 | 0.69 |
| Ptbp2 | ENSMUST00000029780.7 | -0.26 | -0.26 | 0.69 |
| Arhgef6 | ENSMUST00000033468.5 | -0.28 | -0.29 | 0.69 |
| Cux2 | ENSMUST00000111752.4 | -0.15 | -0.18 | 0.69 |
| Ptk2 | ENSMUST00000110036.3 | -0.27 | -0.4 | 0.69 |
| Slc5a7 | ENSMUST00000095712.3 | -1.11 | -1.11 | 0.69 |
| Znrf3 | ENSMUST00000172492.2 | -0.11 | -0.25 | 0.69 |
| Trpc1 | ENSMUST00000189137.1 | -0.36 | -0.43 | 0.69 |
| Ralbp1 | ENSMUST00000166543.3 | -0.09 | -0.26 | 0.69 |
| Arel1 | ENSMUST00000043169.8 | -0.28 | -0.36 | 0.69 |
| G3bp1 | ENSMUST00000018727.3 | -0.09 | -0.15 | 0.69 |
| Cep170 | ENSMUST00000057037.7 | -0.2 | -0.29 | 0.69 |
| Uri1 | ENSMUST00000085513.4 | -0.19 | -0.28 | 0.69 |
| Sdcbp | ENSMUST00000029912.5 | -0.37 | -0.37 | 0.69 |
| Dag1 | ENSMUST00000166905.2 | -0.23 | -0.23 | 0.69 |
| Psmb4 | ENSMUST00000005923.6 | 0 | -0.17 | 0.69 |
| Ppp1r12c | ENSMUST00000013886.8 | -0.35 | -0.36 | 0.69 |
| Nudt4 | ENSMUST00000020217.5 | -0.44 | -0.46 | 0.68 |
| Gja1 | ENSMUST00000068581.7 | -0.18 | -0.18 | 0.68 |
| Myo1c | ENSMUST00000102505.4 | -0.3 | -0.3 | 0.68 |
| Shisa6 | ENSMUST00000066679.6 | -0.12 | -0.12 | 0.68 |
| Clvs2 | ENSMUST00000019920.7 | -0.48 | -0.48 | 0.68 |
| Ralgps2 | ENSMUST00000172057.2 | -0.13 | -0.34 | 0.68 |
| Osbpl8 | ENSMUST00000105275.2 | -0.07 | -0.09 | 0.68 |
| Ppp1r9b | ENSMUST00000038696.6 | -0.28 | -0.51 | 0.68 |
| Sntb2 | ENSMUST00000047425.3 | -0.1 | -0.23 | 0.68 |
| Mat2a | ENSMUST00000059472.8 | -0.3 | -0.43 | 0.68 |
| Ggnbp2 | ENSMUST00000108081.3 | -0.13 | -0.36 | 0.68 |
| St3gal6 | ENSMUST00000137035.2 | 0 | -0.27 | 0.68 |
| Nsg1 | ENSMUST00000031009.4 | -0.21 | -0.22 | 0.68 |
| Ccnt2 | ENSMUST00000112570.1 | -0.06 | -0.29 | 0.68 |
| Dirc2 | ENSMUST00000023554.8 | -0.41 | -0.5 | 0.68 |
| Ndufa4 | ENSMUST00000031637.5 | -0.43 | -0.43 | 0.68 |
| Fam81a | ENSMUST00000034749.9 | -0.13 | -0.14 | 0.68 |
| Tbk1 | ENSMUST00000020316.2 | -0.64 | -1.54 | 0.67 |
| Extl2 | ENSMUST00000029575.6 | -0.33 | -0.34 | 0.67 |
| Hipk3 | ENSMUST00000028600.8 | -0.21 | -0.23 | 0.67 |
| Rnf152 | ENSMUST00000058688.6 | -0.44 | -0.46 | 0.67 |
| Phospho1 | ENSMUST00000054173.3 | -0.35 | -0.35 | 0.67 |
| Mef2a | ENSMUST00000156690.2 | -0.33 | -0.34 | 0.67 |
| Adcyap1 | ENSMUST00000064775.6 | -0.47 | -0.47 | 0.67 |
| Frk | ENSMUST00000019913.8 | -0.27 | -0.28 | 0.67 |
| Nucks1 | ENSMUST00000062264.7 | -0.15 | -0.27 | 0.67 |
| Sec62 | ENSMUST00000029256.7 | -0.37 | -0.77 | 0.67 |
| Elk4 | ENSMUST00000086556.6 | -0.51 | -0.83 | 0.67 |
| Sgms1 | ENSMUST00000099514.4 | -0.25 | -0.35 | 0.67 |
| Elovl2 | ENSMUST00000021793.8 | -0.59 | -0.6 | 0.67 |
| Ywhae | ENSMUST00000067664.4 | -0.37 | -0.38 | 0.67 |
| Rcor1 | ENSMUST00000084968.8 | -0.27 | -0.35 | 0.67 |
| Rims1 | ENSMUST00000115273.3 | -0.23 | -0.23 | 0.67 |
| D10Bwg1379e | ENSMUST00000019999.5 | -0.18 | -0.23 | 0.67 |
| Atp1b1 | ENSMUST00000027863.7 | -0.37 | -0.37 | 0.67 |
| Jakmip2 | ENSMUST00000082254.6 | -0.31 | -0.31 | 0.67 |
| Prkd3 | ENSMUST00000003191.8 | -0.16 | -0.16 | 0.67 |
| Slc44a5 | ENSMUST00000089948.5 | -0.14 | -0.14 | 0.67 |
| Dgkh | ENSMUST00000074729.5 | -0.08 | -0.2 | 0.67 |
| Dram2 | ENSMUST00000067630.7 | -0.38 | -0.57 | 0.67 |
| BC030336 | ENSMUST00000060175.6 | -0.2 | -0.34 | 0.67 |
| Klhl28 | ENSMUST00000021331.7 | -0.3 | -0.38 | 0.67 |
| Lmln | ENSMUST00000023497.2 | -0.16 | -0.16 | 0.67 |
| Sorl1 | ENSMUST00000060989.8 | -0.08 | -0.1 | 0.67 |
| Zfp704 | ENSMUST00000041124.7 | -0.06 | -0.15 | 0.67 |
| Stambp | ENSMUST00000068054.7 | -0.27 | -0.27 | 0.66 |
| Gga1 | ENSMUST00000041587.7 | -0.2 | -0.2 | 0.66 |
| Ndrg4 | ENSMUST00000073139.8 | -0.3 | -0.3 | 0.66 |
| Rims2 | ENSMUST00000082054.6 | -0.67 | -1.06 | 0.66 |
| B4galt5 | ENSMUST00000109221.3 | -0.27 | -0.27 | 0.66 |
| Ptchd4 | ENSMUST00000048691.4 | -0.12 | -0.51 | 0.66 |
| Syn3 | ENSMUST00000120638.2 | -0.24 | -0.42 | 0.66 |
| Raver2 | ENSMUST00000038463.9 | -0.45 | -0.45 | 0.66 |
| Ppp2r5c | ENSMUST00000084985.4 | -0.26 | -0.26 | 0.66 |
| Cenpb | ENSMUST00000089510.4 | -0.26 | -0.39 | 0.66 |
| Csnk1a1 | ENSMUST00000165123.2 | -0.08 | -0.74 | 0.66 |
| Bmpr1a | ENSMUST00000165280.2 | -0.56 | -0.66 | 0.65 |
| Qk | ENSMUST00000097414.4 | -0.24 | -0.25 | 0.65 |
| Phf20 | ENSMUST00000037401.8 | -0.18 | -0.2 | 0.65 |
| Rnf217 | ENSMUST00000081989.7 | -0.05 | -0.37 | 0.65 |
| Dlg2 | ENSMUST00000107196.3 | -0.08 | -0.11 | 0.65 |
| Scn2b | ENSMUST00000170998.2 | -0.29 | -0.29 | 0.65 |
| Pde7a | ENSMUST00000099195.4 | -0.13 | -0.21 | 0.65 |
| Gata3 | ENSMUST00000102976.3 | -0.33 | -0.33 | 0.65 |
| Ptpn9 | ENSMUST00000034832.6 | -0.18 | -0.18 | 0.65 |
| Mapk10 | ENSMUST00000112848.2 | -0.03 | -0.16 | 0.65 |
| Stk38l | ENSMUST00000001675.8 | -0.23 | -0.23 | 0.65 |
| St6gal2 | ENSMUST00000025000.3 | -0.17 | -0.17 | 0.65 |
| Zfp654 | ENSMUST00000052588.4 | -0.2 | -0.4 | 0.65 |
| Onecut2 | ENSMUST00000175965.3 | -0.38 | -0.38 | 0.65 |
| Kat6b | ENSMUST00000069648.8 | -0.22 | -0.24 | 0.65 |
| Sp1 | ENSMUST00000001326.6 | -0.19 | -0.19 | 0.65 |
| Spata2 | ENSMUST00000057627.10 | -0.11 | -0.14 | 0.64 |
| Pou2f3 | ENSMUST00000034513.7 | -0.22 | -0.25 | 0.64 |
| Gramd4 | ENSMUST00000138134.2 | -0.1 | -0.1 | 0.64 |
| Zbtb44 | ENSMUST00000115222.3 | -0.38 | -0.5 | 0.64 |
| Prdm16 | ENSMUST00000070313.8 | -0.11 | -0.23 | 0.64 |
| Ccdc171 | ENSMUST00000053414.7 | -0.23 | -0.3 | 0.64 |
| Tbx4 | ENSMUST00000000096.6 | -0.34 | -0.35 | 0.64 |
| Hif1a | ENSMUST00000110461.2 | -0.58 | -0.58 | 0.64 |
| Sema3a | ENSMUST00000030714.7 | -0.26 | -0.33 | 0.64 |
| Fermt2 | ENSMUST00000045905.7 | -0.41 | -0.48 | 0.64 |
| Gria3 | ENSMUST00000076349.6 | -0.27 | -0.27 | 0.63 |
| Lrrn1 | ENSMUST00000049285.8 | -0.17 | -0.17 | 0.63 |
| Etv3 | ENSMUST00000119109.2 | -0.17 | -0.17 | 0.63 |
| Trim2 | ENSMUST00000107692.2 | -0.1 | -0.11 | 0.63 |
| Arhgef7 | ENSMUST00000074856.7 | -0.2 | -0.21 | 0.63 |
| Kctd10 | ENSMUST00000102581.5 | -0.07 | -0.27 | 0.63 |
| Socs4 | ENSMUST00000065562.4 | -0.22 | -0.27 | 0.63 |
| Hnrnpu | ENSMUST00000037748.8 | 0 | -0.15 | 0.63 |
| Tbc1d4 | ENSMUST00000161991.2 | -0.3 | -0.39 | 0.63 |
| Ahcyl1 | ENSMUST00000029490.9 | -0.27 | -0.29 | 0.63 |
| Zfp385a | ENSMUST00000168828.1 | -0.28 | -0.28 | 0.63 |
| Tceb3 | ENSMUST00000030427.5 | -0.01 | -0.19 | 0.63 |
| Hnrnpa3 | ENSMUST00000111962.2 | -0.12 | -0.24 | 0.63 |
| Erc2 | ENSMUST00000090302.5 | -0.05 | -0.05 | 0.63 |
| Ncoa2 | ENSMUST00000081713.5 | -0.16 | -0.2 | 0.63 |
| Mtpn | ENSMUST00000031866.5 | -0.25 | -0.26 | 0.62 |
| Sec14l1 | ENSMUST00000090433.5 | -0.21 | -0.21 | 0.62 |
| Gk5 | ENSMUST00000122383.2 | -0.14 | -0.14 | 0.62 |
| Spop | ENSMUST00000107724.3 | -0.01 | -0.11 | 0.62 |
| Fut9 | ENSMUST00000084770.4 | -0.13 | -0.31 | 0.62 |
| Ank3 | ENSMUST00000182884.2 | -0.12 | -0.17 | 0.62 |
| Zfp831 | ENSMUST00000059452.5 | -0.26 | -0.26 | 0.62 |
| Kcnc3 | ENSMUST00000107907.3 | -0.07 | -0.07 | 0.62 |
| Jak2 | ENSMUST00000065796.4 | -0.31 | -0.43 | 0.62 |
| Snrk | ENSMUST00000120173.2 | -0.78 | -0.78 | 0.62 |
| Tsen54 | ENSMUST00000021134.4 | -0.41 | -0.41 | 0.61 |
| Bcl9l | ENSMUST00000074989.5 | -0.03 | -0.12 | 0.61 |
| Abce1 | ENSMUST00000080536.6 | -0.45 | -0.45 | 0.61 |
| Tet2 | ENSMUST00000098603.3 | -0.19 | -0.19 | 0.61 |
| Zfp217 | ENSMUST00000063710.7 | -0.1 | -0.11 | 0.61 |
| Ick | ENSMUST00000044551.7 | -0.74 | -1.5 | 0.61 |
| Smad4 | ENSMUST00000025393.8 | -0.07 | -0.21 | 0.61 |
| Chsy1 | ENSMUST00000036372.6 | -0.24 | -0.24 | 0.61 |
| Lpgat1 | ENSMUST00000110855.2 | -0.16 | -0.19 | 0.61 |
| Chek1 | ENSMUST00000172702.3 | -0.2 | -0.22 | 0.61 |
| Tex2 | ENSMUST00000042780.8 | -0.2 | -0.2 | 0.61 |
| Golga2 | ENSMUST00000113377.2 | -0.03 | -0.24 | 0.61 |
| Snx18 | ENSMUST00000109241.3 | -0.03 | -0.31 | 0.61 |
| Slc6a8 | ENSMUST00000033752.8 | -0.16 | -0.16 | 0.61 |
| Palm2Akap2 | ENSMUST00000150412.1 | -0.42 | -0.43 | 0.61 |
| Akap2 | ENSMUST00000102903.2 | -0.21 | -0.22 | 0.61 |
| Nbea | ENSMUST00000029374.6 | -0.23 | -0.23 | 0.6 |
| Gmfb | ENSMUST00000111817.2 | -0.12 | -0.23 | 0.6 |
| Rab3gap2 | ENSMUST00000069652.6 | -0.06 | -0.2 | 0.6 |
| Atf3 | ENSMUST00000027941.8 | -0.25 | -0.35 | 0.6 |
| Sirt1 | ENSMUST00000120239.2 | -0.24 | -0.25 | 0.6 |
| Rap2a | ENSMUST00000062117.7 | -0.43 | -0.44 | 0.6 |

**Supplementary Data 5. The predicted the potential targets of miR-135a-5p by miRDB.**

| Target Rank | Target Score | Gene Symbol |
| --- | --- | --- |
| 1 | 99 | Setbp1 |
| 2 | 99 | Cplx1 |
| 3 | 99 | Zfp951 |
| 4 | 98 | Rasal2 |
| 5 | 98 | Zbtb44 |
| 6 | 98 | Setd7 |
| 7 | 98 | Sdcbp |
| 8 | 98 | Ergic2 |
| 9 | 97 | Smim13 |
| 10 | 97 | Wscd2 |
| 11 | 96 | Kcnb1 |
| 12 | 96 | Shisa7 |
| 13 | 96 | Fcho2 |
| 14 | 96 | Rbak |
| 15 | 96 | Bsn |
| 16 | 95 | Clvs2 |
| 17 | 95 | Slc8a1 |
| 18 | 95 | Col5a1 |
| 19 | 95 | Acvr1b |
| 20 | 95 | Kdm7a |
| 21 | 95 | Cramp1l |
| 22 | 95 | Ubox5 |
| 23 | 95 | Cplx2 |
| 24 | 94 | Syt2 |
| 25 | 94 | Slc30a4 |
| 26 | 94 | Kcnj6 |
| 27 | 94 | Gulp1 |
| 28 | 94 | Zranb2 |
| 29 | 94 | Ntng1 |
| 30 | 94 | Dag1 |
| 31 | 94 | Dip2c |
| 32 | 94 | Ebf1 |
| 33 | 94 | Rsbn1l |
| 34 | 94 | 4930562C15Rik |
| 35 | 94 | Vps37c |
| 36 | 94 | Syne1 |
| 37 | 94 | Atp8a1 |
| 38 | 94 | Bach1 |
| 39 | 94 | Rnf138 |
| 40 | 93 | Esrra |
| 41 | 93 | Cttnbp2 |
| 42 | 93 | Kalrn |
| 43 | 93 | Slco5a1 |
| 44 | 93 | Orc5 |
| 45 | 93 | Mtmr2 |
| 46 | 93 | Atp11b |
| 47 | 93 | Slc25a5 |
| 48 | 93 | Slc9a3r2 |
| 49 | 93 | Entpd7 |
| 50 | 93 | Srsf3 |
| 51 | 93 | Ado |
| 52 | 93 | Wapl |
| 53 | 93 | Vamp2 |
| 54 | 93 | Pdp1 |
| 55 | 93 | Kdm1b |
| 56 | 93 | Trpc6 |
| 57 | 92 | Phldb2 |
| 58 | 92 | Kcnd1 |
| 59 | 92 | Rock2 |
| 60 | 92 | Cdyl2 |
| 61 | 92 | Erbb4 |
| 62 | 92 | Topors |
| 63 | 92 | Zfp131 |
| 64 | 92 | Hps5 |
| 65 | 92 | Trp63 |
| 66 | 92 | Ppp6r3 |
| 67 | 92 | C2cd2 |
| 68 | 91 | Mob1b |
| 69 | 91 | Slc16a6 |
| 70 | 91 | Ssr2 |
| 71 | 91 | Pitpnc1 |
| 72 | 91 | Ints2 |
| 73 | 91 | Lmtk2 |
| 74 | 91 | Runx2 |
| 75 | 91 | Cacna1e |
| 76 | 91 | Bmper |
| 77 | 91 | Pik3r2 |
| 78 | 91 | Mtss1 |
| 79 | 91 | Zfp518a |
| 80 | 91 | Mapkbp1 |
| 81 | 91 | Hif1an |
| 82 | 91 | Prune2 |
| 83 | 91 | Slc24a2 |
| 84 | 91 | Kcnn3 |
| 85 | 90 | Elk3 |
| 86 | 90 | Gria3 |
| 87 | 90 | Rnf43 |
| 88 | 90 | Ncln |
| 89 | 90 | Ildr2 |
| 90 | 90 | Chd1 |
| 91 | 90 | Slc12a6 |
| 92 | 89 | Abce1 |
| 93 | 89 | Washc4 |
| 94 | 89 | D17Wsu92e |
| 95 | 89 | Zfp654 |
| 96 | 89 | Bcl11a |
| 97 | 89 | Rab1b |
| 98 | 89 | Actr3b |
| 99 | 89 | Lats2 |
| 100 | 89 | Tti2 |
| 101 | 89 | Tstd2 |
| 102 | 89 | Kif3b |
| 103 | 89 | Rspo2 |
| 104 | 89 | Zfp292 |
| 105 | 89 | Cacna1d |
| 106 | 89 | Hmgxb3 |
| 107 | 89 | 1600012H06Rik |
| 108 | 89 | Atg14 |
| 109 | 88 | Klf4 |
| 110 | 88 | Wnt3 |
| 111 | 88 | Sema3e |
| 112 | 88 | Arhgap11a |
| 113 | 88 | Sec14l1 |
| 114 | 88 | Manea |
| 115 | 88 | Mfhas1 |
| 116 | 88 | Anxa7 |
| 117 | 88 | Slfn5 |
| 118 | 88 | Chmp4b |
| 119 | 88 | Hoxa10 |
| 120 | 88 | Ppp2r5c |
| 121 | 88 | Kcnab3 |
| 122 | 88 | Shisa6 |
| 123 | 88 | Ppp1r12c |
| 124 | 88 | Zcchc14 |
| 125 | 88 | Foxn3 |
| 126 | 88 | Fermt2 |
| 127 | 87 | Ntrk2 |
| 128 | 87 | Rgl1 |
| 129 | 87 | Jakmip2 |
| 130 | 87 | Tnpo1 |
| 131 | 87 | Zfp385b |
| 132 | 87 | Mypop |
| 133 | 87 | Creb5 |
| 134 | 87 | Fam71f1 |
| 135 | 87 | Gpr21 |
| 136 | 87 | Dpf1 |
| 137 | 87 | Man1a |
| 138 | 87 | Arhgap6 |
| 139 | 87 | Spock1 |
| 140 | 87 | Pde1a |
| 141 | 87 | Cpd |
| 142 | 87 | Strbp |
| 143 | 86 | Pcyt1b |
| 144 | 86 | Elovl6 |
| 145 | 86 | Slc9a9 |
| 146 | 86 | B3glct |
| 147 | 86 | Zdhhc6 |
| 148 | 86 | Fbln5 |
| 149 | 86 | Kctd1 |
| 150 | 86 | Med13 |
| 151 | 86 | Parn |
| 152 | 86 | Ndrg4 |
| 153 | 86 | Prlr |
| 154 | 86 | Ubfd1 |
| 155 | 86 | Psip1 |
| 156 | 85 | Aplf |
| 157 | 85 | Gng7 |
| 158 | 85 | Nr3c2 |
| 159 | 85 | Bace1 |
| 160 | 85 | Zbtb34 |
| 161 | 85 | Ccsap |
| 162 | 85 | Angptl2 |
| 163 | 85 | Ppp1r1c |
| 164 | 85 | Tbc1d4 |
| 165 | 85 | Net1 |
| 166 | 85 | Jade1 |
| 167 | 85 | Glrb |
| 168 | 85 | Taf4 |
| 169 | 85 | Snx18 |
| 170 | 84 | Pde8b |
| 171 | 84 | Naaladl2 |
| 172 | 84 | Hmbox1 |
| 173 | 84 | D630045J12Rik |
| 174 | 84 | Nbea |
| 175 | 84 | Kcnq5 |
| 176 | 84 | Thrb |
| 177 | 84 | Meltf |
| 178 | 84 | Chsy1 |
| 179 | 84 | Stk35 |
| 180 | 84 | Smad5 |
| 181 | 84 | Traf4 |
| 182 | 84 | Syt3 |
| 183 | 84 | Bzw2 |
| 184 | 84 | Cadm3 |
| 185 | 84 | Fgf11 |
| 186 | 84 | Entpd4 |
| 187 | 84 | Wdr33 |
| 188 | 84 | Dram2 |
| 189 | 83 | Edem3 |
| 190 | 83 | Casz1 |
| 191 | 83 | Rrbp1 |
| 192 | 83 | Sptbn1 |
| 193 | 83 | Nfxl1 |
| 194 | 83 | Atp2b3 |
| 195 | 83 | Mtmr12 |
| 196 | 83 | Ccdc92b |
| 197 | 83 | Cntnap1 |
| 198 | 83 | Zfp385a |
| 199 | 83 | Nedd9 |
| 200 | 83 | Ywhag |
| 201 | 83 | Sirt1 |
| 202 | 82 | Foxo1 |
| 203 | 82 | Kdm5b |
| 204 | 82 | Mtus1 |
| 205 | 82 | Ssr1 |
| 206 | 82 | Sv2b |
| 207 | 82 | Lmbrd2 |
| 208 | 82 | Arel1 |
| 209 | 82 | Dap |
| 210 | 82 | Pggt1b |
| 211 | 82 | Arhgef15 |
| 212 | 82 | Sema3a |
| 213 | 82 | Jak2 |
| 214 | 82 | Wdr45b |
| 215 | 82 | Mastl |
| 216 | 82 | Elovl7 |
| 217 | 82 | Arhgef4 |
| 218 | 82 | Ghsr |
| 219 | 82 | Sntb2 |
| 220 | 82 | Camk1g |
| 221 | 82 | 2310022B05Rik |
| 222 | 82 | Ralgapb |
| 223 | 81 | Gsk3b |
| 224 | 81 | Zfp831 |
| 225 | 81 | Saysd1 |
| 226 | 81 | Itm2b |
| 227 | 81 | Gabrb2 |
| 228 | 81 | Baz1a |
| 229 | 81 | 4921507P07Rik |
| 230 | 81 | Myocd |
| 231 | 81 | Trim66 |
| 232 | 81 | Col4a3 |
| 233 | 81 | Zfp322a |
| 234 | 81 | Evi5 |
| 235 | 81 | Slc5a7 |
| 236 | 81 | Mbnl1 |
| 237 | 80 | Arl5a |
| 238 | 80 | Zfp236 |
| 239 | 80 | Gad1 |
| 240 | 80 | Nampt |
| 241 | 80 | Plekhb2 |
| 242 | 80 | Bmpr1a |
| 243 | 80 | Tldc1 |
| 244 | 80 | Dnajc16 |
| 245 | 80 | Scn2a |
| 246 | 80 | Aebp2 |
| 247 | 80 | Unc5c |
| 248 | 79 | Sp9 |
| 249 | 79 | Rnf152 |
| 250 | 79 | Asph |
| 251 | 79 | Eya1 |
| 252 | 79 | Syt1 |
| 253 | 79 | Mapre2 |
| 254 | 79 | Snta1 |
| 255 | 79 | Gpr158 |
| 256 | 79 | Lrrn1 |
| 257 | 79 | Pik3cd |
| 258 | 79 | Rapgef6 |
| 259 | 79 | Mtdh |
| 260 | 79 | Efnb2 |
| 261 | 79 | Nefm |
| 262 | 79 | Inpp4b |
| 263 | 79 | Gas7 |
| 264 | 79 | Elk1 |
| 265 | 79 | Frmpd4 |
| 266 | 79 | Dyrk1b |
| 267 | 78 | Abhd15 |
| 268 | 78 | Arhgef6 |
| 269 | 78 | Mmp11 |
| 270 | 78 | Slc39a13 |
| 271 | 78 | Serpina1f |
| 272 | 78 | Arhgef2 |
| 273 | 78 | Clec4n |
| 274 | 78 | Fam81a |
| 275 | 78 | Ddx3x |
| 276 | 78 | Btaf1 |
| 277 | 78 | Glyctk |
| 278 | 78 | Nck1 |
| 279 | 77 | Cic |
| 280 | 77 | Tcf7l2 |
| 281 | 77 | Tmed5 |
| 282 | 77 | Ggnbp2 |
| 283 | 77 | Psd3 |
| 284 | 77 | Zfp143 |
| 285 | 77 | Sptbn4 |
| 286 | 77 | Usp15 |
| 287 | 77 | Btbd10 |
| 288 | 77 | Ptprf |
| 289 | 77 | Rps6kb1 |
| 290 | 77 | Bahcc1 |
| 291 | 77 | Frmd4a |
| 292 | 77 | Gpr155 |
| 293 | 77 | Rap1gds1 |
| 294 | 77 | Scn2b |
| 295 | 76 | Ifnk |
| 296 | 76 | Mras |
| 297 | 76 | Gstt3 |
| 298 | 76 | Il18rap |
| 299 | 76 | Rbfox2 |
| 300 | 76 | Usp42 |
| 301 | 76 | Zfp790 |
| 302 | 76 | Zfp960 |
| 303 | 76 | Ralgps2 |
| 304 | 75 | Elovl2 |
| 305 | 75 | Robo1 |
| 306 | 75 | Tmem189 |
| 307 | 75 | Nsdhl |
| 308 | 75 | Ralbp1 |
| 309 | 75 | Vipr2 |
| 310 | 75 | Zyg11b |
| 311 | 75 | Chst14 |
| 312 | 75 | Sertad2 |
| 313 | 75 | Rap2a |
| 314 | 75 | Lonrf1 |
| 315 | 75 | Klf13 |
| 316 | 75 | Ipo8 |
| 317 | 74 | Sec62 |
| 318 | 74 | Cdr2l |
| 319 | 74 | Adcyap1r1 |
| 320 | 74 | Ago2 |
| 321 | 74 | Chst3 |
| 322 | 74 | Faxc |
| 323 | 74 | Siah1a |
| 324 | 74 | Siah1b |
| 325 | 74 | Tmem9 |
| 326 | 74 | Mbnl2 |
| 327 | 74 | Trpc1 |
| 328 | 74 | Atp2b4 |
| 329 | 73 | Ublcp1 |
| 330 | 73 | Ccng2 |
| 331 | 73 | Pcyt1a |
| 332 | 73 | Sp3 |
| 333 | 73 | Clcn3 |
| 334 | 73 | Ror1 |
| 335 | 73 | G3bp1 |
| 336 | 73 | Krt33a |
| 337 | 73 | Pim2 |
| 338 | 73 | Hectd2 |
| 339 | 73 | Npat |
| 340 | 73 | Hdac9 |
| 341 | 73 | Ptpn3 |
| 342 | 73 | Zswim4 |
| 343 | 73 | Nucks1 |
| 344 | 73 | Pcnx2 |
| 345 | 73 | Dmrta1 |
| 346 | 72 | Aurkb |
| 347 | 72 | Pard3 |
| 348 | 72 | Aptx |
| 349 | 72 | Znrf1 |
| 350 | 72 | Ank3 |
| 351 | 72 | Abcb1b |
| 352 | 72 | Phtf2 |
| 353 | 72 | Pde1c |
| 354 | 72 | Slc4a4 |
| 355 | 72 | Dpt |
| 356 | 72 | Mal |
| 357 | 71 | Ndfip2 |
| 358 | 71 | Man2a1 |
| 359 | 71 | Myef2 |
| 360 | 71 | Ncoa2 |
| 361 | 71 | Syncrip |
| 362 | 71 | 1700037H04Rik |
| 363 | 71 | Atad1 |
| 364 | 71 | Spred1 |
| 365 | 71 | Josd1 |
| 366 | 71 | Fut9 |
| 367 | 71 | Ankrd55 |
| 368 | 71 | Snx12 |
| 369 | 70 | Rock1 |
| 370 | 70 | Stab2 |
| 371 | 70 | Clic4 |
| 372 | 70 | Chp2 |
| 373 | 70 | Camk2d |
| 374 | 70 | Vldlr |
| 375 | 70 | Zfp275 |
| 376 | 70 | Slc18a2 |
| 377 | 70 | Rnf26 |
| 378 | 70 | Tbl1xr1 |
| 379 | 70 | Utp14a |
| 380 | 70 | 2810021J22Rik |
