# Supplementary material for: miR-135a-5p mediates memory and synaptic impairments via the Rock2/Adducin1 signaling pathway in a mouse model of Alzheimer’s disease
Source: Nat Commun. 2021 Mar 26;12:1903. doi: 10.1038/s41467-021-22196-y (PMC7998005; doi:10.1038/s41467-021-22196-y)
Supplement: Supplementary file 4 — Reporting Summary [file 41467_2021_22196_MOESM4_ESM.pdf]

nature research

Corresponding author(s): Ling-Qiang Zhu
Last updated by author(s): Feb 14, 2021

Reporting Summary

Nature Research wishes to improve the reproducibility of the work that we publish. This form provides structure for consistency and transparency in reporting. For further information on Nature Research policies, see our Editorial Policies and the Editorial Policy Checklist.

Statistics

For all statistical analyses, confirm that the following items are present in the figure legend, table legend, main text, or Methods section.

n/a	Confirmed	
☐	☒	The exact sample size (*n*) for each experimental group/condition, given as a discrete number and unit of measurement
☐	☒	A statement on whether measurements were taken from distinct samples or whether the same sample was measured repeatedly
☐	☒	The statistical test(s) used AND whether they are one- or two-sided<br>*Only common tests should be described solely by name; describe more complex techniques in the Methods section.*
☐	☒	A description of all covariates tested
☐	☒	A description of any assumptions or corrections, such as tests of normality and adjustment for multiple comparisons
☐	☒	A full description of the statistical parameters including central tendency (e.g. means) or other basic estimates (e.g. regression coefficient) AND variation (e.g. standard deviation) or associated estimates of uncertainty (e.g. confidence intervals)
☐	☒	For null hypothesis testing, the test statistic (e.g. *F*, *t*, *r*) with confidence intervals, effect sizes, degrees of freedom and *P* value noted<br>*Give P values as exact values whenever suitable.*
☒	☐	For Bayesian analysis, information on the choice of priors and Markov chain Monte Carlo settings
☒	☐	For hierarchical and complex designs, identification of the appropriate level for tests and full reporting of outcomes
☐	☒	Estimates of effect sizes (e.g. Cohen's *d*, Pearson's *r*), indicating how they were calculated

Our web collection on statistics for biologists contains articles on many of the points above.

Software and code

Policy information about availability of computer code

Data collection	Single-, double-, or triple-labeling was viewed and imaged with a confocal laser-scanning microscope (Zeiss LSM780) and analyzed the fluorescence intensity with ImageJ software 1.52n in figure 1d-h, supplementary figure 1a,g-h and supplementary figure 3f-g. Brain tissues were cut with a vibrating microtome (Leica, Wetzlar, Germany) and was viewed and imaged with Nikon Ni-E in figure 4e-h, figure 6b-e, figure 7d-g, supplementary figure 4b-e and supplementary figure 6e-h. The Sholl analysis was used to determine the complexity of dendrites by using ImageJ plugin NeuronJ 1.4.3 in figure 4i, figure 6f, figure 7h, supplementary figure 4f and supplementary figure 6i. pClamp 10 and Med64 Mobius Software 0.5.1 for the collection of electrophysiological data in figure 4a-d, figure 6h-i, figure 7j-k, supplementary figure 1i-k, supplementary figure 4h-i, supplementary figure 4 and supplementary figure 6k-m. qRT-PCR was performed on CFX Connect Real-Time System 2.1 (BioRad, USA) by using SYBR Green Premix Ex Taq (Takara).The image of Western blot was scanned using Licor's Odyssey software 2.0. ImageJ 1.52n was used to quantify the western blot images.
Data analysis	SPSS 18.0 for data analysis and GraphPad Prism 6.0 for plotting; Zeiss LSM 780 for imaging; ClampFit 10.2 software (Molecular Devices) for supplementary figure 1i-k and Med64 Mobius Software 0.5.1 for the analysis of electrophysiological data(LTP and I-O curve); ImagJ software 1.52n for image and western blot images analysis.

For manuscripts utilizing custom algorithms or software that are central to the research but not yet described in published literature, software must be made available to editors and reviewers. We strongly encourage code deposition in a community repository (e.g. GitHub). See the Nature Research guidelines for submitting code & software for further information.

Data

Policy information about availability of data

All manuscripts must include a data availability statement. This statement should provide the following information, where applicable:

- Accession codes, unique identifiers, or web links for publicly available datasets
- A list of figures that have associated raw data
- A description of any restrictions on data availability

The source data for figures and supplementary figures of this manuscript are available in the source data file. Source data are provided with this paper. The databases used in this study were listed as below: TargetScan (http://www.targetscan.org/mmu_72/), miRDB (http://mirdb.org/), RegRNA2.0 (http://regrna2.mbc.nctu.edu.tw/index.html), LASAGNA (http://biogrid-lasagna.engr.uconn.edu/lasagna_search/), PROMO (http://alggen.lsi.upc.es/cgi-bin/promo_v3/promo/promoinit.cgi?dirDB=TF_8.3).

Field-specific reporting

Please select the one below that is the best fit for your research. If you are not sure, read the appropriate sections before making your selection.

☒ Life sciences ☐ Behavioural & social sciences ☐ Ecological, evolutionary & environmental sciences

For a reference copy of the document with all sections, see nature.com/documents/nr-reporting-summary-flat.pdf

Life sciences study design

All studies must disclose on these points even when the disclosure is negative.

Sample size	The sample size was determined according to previous studies (PMID: 31248451, PMID: 28111080, PMID: 26982728).
Data exclusions	There are instances when mice slices do not get staining efficiently with antibodies, possibly due to long-term storage and slice tissue inactivation. The analysis of such slices results in analytical bias, so a strict range of fluorescence characteristics must be defined for any particular longitudinal regeneration experiment. In other cases, the storage time of mouse brain protein or miRNA samples was different, which led to later results and analytical errors. After excluding some of these data, we will use the same batch of samples processed at the same time for accurate scientific experiments to eliminate errors.
Replication	All experiments were performed independently, by non-blinded investigators. We did the same experiments for at least three times in the manuscript and got similar results.
Randomization	We allocate the mice into different group with their strains and the virus injected. About the different group with same strain, we allocate them randomly before different virus injected. Cells in culture plates were allocated randomly into different groups.
Blinding	The investigators were double-blinded to group allocation during data collection and analysis about the behavior tests, qPCR, WB, sholl analysis, DCI, LTP, LTD, FISH/IF, Luciferase assay.

Behavioural & social sciences study design

All studies must disclose on these points even when the disclosure is negative.

Study description	*Briefly describe the study type including whether data are quantitative, qualitative, or mixed-methods (e.g. qualitative cross-sectional, quantitative experimental, mixed-methods case study).*
Research sample	*State the research sample (e.g. Harvard university undergraduates, villagers in rural India) and provide relevant demographic information (e.g. age, sex) and indicate whether the sample is representative. Provide a rationale for the study sample chosen. For studies involving existing datasets, please describe the dataset and source.*
Sampling strategy	*Describe the sampling procedure (e.g. random, snowball, stratified, convenience). Describe the statistical methods that were used to predetermine sample size OR if no sample-size calculation was performed, describe how sample sizes were chosen and provide a rationale for why these sample sizes are sufficient. For qualitative data, please indicate whether data saturation was considered, and what criteria were used to decide that no further sampling was needed.*
Data collection	*Provide details about the data collection procedure, including the instruments or devices used to record the data (e.g. pen and paper, computer, eye tracker, video or audio equipment) whether anyone was present besides the participant(s) and the researcher, and whether the researcher was blind to experimental condition and/or the study hypothesis during data collection.*
Timing	*Indicate the start and stop dates of data collection. If there is a gap between collection periods, state the dates for each sample cohort.*
Data exclusions	*If no data were excluded from the analyses, state so OR if data were excluded, provide the exact number of exclusions and the rationale behind them, indicating whether exclusion criteria were pre-established.*

Non-participation	*State how many participants dropped out/declined participation and the reason(s) given OR provide response rate OR state that no participants dropped out/declined participation.*
Randomization	*If participants were not allocated into experimental groups, state so OR describe how participants were allocated to groups, and if allocation was not random, describe how covariates were controlled.*

Ecological, evolutionary & environmental sciences study design

All studies must disclose on these points even when the disclosure is negative.

Study description	*Briefly describe the study. For quantitative data include treatment factors and interactions, design structure (e.g. factorial, nested, hierarchical), nature and number of experimental units and replicates.*
Research sample	*Describe the research sample (e.g. a group of tagged Passer domesticus, all Stenocereus thurberi within Organ Pipe Cactus National Monument), and provide a rationale for the sample choice. When relevant, describe the organism taxa, source, sex, age range and any manipulations. State what population the sample is meant to represent when applicable. For studies involving existing datasets, describe the data and its source.*
Sampling strategy	*Note the sampling procedure. Describe the statistical methods that were used to predetermine sample size OR if no sample-size calculation was performed, describe how sample sizes were chosen and provide a rationale for why these sample sizes are sufficient.*
Data collection	*Describe the data collection procedure, including who recorded the data and how.*
Timing and spatial scale	*Indicate the start and stop dates of data collection, noting the frequency and periodicity of sampling and providing a rationale for these choices. If there is a gap between collection periods, state the dates for each sample cohort. Specify the spatial scale from which the data are taken*
Data exclusions	*If no data were excluded from the analyses, state so OR if data were excluded, describe the exclusions and the rationale behind them, indicating whether exclusion criteria were pre-established.*
Reproducibility	*Describe the measures taken to verify the reproducibility of experimental findings. For each experiment, note whether any attempts to repeat the experiment failed OR state that all attempts to repeat the experiment were successful.*
Randomization	*Describe how samples/organisms/participants were allocated into groups. If allocation was not random, describe how covariates were controlled. If this is not relevant to your study, explain why.*
Blinding	*Describe the extent of blinding used during data acquisition and analysis. If blinding was not possible, describe why OR explain why blinding was not relevant to your study.*

Did the study involve field work? ☐ Yes ☐ No

Field work, collection and transport

Field conditions	*Describe the study conditions for field work, providing relevant parameters (e.g. temperature, rainfall).*
Location	*State the location of the sampling or experiment, providing relevant parameters (e.g. latitude and longitude, elevation, water depth).*
Access & import/export	*Describe the efforts you have made to access habitats and to collect and import/export your samples in a responsible manner and in compliance with local, national and international laws, noting any permits that were obtained (give the name of the issuing authority, the date of issue, and any identifying information).*
Disturbance	*Describe any disturbance caused by the study and how it was minimized.*

Reporting for specific materials, systems and methods

We require information from authors about some types of materials, experimental systems and methods used in many studies. Here, indicate whether each material, system or method listed is relevant to your study. If you are not sure if a list item applies to your research, read the appropriate section before selecting a response.

Materials & experimental systems

n/a	Involved in the study
☐	☒ Antibodies
☐	☒ Eukaryotic cell lines
☒	☐ Palaeontology and archaeology
☐	☒ Animals and other organisms
☐	☒ Human research participants
☒	☐ Clinical data
☒	☐ Dual use research of concern

Methods

n/a	Involved in the study
☒	☐ ChIP-seq
☒	☐ Flow cytometry
☒	☐ MRI-based neuroimaging

Antibodies

Antibodies used

Primary antibodies used:
Antibody Company Catalog No. Dilution
Anti-Actin Proteintech 60008-1-Ig 1:2000 WB
Anti-CaMKII Abcam ab22609 1:100 IF
Anti-GAD67 Millipore MAB5406 1:100 IF
Anti-Tau-5 Invitrogen AHB0042 1:1000 WB/1:200 IF
Anti-Rock2 Proteintech 21645-1-AP 1:1000 WB/1:100 IF
Anti-Rock1 Proteintech 21850-1-AP 1:1000 WB
Anti-Flag Proteintech 20543-1-AP 1:100 ChIP
Anti-Foxd3 ABclonal A2926 1:500 WB
Anti-Add1 Proteintech 10791-1-AP 1:1000 WB
Anti-Add2 Proteintech 14640-1-AP 1:1000 WB
Anti-Crmp2 Proteintech 14521-1-AP 1:1000 WB
Anti-Add1T445 Bioss bs-12961R 1:1000 WB
Anti-Add1S726 Bioss bs-6189R 1:1000 WB
Anti-Add2S713 Bioss bs-12960R 1:500 WB
Anti-Crmp2T555 ECM Biosciences CP2251 1:500 WB
Anti-Cacna1d ABclonal A15034 1:500 WB
Anti-Pik3R2 Bioss bs-4160R 1:500 WB
Anti-Limk1 ABclonal A16664 1:1000 WB
Anti-Limk2 Proteintech 12350-1-AP 1:1000 WB
Anti-Limk1T508 ABclonal AP0387 1:500 WB
Anti-Limk2T505 ABclonal AP0388 1:500 WB

Validation

All the Antibodies have been validated, Anti-Tau-5 (Invitrogen, Catalog: AHB0042, Reacts With: rat/ mouse/ bovine/ human/ sheep) has been validated in western blotting (PMID: 30699343, PMID: 30907729); Anti-Actin, Anti-Rock2, Anti-Rock1, Anti-Flag, Anti-Foxd3, Anti-Add1, Anti-Add2, Anti-Crmp2, Anti-Pik3R2, Anti-Add1T445, Anti-Add1S726, Anti-Add2S713, Anti-Crmp2T555, Anti-Cacna1d, Anti-Limk1, Anti-Limk2, Anti-Limk1T508 and Anti-Limk2T505 were also validated in databases or publications (PMID: 30582911, PMID: 30576711, PMID: 30597292, PMID: 28663280, PMID: 27127481, PMID: 30688376, PMID: 30824700, PubMed: 32013230, PMID: 27940916, PMID: 29970879, PMID: 23437279,); Anit-CaMKII, Anti-GAD67, Anti-Tau-5, Anti-Rock2 Antibodies have been validated for use on mouse brain sections or database (PMID: 30790215, PMID: 29136113, PMID: 24519931, PMID: 30005699, PMID: 25571974).

Eukaryotic cell lines

Policy information about cell lines

Cell line source(s)

Mouse N2a cells were bought from American Type Culture Collection(ATCC) bank, Manassas, VA, USA (CCL-131™). Human 293T cells were obtained from ATCC (Manassas VA, USA).

Authentication

morphology

Mycoplasma contamination

All cell lines tested negative for mycoplasma by MycAwayTM-Color One-Step Mycoplasma Detection Kit(40611ES25, yeasen).

Commonly misidentified lines
(See ICLAC register)

None of the used cell lines is listed in ICLAC database.

Palaeontology and Archaeology

Specimen provenance

Provide provenance information for specimens and describe permits that were obtained for the work (including the name of the issuing authority, the date of issue, and any identifying information).

Specimen deposition

Indicate where the specimens have been deposited to permit free access by other researchers.

Dating methods | *If new dates are provided, describe how they were obtained (e.g. collection, storage, sample pretreatment and measurement), where they were obtained (i.e. lab name), the calibration program and the protocol for quality assurance OR state that no new dates are provided.*

☐ Tick this box to confirm that the raw and calibrated dates are available in the paper or in Supplementary Information.

Ethics oversight | *Identify the organization(s) that approved or provided guidance on the study protocol, OR state that no ethical approval or guidance was required and explain why not.*

Note that full information on the approval of the study protocol must also be provided in the manuscript.

Animals and other organisms

Policy information about studies involving animals; ARRIVE guidelines recommended for reporting animal research

Laboratory animals	Male APP/PS1 mice of 4-month-old were purchased from the Jackson Laboratory (Bar Harbor, ME, NO. 034832). The male Tau-Knockout (Tau-/-) mouse of 6-month-old was generated using CRISPR-Cas9 system by specifically knocking out the sequence of exon 5 (Beijing Biocytogen Co., Ltd., Beijing, China). Adult male C57BL/6 mice of 6-month-old were purchased from the National Resource Center of Model Mice (Nanjing, China). In the behavioral experiment operation, gently touch the animal before catching them. If animals are killed, they will be dealt with at the Experimental Animal Central of Tongji Medical College, Huazhong University of Science and Technology.
Wild animals	No wild animals were used in the study.
Field-collected samples	No field collected samples were used in the study.
Ethics oversight	This study was approved by Institutional Animal Care and Use Committee of the Huazhong University of Science and Technology.

Note that full information on the approval of the study protocol must also be provided in the manuscript.

Human research participants

Policy information about studies involving human research participants

Population characteristics	All the human participants are adult male and aged between 81-90 years old. Group of cortical tissues from the brains of nondementia control subjects and AD cases based on neuropathological diagnosis in figure 5F-I and supplementary figure 1H (4CON vs 4AD) was obtained from the Tissue Bank of the Institute of Geriatrics, Chinese PLA General Hospital and Chinese PLA Medical Academy.
Recruitment	Participants were recruited as part of routine clinical AD. Self-selection bias is unlikely to have affected the results.
Ethics oversight	The present study was approved by the ethics committee of Tongji Medical College (Wuhan, China).

Note that full information on the approval of the study protocol must also be provided in the manuscript.

Clinical data

Policy information about clinical studies

All manuscripts should comply with the ICMJEguidelines for publication of clinical research and a completedCONSORT checklist must be included with all submissions.

Clinical trial registration	*Provide the trial registration number from ClinicalTrials.gov or an equivalent agency.*
Study protocol	*Note where the full trial protocol can be accessed OR if not available, explain why.*
Data collection	*Describe the settings and locales of data collection, noting the time periods of recruitment and data collection.*
Outcomes	*Describe how you pre-defined primary and secondary outcome measures and how you assessed these measures.*

Dual use research of concern

Policy information about dual use research of concern

Hazards

Could the accidental, deliberate or reckless misuse of agents or technologies generated in the work, or the application of information presented in the manuscript, pose a threat to:

No	Yes	
☐	☐	Public health
☐	☐	National security
☐	☐	Crops and/or livestock
☐	☐	Ecosystems
☐	☐	Any other significant area

Experiments of concern

Does the work involve any of these experiments of concern:

No	Yes	
☐	☐	Demonstrate how to render a vaccine ineffective
☐	☐	Confer resistance to therapeutically useful antibiotics or antiviral agents
☐	☐	Enhance the virulence of a pathogen or render a nonpathogen virulent
☐	☐	Increase transmissibility of a pathogen
☐	☐	Alter the host range of a pathogen
☐	☐	Enable evasion of diagnostic/detection modalities
☐	☐	Enable the weaponization of a biological agent or toxin
☐	☐	Any other potentially harmful combination of experiments and agents

ChIP-seq

Data deposition

☐ Confirm that both raw and final processed data have been deposited in a public database such as GEO.

☐ Confirm that you have deposited or provided access to graph files (e.g. BED files) for the called peaks.

Data access links<br>*May remain private before publication.*	*For "Initial submission" or "Revised version" documents, provide reviewer access links. For your "Final submission" document, provide a link to the deposited data.*
Files in database submission	*Provide a list of all files available in the database submission.*
Genome browser session<br>(e.g. UCSC)	*Provide a link to an anonymized genome browser session for "Initial submission" and "Revised version" documents only, to enable peer review. Write "no longer applicable" for "Final submission" documents.*

Methodology

Replicates	*Describe the experimental replicates, specifying number, type and replicate agreement.*
Sequencing depth	*Describe the sequencing depth for each experiment, providing the total number of reads, uniquely mapped reads, length of reads and whether they were paired- or single-end.*
Antibodies	*Describe the antibodies used for the ChIP-seq experiments; as applicable, provide supplier name, catalog number, clone name, and lot number.*
Peak calling parameters	*Specify the command line program and parameters used for read mapping and peak calling, including the ChIP, control and index files used.*
Data quality	*Describe the methods used to ensure data quality in full detail, including how many peaks are at FDR 5% and above 5-fold enrichment.*
Software	*Describe the software used to collect and analyze the ChIP-seq data. For custom code that has been deposited into a community repository, provide accession details.*

Flow Cytometry

Plots

Confirm that:

☐ The axis labels state the marker and fluorochrome used (e.g. CD4-FITC).

☐ The axis scales are clearly visible. Include numbers along axes only for bottom left plot of group (a 'group' is an analysis of identical markers).

☐ All plots are contour plots with outliers or pseudocolor plots.

☐ A numerical value for number of cells or percentage (with statistics) is provided.

Methodology

Sample preparation	*Describe the sample preparation, detailing the biological source of the cells and any tissue processing steps used.*
Instrument	*Identify the instrument used for data collection, specifying make and model number.*
Software	*Describe the software used to collect and analyze the flow cytometry data. For custom code that has been deposited into a community repository, provide accession details.*
Cell population abundance	*Describe the abundance of the relevant cell populations within post-sort fractions, providing details on the purity of the samples and how it was determined.*
Gating strategy	*Describe the gating strategy used for all relevant experiments, specifying the preliminary FSC/SSC gates of the starting cell population, indicating where boundaries between "positive" and "negative" staining cell populations are defined.*

☐ Tick this box to confirm that a figure exemplifying the gating strategy is provided in the Supplementary Information.

Magnetic resonance imaging

Experimental design

Design type	*Indicate task or resting state; event-related or block design.*
Design specifications	*Specify the number of blocks, trials or experimental units per session and/or subject, and specify the length of each trial or block (if trials are blocked) and interval between trials.*
Behavioral performance measures	*State number and/or type of variables recorded (e.g. correct button press, response time) and what statistics were used to establish that the subjects were performing the task as expected (e.g. mean, range, and/or standard deviation across subjects).*

Acquisition

Imaging type(s)	*Specify: functional, structural, diffusion, perfusion.*
Field strength	*Specify in Tesla*
Sequence & imaging parameters	*Specify the pulse sequence type (gradient echo, spin echo, etc.), imaging type (EPI, spiral, etc.), field of view, matrix size, slice thickness, orientation and TE/TR/flip angle.*
Area of acquisition	*State whether a whole brain scan was used OR define the area of acquisition, describing how the region was determined.*

Diffusion MRI ☐ Used ☐ Not used

Preprocessing

Preprocessing software	*Provide detail on software version and revision number and on specific parameters (model/functions, brain extraction, segmentation, smoothing kernel size, etc.).*
Normalization	*If data were normalized/standardized, describe the approach(es): specify linear or non-linear and define image types used for transformation OR indicate that data were not normalized and explain rationale for lack of normalization.*
Normalization template	*Describe the template used for normalization/transformation, specifying subject space or group standardized space (e.g. original Talairach, MNI305, ICBM152) OR indicate that the data were not normalized.*
Noise and artifact removal	*Describe your procedure(s) for artifact and structured noise removal, specifying motion parameters, tissue signals and physiological signals (heart rate, respiration).*

Volume censoring | *Define your software and/or method and criteria for volume censoring, and state the extent of such censoring.*

Statistical modeling & inference

Model type and settings | *Specify type (mass univariate, multivariate, RSA, predictive, etc.) and describe essential details of the model at the first and second levels (e.g. fixed, random or mixed effects; drift or auto-correlation).*

Effect(s) tested | *Define precise effect in terms of the task or stimulus conditions instead of psychological concepts and indicate whether ANOVA or factorial designs were used.*

Specify type of analysis: ☐ Whole brain ☐ ROI-based ☐ Both

Statistic type for inference
(See Eklund et al. 2016) | *Specify voxel-wise or cluster-wise and report all relevant parameters for cluster-wise methods.*

Correction | *Describe the type of correction and how it is obtained for multiple comparisons (e.g. FWE, FDR, permutation or Monte Carlo).*

Models & analysis

n/a	Involved in the study
☐	☐ Functional and/or effective connectivity
☐	☐ Graph analysis
☐	☐ Multivariate modeling or predictive analysis

Functional and/or effective connectivity | *Report the measures of dependence used and the model details (e.g. Pearson correlation, partial correlation, mutual information).*

Graph analysis | *Report the dependent variable and connectivity measure, specifying weighted graph or binarized graph, subject- or group-level, and the global and/or node summaries used (e.g. clustering coefficient, efficiency, etc.).*

Multivariate modeling and predictive analysis | *Specify independent variables, features extraction and dimension reduction, model, training and evaluation metrics.*
